# Accounting for electron-beam-induced warping of molecular nanocrystals in MicroED structure determination

**DOI:** 10.1107/S2052252524012132

**Published:** 2025-02-10

**Authors:** Niko Vlahakis, Arden Clauss, Jose A. Rodriguez

**Affiliations:** a Department of Chemistry and Biochemistry, UCLA-DOE Institute for Genomics and Proteomics; STROBE, NSF Science and Technology Center University of California, Los Angeles 611 Charles E. Young Dr East Los Angeles CA90095 USA; University of Oxford, United Kingdom

**Keywords:** cryoEM, MicroED, nanocrystals, beam-induced motion, radiation damage, 3D electron diffraction, 3DED, microcrystal electron diffraction, structural studies of nanocrystals, structural studies of microcrystals, TEM

## Abstract

High-energy electrons induce sample damage and motion at the nanoscale to fundamentally limit the determination of molecular structures by electron diffraction. Using a fast event-based electron counting (EBEC) detector, we characterize beam-induced, dynamic, molecular crystal lattice reorientations (BIRs). These changes are sufficiently large to bring reciprocal lattice points entirely in or out of intersection with the sphere of reflection, occur as early events in the decay of diffracted signal due to radiolytic damage, and coincide with beam-induced migrations of crystal bend contours within the same fluence regime and at the same illuminated location on a crystal. These effects are observed in crystals of biotin, a series of amino acid metal chelates, and a six-residue peptide, suggesting that incident electrons inevitably warp molecular lattices. The precise orientation changes experienced by a given microcrystal are unpredictable but are measurable by indexing individual diffraction patterns during beam-induced decay. Reorientations can often tilt a crystal lattice several degrees away from its initial position before irradiation, and for an especially beam-sensitive Zn(II)-me­thio­nine chelate, are associated with dramatic crystal quakes prior to 1 e^−^ Å^−2^ electron beam fluence accumulates. Since BIR coincides with the early stages of beam-induced damage, it echoes the beam-induced motion observed in single-particle cryoEM. As with motion correction for cryoEM imaging experiments, accounting for BIR-induced errors during data processing could improve the accuracy of MicroED data.

## Abstract 2

Here we identify and characterize the warping of molecular crystal lattices induced by electron beam exposure during microcrystal electron diffraction (MicroED/3DED) data collection. We find changes to consensus crystal lattice orientation that are often dramatic, and appear ubiquitous in small organic molecule crystals. This evidence highlights the relevance of crystal bending or warping as a consequence of radiation-induced damage on molecular specimens, and points to it as a fundamental source of error in MicroED/3DED data collection and structure determination.

## 1. Introduction

Electron diffraction patterns collected from crystals are expected to be largely unaffected by beam-induced translation, in contrast to the well known impact of this effect in high-resolution electron microscopy imaging (Henderson *et al.*, 2011 ▸; Brilot *et al.*, 2012 ▸; Li *et al.*, 2013 ▸; Scheres, 2014 ▸). However, crystal rotation, bending or warping can produce measurable changes in diffracted signal. Intentional rotation of a crystal, for instance, allows the efficient and sufficiently complete sampling of Bragg reflections in the reciprocal lattice, a prerequisite for structure determination (Shi *et al.*, 1998 ▸; Kolb *et al.*, 2007 ▸; Kabsch, 2010*a* ▸; Nederlof *et al.*, 2013 ▸; Nannenga *et al.*, 2014 ▸; Yonekura *et al.*, 2015 ▸). In electron diffraction measurements from 3D microcrystals, a target crystal is continuously and unidirectionally rotated about a single axis during data collection. Methods that exercise this approach, with or without cryogenic preservation of the crystal, are often termed microcrystal electron diffraction (MicroED) or 3D electron diffraction (3DED) (Shi *et al.*, 2013 ▸; Gemmi *et al.*, 2019 ▸; Saha *et al.*, 2022 ▸). Most MicroED data processing routines assume that a crystal remains relatively static on its support, even if it translates within the illuminating beam. If this assumption remains true, and the structure of a crystal is not significantly damaged by the incident beam, then the diffracted signal can be predicted, integrated and reduced to a list of intensities suitable for structure determination (Dorset, 1991 ▸; Hattne *et al.*, 2015 ▸; Shi *et al.*, 2016 ▸; Sawaya *et al.*, 2016 ▸; Van Genderen *et al.*, 2016 ▸).

At the energies used in conventional MicroED experiments (80–300 kV), incident electrons are expected to break chemical bonds (Egerton *et al.*, 2004 ▸), gradually reducing the degree of order within a crystal. This induces the decay of Bragg reflection intensities, first at fine spatial frequencies. The total electron beam fluence of a typical MicroED experiment (1–20 e^−^ Å^−2^) can fully ablate fine resolution diffraction (Hattne *et al.*, 2018 ▸). Many studies have measured and modeled these effects (Dorset & Turner, 1976 ▸; Henderson, 1995 ▸; Glaeser & Taylor, 1978 ▸; Kolb *et al.*, 2011 ▸; Hattne *et al.*, 2018 ▸; Saha *et al.*, 2024 ▸), which mirror those observed and investigated in detail in X-ray diffraction experiments where beam-induced damage is considered one of the most persistent obstacles to structure determination in beam-sensitive crystals even at cryogenic temperatures (Gonzalez & Nave, 1994 ▸; Nave & Garman, 2005 ▸; Garman & Weik, 2017 ▸). In addition, real-space changes in the appearance of crystal bend contours have been cataloged as evidence of electron beam damage, particularly in studies showing that diffraction contrast disappears from a crystalline region subjected to radiolysis (Murata *et al.*, 1977 ▸; Fryer, 1984 ▸).

Beam-induced anomalous changes in reflection intensities measured from static catalase crystals have also been observed by Bammes *et al.* (2010 ▸) at ultra-cold temperatures. In contrast to the anticipated decay in diffraction as a function of increased electron irradiation, non-monotonic changes in diffracted intensities were suggested to reflect a form of beam-induced specimen movement or charging. Recent pioneering work by Saha *et al.* (2024 ▸) has leveraged the unique spatial mapping capability of 4D scanning transmission electron microscopy to visualize domains of uniform orientation within a mosaic nanocrystal and track their changes as a result of beam exposure. That approach provided a fine-grained perspective on the phenomenon of radiation-induced changes in molecular crystal lattice structures. It revealed that the complex network of ‘coherently diffracting zones’ within an apparently single crystal is in constant motion in response to beam-induced radiolysis (Saha *et al.*, 2024 ▸). Such changes are also consistent with a model whereby already strained molecular crystals become subject to additional stress upon initiation of damage (McBride *et al.*, 1986 ▸), which may lead to further crystal bending that occurs alongside radiation-damage-induced lattice disruption by bond breaking. However, the impact of these effects on the routine determination of structures by MicroED has yet to be detailed.

Here, we further characterize correlated non-monotonic changes in Bragg reflection intensities observed in diffraction from static 3D molecular crystals in response to electron beam illumination, and investigate their impact on MicroED measurements and data quality. In real space, these changes are mirrored by the beam-induced movement of bend contours; these are visible as changing regions of differential diffraction contrast within crystals (Dorset, 1985 ▸; Pham *et al.*, 2023 ▸). We witness bend contours migrate across a crystal body in response to uniform illumination of a roughly 5 µm-diameter area: a manifestation of beam-induced changes in local bending within a mosaic nanocrystal. Ultimately, we find these complex rearrangements sum to an overall change in the consensus orientation of the lattice at a given crystal orientation, as measured by selected area electron diffraction (SAED) patterns. We refer to the consequence of this process on crystallographic measurements as ‘beam-induced reorientation’ (BIR) for the remainder of this paper. Our investigation leverages conventional TEM approaches in bright-field imaging and diffraction modes, confirming that this effect occurs when a crystal is uniformly illuminated with the electron beam as it would be in a conventional MicroED experiment. Ultimately, we highlight BIR as a fundamental phenomenon and a potential source of error in MicroED that would benefit from tracking and correction during data reduction.

## 2. Results

### 2.1. Non-monotonic decay occurs in electron diffraction from stationary microcrystals

**Figure 1:**
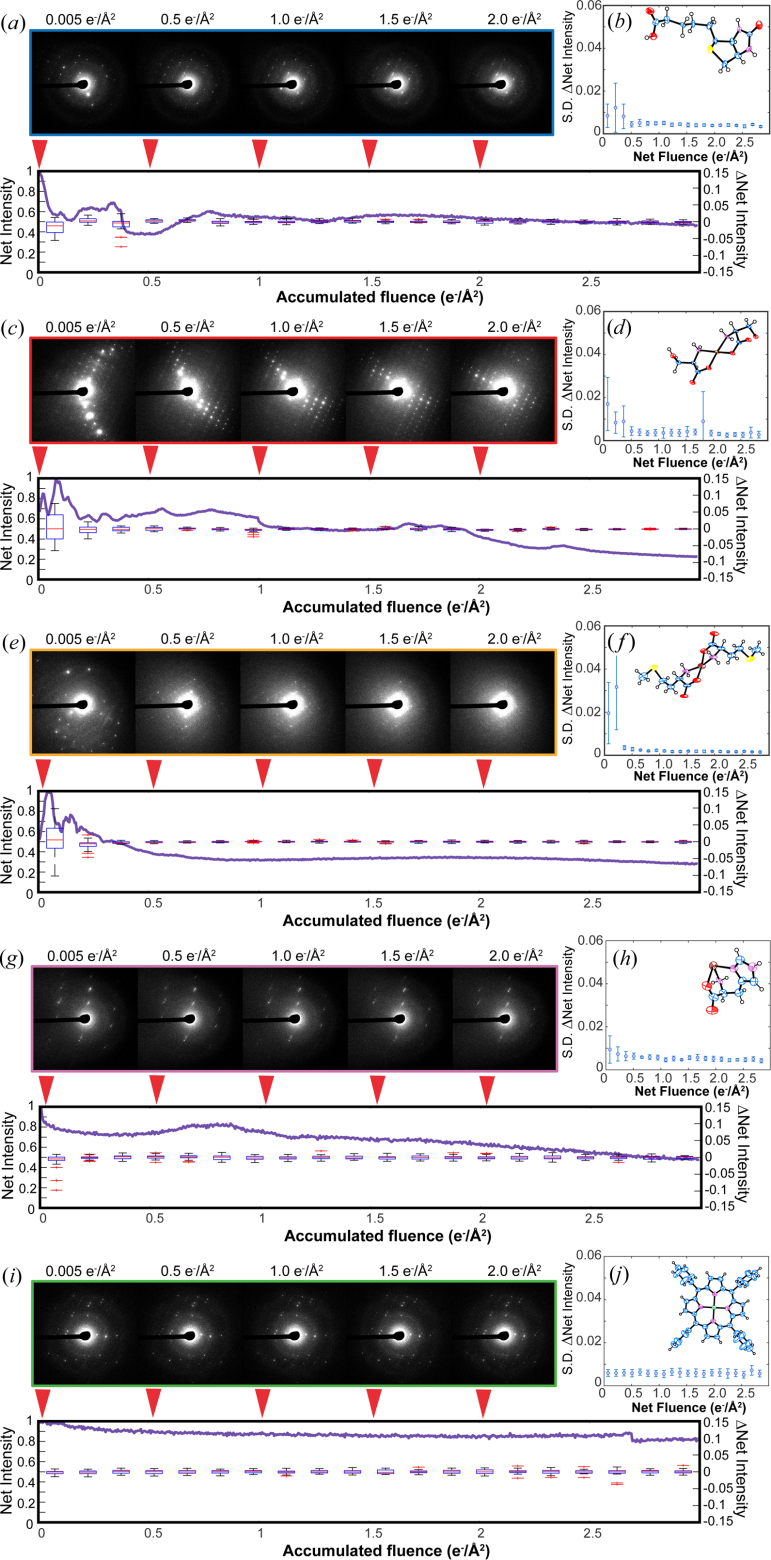
Non-monotonic changes in diffraction intensity from stationary, room-temperature crystals at 200 kV. For representative crystals of (*a*) biotin, (*c*) Cu(II)-serine, (*e*) Zn(II)-me­thio­nine, (*g*) Zn(II)-histidine and (*i*) Co(II)-porphyrin: initial diffraction pattern and frames acquired following an accumulated fluence of (from left to right) 0.5, 1.0, 1.5 and 2.0 e^−^ Å^−2^; plot of normalized total reflection intensity (left-hand *y* axis) as a function of accumulated fluence; and discretized plot of values of the derivative of this curve with respect to fluence (right-hand *y* axis). Plots of the average standard deviation of first-derivative values in each such discrete bin for (*b*) biotin, (*d*) Cu(II)-serine, (*f*) Zn(II)-me­thio­nine, (*h*) Zn(II)-histidine and (*j*) Co(II)-porphyrin interrogated at 200 kV and room temperature. Error bars are equal to one standard deviation from the mean. Inset are *ORTEP* diagrams of a representative atomic structure solution determined by MicroED for each compound.

When characterizing the anticipated monotonic decay of Bragg reflections in SAED patterns from molecular microcrystals, we observed anomalous fluctuations in Bragg reflection intensities arising from apparently static crystals. To measure these fluctuations, we illuminated static sub-micrometre-thick crystals of five distinct small molecules (biotin, Cu(II)-serine, Zn(II)-me­thio­nine, Zn(II)-histidine and Co(II)-meso-tetra­phenyl porphyrin) with low flux (0.01 e^−^ Å^−2^ s^−1^) 200 kV or (0.04 e^−^ Å^−2^ s^−1^) 300 kV electron beams at 293 K, while continuously recording selected area diffraction patterns from each. Across these dose series, static crystals exhibited the overall characteristic decay in Bragg reflections, with their rates of decay differing depending on their sensitivity to electron beam damage (Table S1 of the supporting information). Additionally, and notably, most crystals also displayed unpredictable non-monotonic changes in measured intensities for a subset of Bragg reflections across varying resolutions. The magnitude of this effect reflected the beam sensitivity of crystals; for the most sensitive, over the course of as little as 2 e^−^ Å^−2^ total exposure, reflections could newly enter or completely exit the excitation condition. The overall effect was distinct from the monotonic, resolution-dependent decay in reflection intensities due to radiolytic damage, as the likelihood of a reflection to exit excitation in this way appeared to be unrelated to its resolution (Fig. 1 ▸ and Fig. S1 of the supporting information).

The precise characteristics of these changes could not be predicted for a given crystal, but their magnitude showed a clear dependence on the identity of the compound in the crystal. Dose series acquired on stationary crystals of biotin, Cu(II)-serine and Zn(II)-me­thio­nine displayed high-amplitude non-monotonic fluctuations in reflection intensities with increasing fluence [Figs. 1 ▸(*a*)–1 ▸(*e*), Movies S1–S3 of the supporting information]. Equivalent dose series on Zn(II)-histidine displayed generally mild non-monotonic intensity fluctuations [Fig. 1 ▸(*g*), Movie S4], whereas beam-insensitive Co(II)-porphyrin crystals only exhibited minimal monotonic decay and no noticeable fluctuation at low electron doses [Fig. 1 ▸(*i*), Movie S5]. We noted, however, that when illuminated with as much as 100 e^−^ Å^−2^ total fluence at 200 kV, Co(II)-porphyrin crystals would likewise exhibit gradual anomalous intensity fluctuations of the same sort as the other compounds (Fig. S10), suggesting that beam-hardy molecular crystals are still prone to this behavior, albeit over a greater accumulation of beam fluence. These characteristic behaviors, shown for crystals illuminated at 200 kV in Fig. 1 ▸, were mirrored at 300 kV (Fig. S1). Though our experiments were standardly performed using crystals prepared on ultrathin carbon over lacey carbon supports, we observed equivalent behavior for crystals of these compounds prepared on a variety of other TEM grid support films, including extra thick carbon and formvar/carbon support films, reducing the likelihood that changes were an artifact of the choice of support film or grid type (Fig. S2). To rule out drifting of the sample stage as a root cause of the observed diffraction changes, control experiments were carried out in which multiple single diffraction patterns were collected on a stationary crystal separated by 1–2 min intervals with the beam blanked, followed by continuous illumination of the crystal and collection of a diffraction series. Non-monotonic intensity fluctuations, and the appearance of previously unmeasured reflections, only proceeded while the electron beam irradiated a crystal (Fig. S3). These observations collectively pointed to non-monotonic intensity changes being a beam-induced effect on the particular crystal illuminated. The crystal habits of the compounds studied spanned needles (biotin), thin rods [Cu(II)-serine], plates [Zn(II)-me­thio­nine and Co(II)-porphyrin] and prisms (Zn(II)-histidine), overall constituting a thorough coverage of the types of crystal morphologies commonly interrogated by MicroED.

### 2.2. Correlated changes in Bragg reflections are associated with non-monotonic decay in microcrystal diffraction

To characterize patterns of non-monotonic decay, we measured the total integrated intensity of all reflections across multiple dose series from different crystals (Fig. 1 ▸). The derivative in summed intensity for all measured reflections was calculated as a function of fluence, during increasing beam exposure. Derivative profiles are anticipated to maintain a near constant, negative value in the event of radiolytic damage-induced monotonic intensity decay. Instead, we observed varied behavior depending on the type of crystal studied. In crystals of biotin, Cu(II)-serine and Zn(II)-me­thio­nine, derivative profiles showed an initial period of dynamic fluctuation before approaching a constant negative value, where monotonic decay prevailed, most often by the time 0.5 e^−^ Å^−2^ had been delivered at 200 kV [Figs. S4(*a*)–S4(*i*)]. The distinction between these two regimes of intensity changes is evident in discretized plots of the derivative profiles, and led us to hypothesize that these crystals undergo an early period of dynamic change followed by global monotonic decay. In contrast, for the beam-resistant samples we studied, the hardiest of which was Co(II)-porphyrin, derivative profiles were consistently negative and near constant [Figs. S4(*m*)–S4(*o*)]. These trends were mirrored in plots of the standard deviation of derivative profile values averaged across multiple crystals of a given sample. When observed, the changes were consistently most prominent within the first 0.5 e^−^ Å^−2^ of irradiation at room temperature for beam-sensitive crystals [Figs. 1 ▸(*b*), 1 ▸(*d*), 1 ▸(*f*), 1 ▸(*h*) and 1 ▸(*j*)].

**Figure 2:**
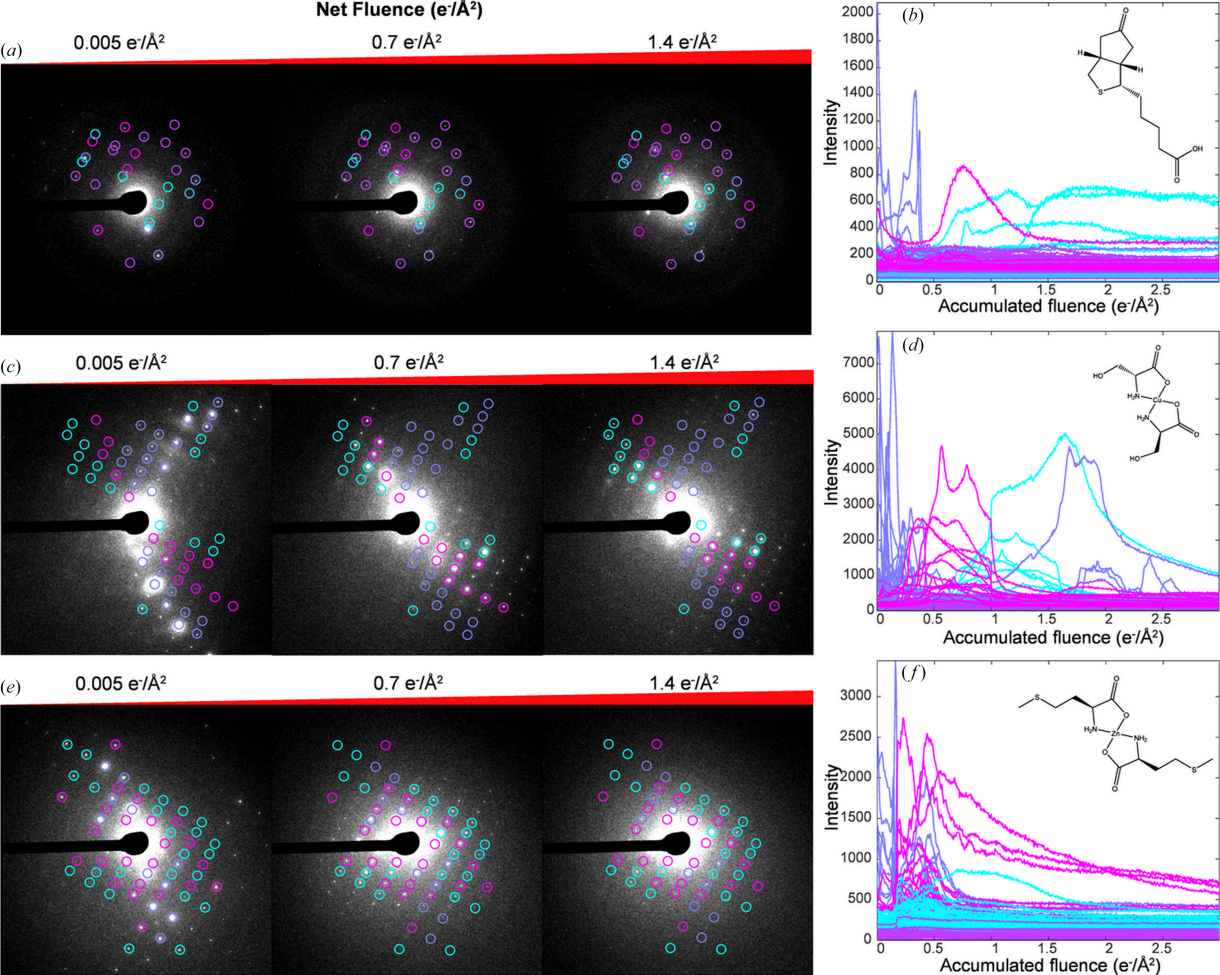
Correlated intensity changes of individual reflections in stationary crystals. (*a*) Initial diffraction pattern and frames acquired following an accumulated fluence of 0.7 and 1.4 e^−^ Å^−2^, and (*b*) plot of all measured reflection intensities as a function of fluence for a representative crystal of biotin. Traces are colored by *k*-means cluster, with matching colors annotating reflections in each major cluster on the diffraction frames. The same are shown for (*c*) and (*d*) Cu(II)-serine, and (*e*) and (*f*) Zn(II)-me­thio­nine, all at 200 kV and room temperature.

**Figure 3:**
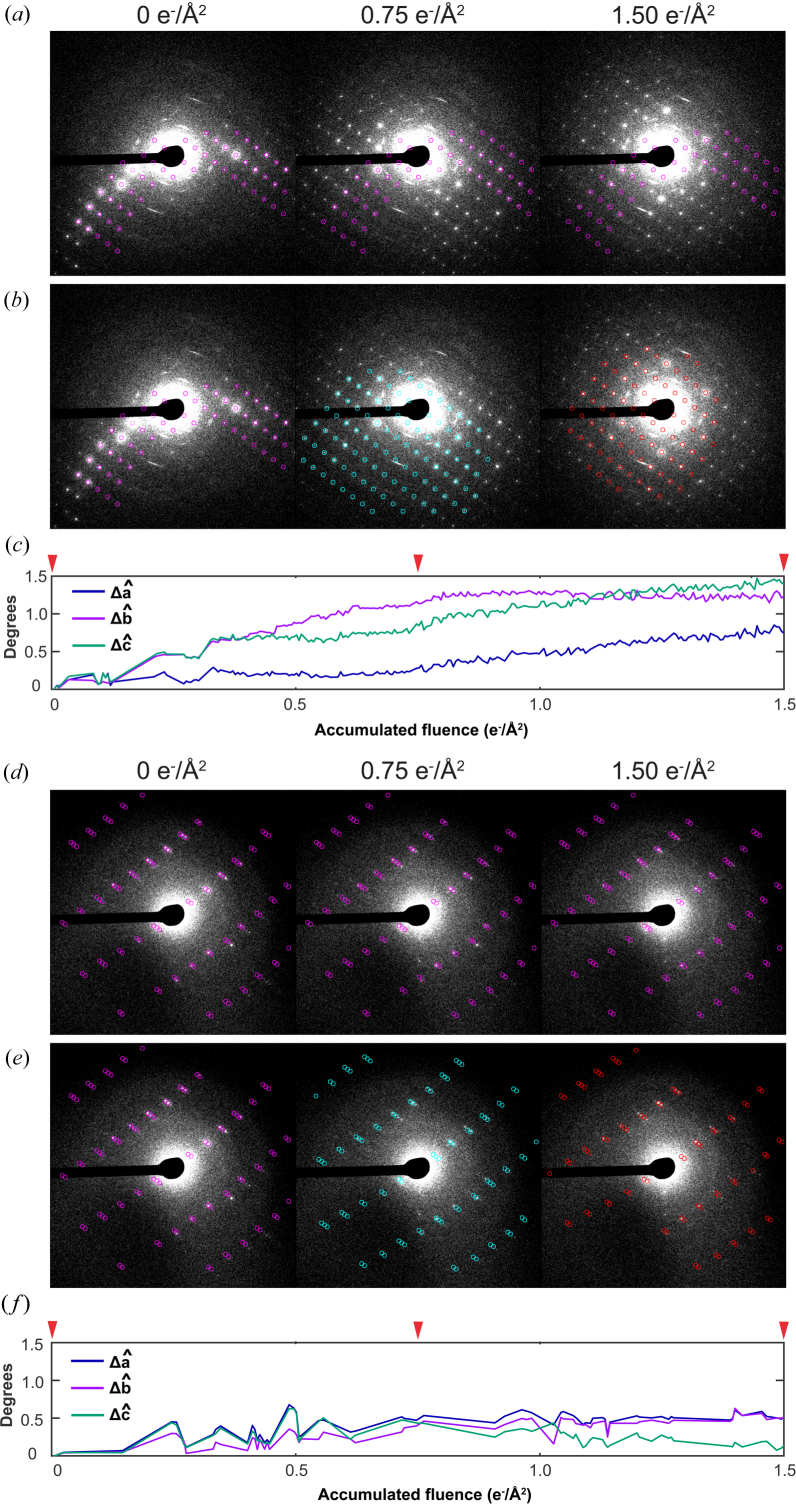
Tracking net crystal reorientation with serial crystallography data processing tools. (*a*) Diffraction frames from three points in a series on a stationary Cu(II)-serine crystal (200 kV, room temperature), overlaid with markers (magenta) at spot positions predicted for the orientation determined for the initial frame by *nXDS*. (*b*) Predicted spot positions from orientations determined for each frame individually, (magenta, teal and red markers), overlaid on the same three frames. (*c*) Calculated angular change in each unit-cell vector from its initial position as a function of fluence, and net rotation of the unit cell from its initial orientation following delivery of 1.50 e^−^ Å^−2^ accumulated fluence. (*d*)–(*f*) The same is shown for a stationary Zn(II)-histidine crystal, which undergoes less dramatic reorientation. Note fewer diffraction frames were successfully indexed in this case.

We then sought to quantitatively examine whether beam-induced diffraction changes arose from correlated, rather than random, changes in specific subsets of Bragg reflections. To achieve this, the intensity of each reflection was tracked as a function of fluence, and *k*-means clustering employed to classify reflections based on when in the diffraction series they were maximally excited, as illustrated for representative cases in Fig. 3. Crystals of Co(II)-porphyrin showed only minor, non-correlated fluctuations in the intensity of individual Bragg reflections, attributable to measurement error or noise, with minimal overall decay evident across the timescales of these experiments (Fig. S9). In contrast, dramatic changes in diffraction associated with crystals of Zn(II)-me­thio­nine were a consequence of abrupt and correlated changes in large sets of reflections. These abrupt events occurred in all crystals of Zn(II)-me­thio­nine measured at room temperature, always following the accumulation of 0.07–0.1 e^−^ Å^−2^ fluence. For some Zn(II)-me­thio­nine crystals, nearly all initially observed reflections disappeared, yielding an entirely new set of Bragg reflections [Figs. 2 ▸(*e*), 2 ▸(*f*) and S7]. All crystals surveyed exhibited diffraction changes between these two extremes. For example, crystals of Zn(II)-histidine showed gradual correlated changes in intensity of Bragg reflections, concomitant with a slow decay in overall signal (Fig. S8). In dose series from crystals of biotin and Cu(II)-serine, moderate fluctuations in intensities were often accompanied by the gradual appearance of clusters of reflections, and disappearance of others, in response to electron beam exposure. These correlated changes consistently marked the early phases of global decay in reflection intensities [Figs. 2 ▸(*a*)–2 ▸(*d*)].

### 2.3. Cryogenic temperatures prolong the period of anomalous beam-induced intensity fluctuations

To better understand the confounding role of beam-induced radiolysis on correlated non-monotonic fluctuations in Bragg reflections, we acquired dose series of stationary crystals at cryogenic temperatures. Crystals dry mounted on TEM grids were cooled to ∼100 K preceding data collection. Fig. S12 illustrates patterns of intensity decay for representative crystals of biotin [Figs. S12(*a*) and S12(*b*)], Zn(II)-me­thio­nine [Figs. S12(*c*) and S12(*d*)], Zn(II)-histidine [Figs. S12(*e*) and S12(*f*)] and Co(II)-porphyrin [Figs. S12(*g*) and S12(*h*)] (Figs. S12–S15 show this, and correlated fluctuations in reflection intensities, for additional crystals of each type, interrogated under the same conditions). Crystals that had shown anomalous correlated changes in Bragg reflections when illuminated at room temperature showed similar changes at 100 K, albeit to a milder extent than their room-temperature counterparts. However, the duration of anomalous intensity changes was generally more extended at 100 K, past the first 1.0 e^−^ Å^−2^ of beam exposure (Fig. S11).

The effect of temperature was especially noteworthy for crystals of Zn(II)-me­thio­nine, their sharp intensity changes observed at room temperature were not observed at 100 K. Instead, Zn(II)-me­thio­nine crystals at 100 K underwent smooth, prolonged periods of non-monotonic intensity fluctuation akin to those observed in other beam-sensitive samples at room temperature (Fig. S13). An interesting exception to this trend was noted in a select few crystals of Zn(II)-histidine and the Co(II)-porphyrin, which at 100 K exhibited more pronounced anomalous fluctuations than what was generally observed at room temperature (Fig. S16). These cases contribute to an increased mean fluctuation in values of the derivative of total intensity profiles for these compounds at 100 K with respect to crystals of the same type at room temperature (Fig. S11), though it should be noted that the typical behavior of these crystals at 100 K was to yield only very subtle or entirely absent non-monotonic fluctuations as fluence accumulated (Figs. S14 and S15). Apparent charging, showed by warping of the shape of the electron beam in diffraction mode, and visible bulk translations of crystals when illuminated, limited measurement of Cu(II)-serine crystals at 100 K. We therefore could not include Cu(II)-serine crystals interrogated at 100 K in our comparative analyses.

### 2.4. Tracking crystal orientation within microcrystal diffraction series links non-monotonic decay to BIR

The non-monotonic changes in Bragg reflections we measured are most consistently described by a reorientation of the overall reciprocal lattice detected in a crystal. We set out to measure the magnitude of change in net orientation for a given stationary crystal during a dose series by indexing individual frames in a dose series using the *nXDS* program (Kabsch, 2014 ▸). Since *nXDS* can operate on individual diffraction frames from randomly oriented crystals such as those regularly generated in serial crystallography experiments, we supplied series of frames from dose series to *nXDS* without prior knowledge of crystal orientation, but enforcing known space group and unit-cell constants (Tables S2–S6). Given the flatness of the Ewald sphere and extremely limited sampling of the reciprocal lattice in these still diffraction images, *nXDS* was not consistently able to yield indexing results for all frames in a dose series. However, we proceeded to analyze a subset of dose series, where the software could accurately index at least 50% of the frames acquired prior to the delivery of 1.5 e^−^ Å^−2^ fluence. We selected this cutoff upon noting that the likelihood of *nXDS* to successfully index sparse diffraction patterns was reduced as crystals accumulated radiation damage.

Best-fit orientation matrices for each frame were determined by *nXDS*, and orientation matrix components were compared for each frame in a dose series. *nXDS* would often find crystallographically equivalent orientations for different frames in the same series. We treated these symmetry-related orientations using the initial orientation as a reference and iteratively correcting each subsequent frame to its symmetry-equivalent orientation nearest that of the preceding frame. Frames were considered mis-indexed when any of their orientation matrix components differed from those of adjacent frames by more than two standard deviations of the population of all measured matrix components of that index. Such frames were excluded from further analysis. For beam-sensitive samples, where substantial non-monotonic intensity fluctuations had been measured, reflection positions predicted by *nXDS* matched reflections in the first frame of a series well, but agreed poorly with reflections on later frames. Figs. 3 ▸(*a*)–3 ▸(*c*) illustrates an example of these indexing disagreements for a room-temperature Cu(II)-serine crystal illuminated at 200 kV, which, following the delivery of only 1.5 e^−^ Å^−2^, produced reflections clearly inconsistent with its initial orientation. Nearby orientations, determined by *nXDS* indexing of subsequent frames in the series, produced predictions better matching the observed diffraction.

On the basis of *nXDS*-determined orientation matrices, we calculated the net electron-beam-induced angular change in unit-cell basis vectors for stationary crystals. From these, we could deduce the net rotation angle and axis of rotation best describing the coordinated changes in Bragg reflections as a function of dose. This analysis showed that the Cu(II)-serine crystal analyzed in Fig. 3 ▸ underwent a net 1.57° rotation over the course of 1.5 e^−^ Å^−2^ accumulated fluence. Such overall rotations were centered at apparently random axes, distinct from the basis vectors of the crystals’ unit cells, and could be obscured by inflating the mosaicity estimate in *nXDS*. For example, frames in the dose series from the Cu(II)-serine crystal in Fig. 3 ▸ could be indexed enforcing high mosaicity, such that the beam-induced change in orientation matrix components and therefore the net rotation of the crystal would appear negligible. As this outcome clearly does not match the reflection intensity changes measured for such a series, we enforced an initial mosaicity parameter that produced the best visual match between measured and predicted reflection locations, and kept this constant for all subsequent processing and analysis. Overall, the degree of intensity fluctuations correlated with the degree of crystal reorientation as calculated by *nXDS* and this analysis method over a given fluence. The Zn(II)-histidine crystal analyzed in Figs. 3 ▸(*d*)–3 ▸(*f*), for instance, only rotated 0.20° after accumulating approximately the same fluence as the Cu(II)-serine crystal. Such observations led us to the conclusion that, at least within this substrate scope, beam-sensitive crystals of different compounds are prone to differing degrees of BIR at both room and cryogenic temperatures.

### 2.5. Beam-induced motion of bend contours in real space mirrors reciprocal lattice BIR observed in diffraction patterns

**Figure 4:**
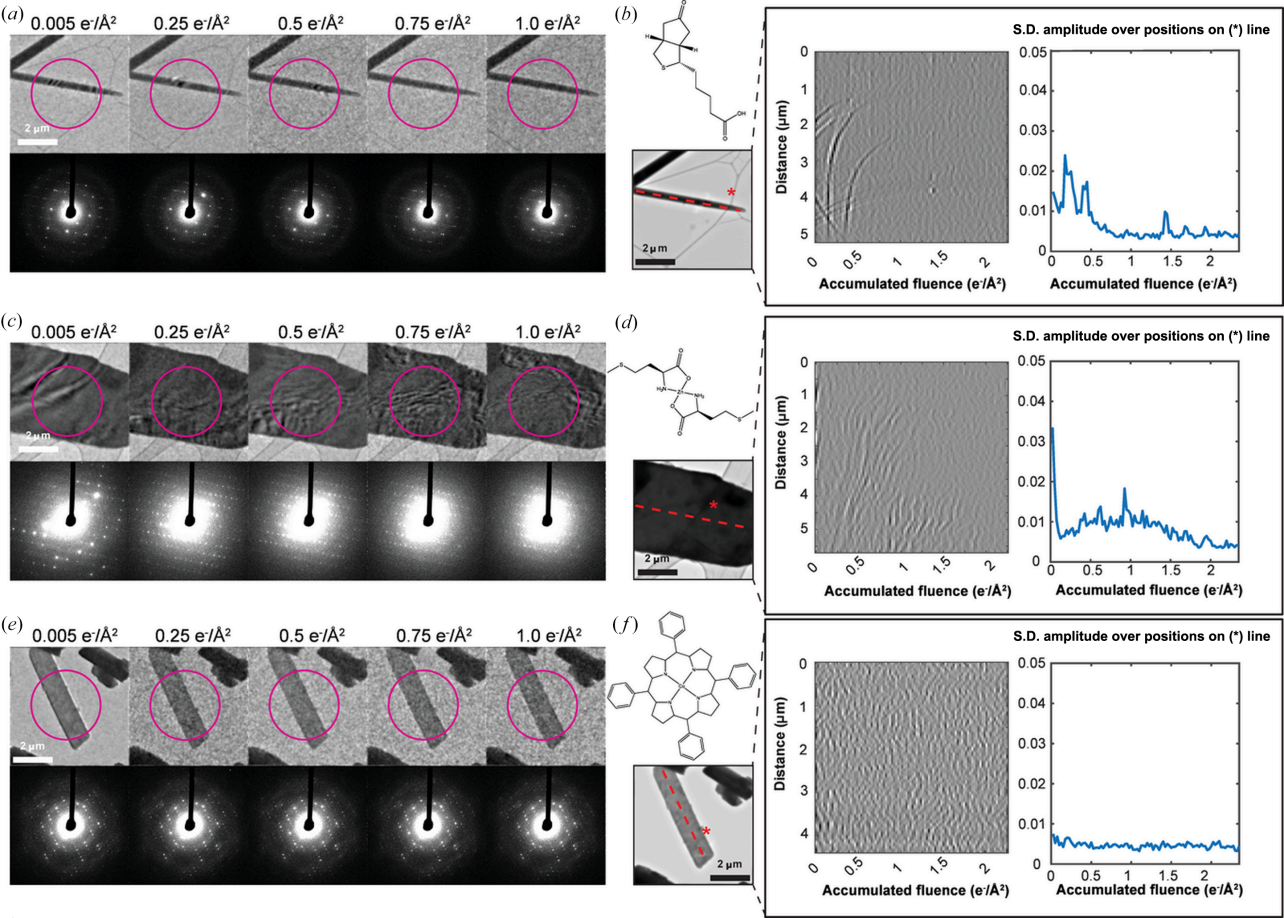
BIR showed by bend contour motion. Images of (*a*) biotin, (*c*) Zn(II)-me­thio­nine and (*e*) Co(II)-porphyrin nanocrystals following the delivery of increasing total fluence, alongside selected area diffraction patterns acquired immediately prior to each image. Following the application of a bandpass filter in the time dimension of each image stack, (*b*), (*d*) and (*f*) show projections of pixel intensities along manually defined directions (red-dashed line and * symbol) as a function of fluence, and plots of the standard deviation (S.D.) of distance in pixel value from the array mean over all pixel positions on the line as a function of fluence.

Given the evidence of BIR from diffraction series on beam-sensitive crystals, we hypothesized they might coincide with changes in diffraction contrast observable in real-space images of the illuminated crystals. To investigate this, we illuminated stationary crystals with the same flux used for diffraction measurements, while acquiring fast image series. At room temperature, crystals of biotin, Co(II)-porphyrin and Zn(II)-me­thio­nine showed clear bend contours across their bodies (Fig. 4 ▸), evidence of deformation visible as bands of differential contrast upon a crystal (Dorset, 1985 ▸). These contours moved unpredictably in response to electron beam exposure, but appeared to diminish in amplitude with increasing fluence. When illuminating crystals of biotin, we consistently measured bend contour migration that was greatest within the first 0.5 e^−^ Å^−2^ of exposure, and largely settled by or shortly after the delivery of 1.0 e^−^ Å^−2^ total fluence [Figs. 4 ▸(*a*) and 4 ▸(*b*), Movie S6]. Despite the dramatic motion of bend contours, no bulk motion of the crystal was seen over the course of an image series. In crystals of Zn(II)-me­thio­nine, the snap-like lattice change we discussed in earlier sections was corroborated by a rapid change in the position and appearance of bend contours in the crystal, which rippled and migrated rapidly, then abruptly vanished, succeeded by many less prominent, more slowly rippling contours [Figs. 4 ▸(*c*) and 4 ▸(*d*), Movie S7]. Crystals of Co(II)-porphyrin, interestingly, also hosted bend contours readily visible by imaging, but were distinctly static except in response to much higher electron flux [Figs. 4 ▸(*e*) and 4 ▸(*f*), Movie S8]. Notably, for each compound tested, the pattern of dynamism in bend contour motion matched the degree of lattice reorientation observed in diffraction patterns from the same illuminated area of the crystal. This further cemented the idea that bend contour motion and non-monotonic changes in diffraction intensities are complementary observations of the same dynamic bending phenomenon within molecular crystals as they are irradiated by an electron beam.

### 2.6. BIR impacts MicroED data collection and processing

Unanticipated BIR of otherwise stationary nanocrystals during MicroED experiments could introduce errors into data reduction routines adapted from X-ray crystallography, as these generally assume a crystal is rotating unidirectionally about a single known axis. To determine whether lattice reorientations have a significant impact on conventional MicroED datasets, we interrogated data recorded from continuously rotating crystals diffracted by a low-flux electron beam. We then searched for evidence of unmodeled consensus reorientation of continuously rotating crystals during data reduction.

**Figure 5:**
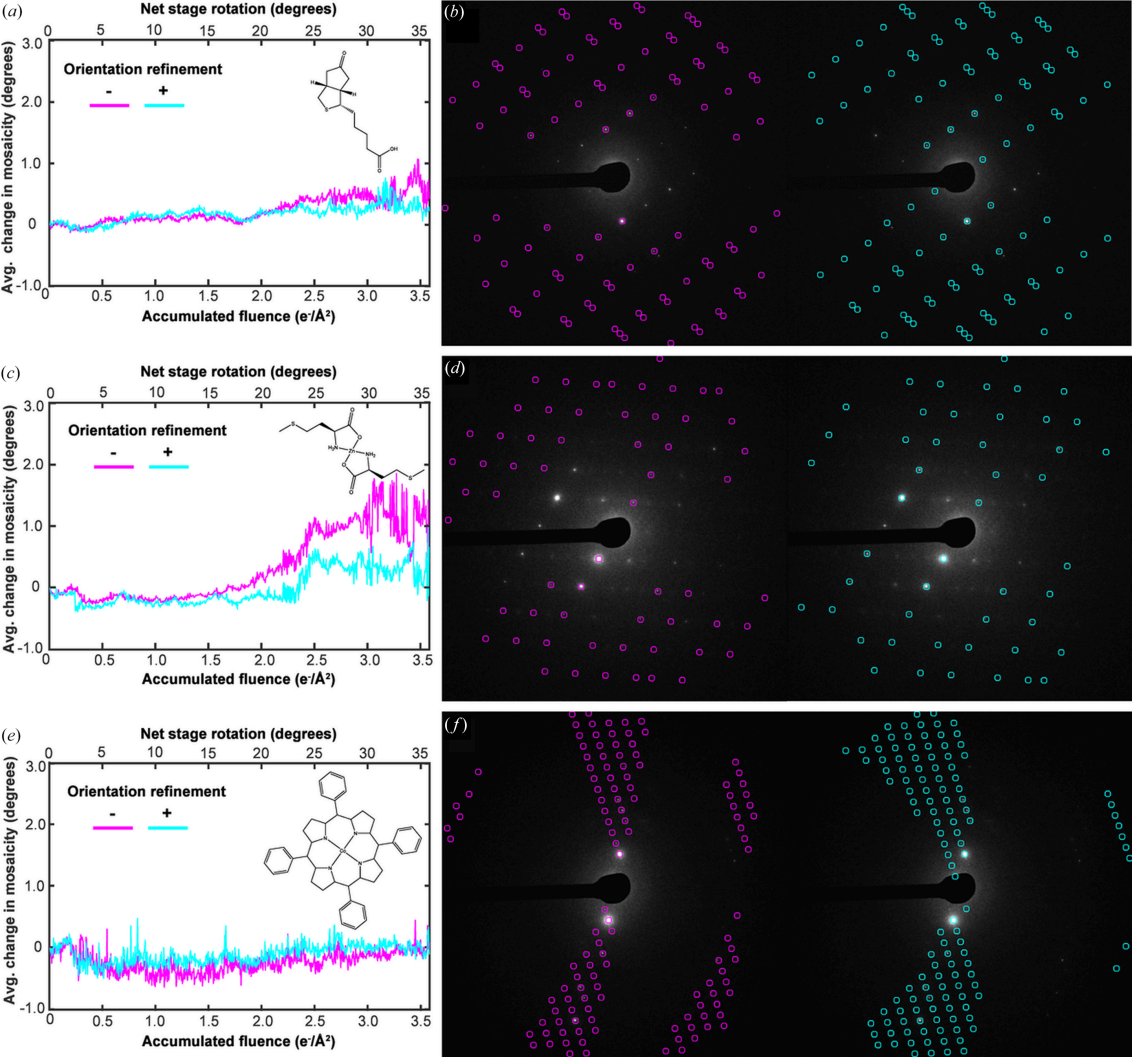
Impacts of BIR in MicroED data reduction. For (*a*) biotin and (*c*) Zn(II)-me­thio­nine crystals at 100 K, and (*e*) Co(II)-porphyrin crystals at room temperature all rotating at 0.09° s^−1^ during diffraction data acquisition: plots of the average change in mosaicity calculated with (light blue) and without (magenta) orientation refinements performed over all crystals of the type measured. For representative cases, (*b*), (*d*) and (*f*) show the diffraction frame acquired after delivery of 2.45 e^−^ Å^−2^ fluence and rotation of 24.5°, superimposed first with labels of predicted reflection positions determined by *XDS* without orientation refinements (magenta), and then with those determined with orientation refinements implemented (teal). Labels show all peaks whose centroids were calculated to be ±1° of rotation from the displayed frame in each case.

Data reduced from biotin and Zn(II)-me­thio­nine at 100 K and Co(II)-porphyrin at 293 K, in this case using *XDS* (Kabsch, 2010*a* ▸), revealed evidence that changes in net crystal orientation were occurring independent from the rotation of the sample stage (Figs. 5 ▸ and S20). After achieving initial indexing solutions, data were reprocessed holding certain geometric parameters often refined by *XDS* – unit-cell constants, rotation axis and orientation – fixed. We tracked the estimated crystal mosaicity determined by *XDS* for each frame of data. We expected that anomalous intensity fluctuations in diffraction tilt series might be interpreted by data reduction software as increased mosaicity, despite authentically being due to BIR. Supporting this hypothesis, the estimated mosaicity of Zn(II)-me­thio­nine and biotin crystals increased with accumulated fluence, as the crystal rotated. This was not true for Co(II)-porphyrin crystals diffracting under equivalent conditions, suggesting this might be a symptom of unmodeled orientation change. We then reprocessed the same tilt series datasets while allowing *XDS* to refine only crystal orientation in small batches of images. Performing these refinements over 2.5° wedges at a time somewhat abated the trend of inflated mosaicity, especially for data from Zn(II)-me­thio­nine crystals [Figs. 5 ▸(*a*)–5 ▸(*d*)]. Most of these Zn(II)-me­thio­nine datasets also granted decreased *R* factors when processed, allowing for orientation refinements; in one case decreases of as much as 7% in *R*_merge_ and 9% in *R*_meas_ were observed (Table S8). Following delivery of ∼1.75 e^−^ Å^−2^, we noted a generally improved agreement between reflection positions predicted by *XDS* and those observed in the experimental frame when orientation refinements were implemented for the Zn(II)-me­thio­nine tilt series datasets [Figs. 5 ▸(*b*), 5 ▸(*d*) and 5 ▸(*f*)]. While allowing crystal orientation to be refined over small wedges of data can reduce errors due to reorientation, MicroED data reduction may at times be limited by the minimum size of a wedge of angular data that can be refined.

### 2.7. Optimizing MicroED data collection in the face of BIR

Given the potential impact of BIR on MicroED data quality, particularly from beam-sensitive molecules, we sought to determine strategies for the optimal collection of MicroED data. To do so, we collected multiple subsequent datasets on single crystals, covering the same wedge of angular rotation repeatedly on each, with an incident flux of either 0.01 or 0.03 e^−^ Å^−2^ s^−1^ for a total of 90 s each [biotin and Zn(II)-histidine] or 100 s each [Zn(II)-me­thio­nine and Co(II)-porphyrin] and compared the data reduction statistics each yielded.

As we found the most dramatic evidence of BIR in the early regime of electron beam exposure, we were curious to explore if acquiring data following an initial period of illumination might yield improved data reduction quality. However, increasing total exposure of crystals to the electron beam is typically expected to worsen data reduction statistics by way of global intensity decay. This was corroborated by the general worsening of data reduction statistics, of structure density and of our likelihood to achieve a reasonable *ab initio* structure solution by direct methods, over subsequent sweeps of equivalent length at a dose rate 0.01 e^−^ Å^−2^ s^−1^ in data from six biotin crystals, and of 0.03 e^−^ Å^−2^ s^−1^ in data from five biotin crystals sampled at 100 K (Tables S10 and S11, Fig. S21).

For some crystals, a second sweep sometimes improved data reduction statistics. This was true of certain biotin and Zn(II)-me­thio­nine crystals diffracting at 100 K (Tables S10 and S12, Fig. S22), and certain Zn(II)-histidine crystals diffracting at room temperature (Table S13, Fig. S23), using our lowest dose rate of 0.01 e^−^ Å^−2^ s^−1^. Of the four Zn(II)-me­thio­nine crystals we interrogated under these conditions, half displayed this behavior. We rationalize these observations by noting that Zn(II)-me­thio­nine undergoes substantial BIR at 100 K during the first 1 e^−^ Å^−2^ of exposure, and that a second sweep may enable capture of a period less impacted by reorientations while still evading the onset of the most deleterious effects of beam-induced decay. In most biotin crystals at 100 K, which exhibit reorientations less dramatic than Zn(II)-me­thio­nine, data reduction statistics worsen monotonically over successive sweeps. We surmise, then, that overall reorientations in biotin crystals at 100 K are most often sufficiently mild that any advantage gained from acquiring data after they occur most distinctly is outweighed by the detrimental impact of global decay on data reduction statistics. This indicates a tradeoff between the effects of BIR and decay that occur as a given crystal accumulates electron beam fluence, which can be optimized for crystals of a given compound.

We therefore concluded that, if the crystals studied are beam sensitive and undergo modest BIR concurrently with MicroED collection, it is still advantageous to acquire a tilt series while delivering the lowest possible total fluence that affords measurement of the highest-resolution reflections the sample can produce. In contrast, data can be acquired from robust crystals after an initial period of illumination, affording improved reduction statistics and more accurate structure solutions. This tactic is sub-optimal, as it ignores data from the period of early beam exposure, when the crystal is best diffracting. Fortunately, existing indexing and integration software applicable to MicroED data include functionality, to varying degrees, for refinement of diffraction geometry parameters including crystal orientation within single continuous rotation datasets (Clabbers *et al.*, 2018 ▸; Palatinus *et al.*, 2019 ▸), and thus may be well suited to address these effects if leveraged.

### 2.8. BIR occurs, to varying degrees, in biomolecular crystal lattices

Though our initial analyses of BIR were performed on a series of small-molecule crystals, we were curious to assess the potential impact of reorientations on more challenging biomolecular targets. To do so, we interrogated microcrystals formed by peptides or proteins: the peptide AVAAGA, the peptidic macrocycle thio­strepton and the enzyme proteinase K. Due to the high solvent content often found within biomolecular crystal lattices, diffraction dose series in certain cases had to be collected from cryogenically preserved stationary crystals (Taylor & Glaeser, 1974 ▸). As such, while AVAAGA and thio­strepton crystals could be diffracted dry, proteinase K crystals had to remain hydrated, in a vitrified state. We found stationary AVAAGA peptide crystals to demonstrate evident non-monotonic intensity fluctuations while illuminated with the electron beam, similar in degree to those seen in biotin at 100 K. These crystals displayed the most notable evidence of BIR, followed by thio­strepton, and then proteinase K. Interestingly, crystals of proteinase K appeared to undergo no detectable reorientation during accumulation of 1 e^−^ Å^−2^ fluence. Curious to explore if this behavior was a result of the preservation of proteinase K crystals in vitreous ice, we proceeded to collect diffraction series on stationary, vitrified AVAAGA peptide crystals as well. However, these consistently showed a comparable degree of BIR to those diffracted dry, and a similar degree of beam-induced bend contour motion when imaged, ruling out a role for vitreous ice in fully quenching reorientations (Figs. S18 and S19, Movies S9 and S10).

## 3. Discussion

**Figure 6:**
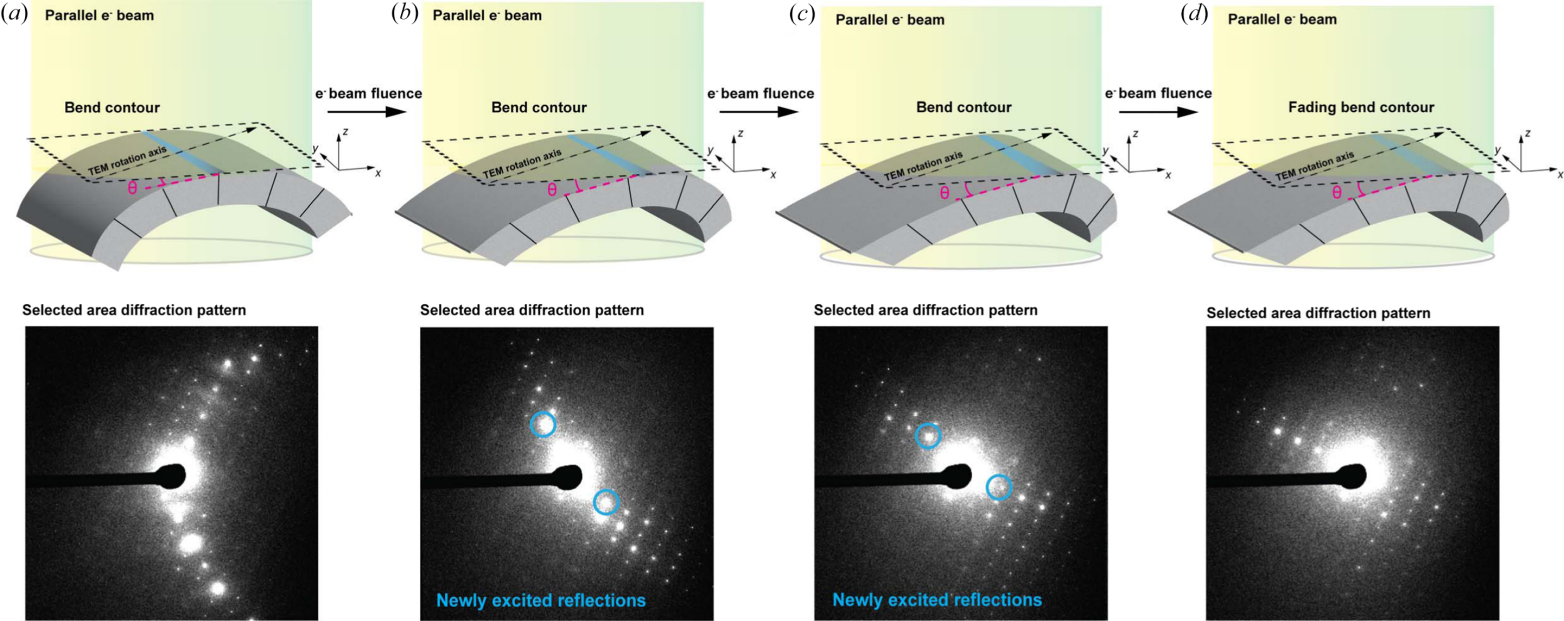
Schematic of BIR. As a static, imperfect, beam-sensitive molecular crystal is illuminated with a parallel beam in a TEM (*a*), pre-existing bends propagate and local bend angles (represented by θ in the illustration) change. As pictured, its warping is exaggerated and evidence of this warping is detectable both in diffraction-contrast imaging, where bend contours are seen to migrate along a crystal body, and in SAED, where the net orientation of the reciprocal lattice undergoes a change (*b*)–(*c*). For particularly beam-sensitive crystals, the set of excited Bragg reflections changes. The directions of these changes are effectively random with respect to the TEM rotation axis, such that if the crystal were rotating as in a MicroED experiment the deviation in net reciprocal lattice orientation would be non-trivial to predict. As electron beam fluence continues to be delivered to the crystal, both diffraction contrast in imaging mode and reflection intensity in diffraction mode gradually decay (*d*).

High-energy electron beams induce a myriad of changes to molecular crystals. Their impact on crystallinity by way of radiolytic damage is well recognized, and has been described as a resolution-dependent decrease in the intensity of Bragg reflections (Kolb *et al.*, 2010 ▸; Hattne *et al.*, 2018 ▸; Garman & Weik, 2023 ▸). However, some studies have also noted non-monotonic fluctuations in Bragg reflections during electron beam irradiation (Bammes *et al.*, 2010 ▸; Saha *et al.*, 2024 ▸). To further investigate the impact of these changes on molecular structure determination by MicroED, we charted the degree of non-monotonic fluctuation in SAED from static molecular nanocrystals. We found that Bragg reflections exhibit beam-induced, correlated and dose-dependent non-monotonic changes in intensity that correlate with crystal bend contour motion. These observations support a model in which electron beam irradiation directly induces dynamic bending or warping of a mosaic molecular crystal lattice (Fig. 6 ▸), which could be accounted for during MicroED data processing.

The link between electron beam irradiation and BIR is underscored by the fact that moderately beam-tolerant crystals such as those of Zn(II)-histidine exhibited limited intensity fluctuations that were gradual, akin to a slow out-of-plane rotation of the crystal. In contrast, the sharp, dramatic changes in diffraction of Zn(II)-me­thio­nine crystals when irradiated at room temperature at either 200 or 300 kV arise from rapid consensus reorientations, or crystal quakes. In fact, a dramatic peak is noted in derivative profiles of summed reflection intensity over time for these crystals, consistently appearing after exposure to 0.07–0.1 e^−^ Å^−2^, which is matched by a rapid shift in the appearance of bend contours in these crystals after accumulation of approximately the same integrated flux. These rapid changes might be explained by a buckling or partial collapse of the crystal lattice instigated by the delivery of a critical dose at room temperature, while application of cryogenic temperatures might allow the crystal to remain closer to its initial configuration. Crystal quakes were more rarely observed in crystals of Cu(II)-serine, Zn(II)-histidine and biotin held at room temperature, though went unobserved in any investigations at cryogenic temperature.

The occurrence of BIR in crystals at both room and cryogenic temperatures suggests the phenomenon is not induced by temperature or vitrification, or an artifact of interrogating specimens arrested in vitreous ice. This further challenges the notion that crystal reorientation in MicroED experiments results solely from a beam-induced crinkling effect on the sample support film, which is known to occur and expected to be accentuated in cryogenic experiments (Henderson & Glaeser, 1985 ▸; Glaeser *et al.*, 2011 ▸). Instead, our results support the well known protective effect of cryogenic temperatures on slowing of radiolytic damage-induced intensity decay in electron diffraction, and further link BIR to beam-induced damage. In contrast, vitrification in an aqueous matrix had little effect on lattice reorientation. For example, AVAAGA peptide crystals behaved similarly at 100 K whether or not they were vitrified. While we observed no such evidence of reorientation in proteinase K crystals, it is possible that damage accumulates too quickly in macromolecular crystals to allow for coherent beam-induced bending across entire nanocrystals. Instead, radiolytic beam damage might rapidly and globally decrease the degree of order within a protein crystal, quickly reducing excitation of higher-resolution reflections (Fig. S18).

Recent studies leveraging 4D-STEM instrumentation for nanobeam electron diffraction have illustrated the complex, imperfect substructures of molecular nanocrystal lattices (Gallagher-Jones *et al.*, 2019 ▸, 2020 ▸; Pham *et al.*, 2023 ▸). Importantly, those experiments have revealed the intricate motions of coherently diffracting zones within molecular crystals resulting from electron beam damage in 4D-STEM (Saha *et al.*, 2024 ▸). Our results corroborate those observations and extend them to TEM, as we see that bends in molecular crystal lattices propagate upon electron beam irradiation impacting diffraction from large regions of a nano- or microcrystal. Interestingly, an abundance of pre-existing bends does not guarantee BIR at low dose, as illustrated by the bend contours we visualized in beam-insensitive Co(II)-porphyrin crystals that did not migrate in response to 2 e^−^ Å^−2^ of electron beam exposure, but required much higher fluence to move. Instead, BIR appears linked to the inherent beam sensitivity of a nanocrystal, whose constituent misoriented domains move with respect to each other in response to beam exposure (Fig. 6 ▸), influenced by its degree of imperfection and internal strain prior to beam illumination.

BIR could contribute to the comparably poor data quality observed in MicroED data collected from some small-molecule nanocrystals compared with the X-ray diffraction data collected from their macroscopic counterparts. Net reorientations of 1–2° can be incorrectly interpreted by data reduction software as increased crystal mosaicity, which does not appropriately correct for the deviation in all reflection positions. Instead, the errors resulting from BIR can be partially accounted for by refinements to crystal orientation performed on small batches of images in the dataset. However, the degree to which these batches can be stably reduced is limited, depending on the processing software chosen. If too few reflections are considered during each refinement then additional errors may be incurred in the data reduction. In addition to crystal orientation refinements made possible with *XDS*, *DIALS* can correct errors in MicroED data associated with rotation instability and deviations from the crystal’s expected rotation angle (Waterman *et al.*, 2016 ▸; Winter *et al.*, 2018 ▸; Clabbers *et al.*, 2018 ▸). The frame-by-frame orientation estimations enabled during integration in *PETS2* (Palatinus *et al.*, 2019 ▸; Brázda *et al.*, 2019 ▸) can also be leveraged by practicing crystallographers to address BIR-associated errors. However, if BIR is sufficiently dramatic, such as for room-temperature Zn(II)-me­thio­nine crystals, data reduction statistics may suffer even if such tempered refinements are implemented. In extreme BIR cases, perhaps the broader application and further development of new serial crystallography approaches for indexing sparse patterns might offer more accurate indexing solutions (Kabsch, 2014 ▸; Brewster *et al.*, 2015 ▸; Hogan-Lamarre *et al.*, 2024 ▸). As such, we recommend careful consideration of BIR in MicroED experiments, and envision a potential approach for addressing these effects in the integration of serial crystallography indexing routines, such as those performed by *nXDS*, as a post-refinement correction implemented following traditional rotation crystallography indexing of a MicroED tilt series.

Overall BIR changes match the few degree reorientations observed for single molecules in single-particle cryoEM experiments (Henderson *et al.*, 2011 ▸), where beam-induced translation, rotation, warping and doming occur most dramatically within the first few e^−^ Å^−2^ of integrated flux (Glaeser, 2016 ▸). The BIR observed in MicroED experiments and the visible motion of bend contours observed in imaging experiments occurred most prominently within the same range of integrated flux values. We therefore anticipate that accounting for BIR during crystallographic data reduction will help improve the overall quality of MicroED data, as motion correction protocols have improved data quality for single-particle cryoEM.

## 4. Materials and methods

### 4.1. Electron beam fluence measurements

The electron beam fluence was measured at 200 and 300 kV by reproducing the condenser lens settings used for parallel beam illumination in diffraction mode, and in imaging mode. Gain-corrected, flat-field images of the parallel beam were collected on the Apollo detector, and the number of electrons measured per pixel was calculated from pixel values by applying the known conversion factor for the Apollo of 16 counts per electron event (Peng *et al.*, 2023 ▸). This yielded estimates of 0.01, 0.03 and 0.045 e^−^ Å^−2^ s^−1^ for spot sizes of 11, 10 and 9, respectively. On the Tecnai F30, where 300 kV measurements were collected, the microscope’s fluorescent screen was exposed to the parallel beam, and beam current measurements yielded estimates of 0.17 e^−^ Å^−2^ s^−1^ for a spot size of 9, 0.10 e^−^ Å^−2^ s^−1^ for a spot size of 10 and 0.04 e^−^ Å^−2^ s^−1^ for a spot size of 11. Data in this report, unless otherwise stated, were collected using the spot size 11 setting at either 200 or 300 kV.

### 4.2. Crystallization of samples

*Zn(II)-me­thio­nine*. Commercially acquired l-me­thio­nine was dissolved in 1 *M* NaOH solution at a 1:1 l-me­thio­nine to NaOH molar ratio. 2 ml of this solution was mixed with 10 ml of 0.1 *M* Zn(II) chloride solution and stirred for 1 h at room temperature. On addition of Zn(II) chloride, white solid immediately precipitated out. This precipitate was isolated by vacuum filtration, washed once with cold water and then with di­ethyl ether. Examination of the solid product under a light microscope revealed the material to be composed of small, colorless plate-shaped crystals.

*Zn(II)-histidine*. Zn(OH)_2_ was prepared by dissolving solid Zn(II) chloride in 1 *M* NaOH at a 1:2 molar ratio. This solution was stirred for 1 h at room temperature. Over this time, Zn(OH)_2_ had precipitated as a white solid, which was isolated by vacuum filtration, washed with cold water and air-dried overnight. 2 equiv. l-histidine and 1 equiv. Zn(OH)_2_ were dissolved in separate aqueous solutions, then mixed together and stirred at 50°C for 2 h. This solution was allowed to evaporate to 1/3 of its original volume and left at room temperature for crystals to form. Colorless, tetragonal crystals then formed overnight.

*Cu(II)-serine*. d-serine and Cu(II) sulfate were dissolved in 1 *M* NaOH at a molar ratio of 2:1:2 d-serine/Cu(II) sulfate/NaOH and stirred for 1 h at room temperature. The solution turned deep blue as the components dissolved. This solution was diluted to 1:20 in ethanol and left overnight, after which thin, blue, rod-shaped crystals had formed.

*Biotin*. A saturated solution of biotin was prepared in water heated to 100°C. The solution was allowed to slowly return to ambient temperature, during which colorless needle-shaped crystals formed. The crystal suspension was diluted to 1:10 in ethanol and saved for subsequent TEM sample preparation.

*AVAAGA peptide*. The AVAAGA peptide was ordered from Genscript, solubilized in water at 10 mg ml^−1^, and crystallized by the hanging drop method against 0.1 *M* Na citrate pH 6.5 and 10% ethanol.

*Thio­strepton*. 30 mg of commercially acquired thio­strepton was dissolved in 1.95 ml 24:1 chloro­form:isoamyl alcohol. 390 µl of ethanol and 195 µl of 100% glycerol were added and mixed into the solution. The solution was allowed to slowly evaporate at ambient temperature, and after a few days small tetragonal crystals had formed.

*Proteinase K*. Proteinase K was acquired from GoldBio, dissolved at 50 mg ml^−1^ in 50 m*M* HEPES pH 7.0, and crystals were grown by vapor diffusion in sitting drop trays against 1.2 *M* ammonium sulfate and 0.1 *M* Tris HCl pH 8.0 to achieve a needle-shaped polymorph amenable to producing high-resolution (2.0 Å) diffraction by MicroED.

### 4.3. TEM sample preparation

We chose to prepare dry TEM samples by methods typically used for modern conventional MicroED experiments, namely by either dispersing solid powder onto TEM grids or dropcasting crystals suspended in a solvent system we knew them to be insoluble in. Unless otherwise noted, ultrathin carbon on lacey carbon TEM grids with a copper mesh were used by default, though other support films were tested in control experiments to explore the effect of support film choice on BIR. Samples of Co(II)-porphyrin were prepared by crushing commercially acquired crystalline powders of the compounds between glass slides and dusting onto TEM grids. Samples of Cu(II)-serine and Zn(II)-histidine were prepared by crushing macroscopic crystals into powder between glass microscope slides and dusting onto TEM grids. Samples of biotin, thio­strepton and Zn(II)-me­thio­nine were prepared by suspending recrystallized material (in ethanol, for biotin and thio­strepton, and water for Zn(II)-me­thio­nine). TEM grids were prepared by dropcasting 2 µl of crystals in suspension onto the grid and wicking the excess solvent away until dry. Samples of AVAAGA were prepared by harvesting hanging drops filled with crystals, diluting twofold in water, and dropcasting 2 µl onto TEM grids and wicking excess solvent away until dry. To compare the degree of BIRs in dry samples to frozen-hydrated ones, cryoEM samples of the AVAAGA peptide were also prepared by dropcasting crystals diluted in water onto grids (both R2/1 Cu Quantifoil grids and extra-thick carbon on gold mesh grids were used for these tests, yielding equivalent results), then blotting off excess solvent and plunge-freezing at an FEI vitrobot. CryoEM samples of proteinase K crystals were prepared by dropcasting a crystal suspension diluted tenfold in 0.1 *M* Tris HCl pH 8.0 onto R2/1 Cu Quantifoil grids, then blotting and freezing at a vitrobot.

### 4.4. Electron diffraction data acquisition on stationary crystals

Electron diffraction data at 200 keV were acquired at a Talos F200C TEM equipped with an Apollo direct electron detector with a frame rate of 60 Hz (Peng *et al.*, 2023 ▸). During data acquisition, sets of 30 frames were integrated (2 frames per second effective rate) and binned to 2048 × 2048 images to produce frames in the movies used for further analysis in MRC file format. For each type of sample, well diffracting crystals were located using diffraction-mode settings focused away from the back focal plane to view a projection of the sample while delivering negligible fluence. For data acquisition, the microscope was operated in microprobe diffraction mode with a parallel beam, illuminating an area approximately 5 µm in diameter. All data were collected using a 100 µm selected area aperture, with a projected diameter of approximately 2 µm on the plane of the specimen. Diffraction movies on stationary crystals were acquired without any rotation of the TEM sample stage over a 5 min period for the small molecules studied, and a 2.5 min period for AVAAGA, thio­strepton and proteinase K.

Diffraction data using 300 kV electrons were acquired at a Tecnai F30 TEM equipped with a TVIPS TemCam XF416 CMOS detector, outputting diffraction movies in TVIPS file format. As on the Talos F200C microscope, crystals were first located using overfocused diffraction-mode settings and then illuminated for data acquisition with a parallel beam in microprobe diffraction mode with a 100 µm selected area aperture. Movies on stationary crystals were acquired using either 0.25, 1 or 2 s exposures on the TVIPS camera.

For data collection at 100 K, grids prepared with crystals were loaded dry into a cryo-transfer holder at room temperature, inserted into the microscope and then cooled to 100 K prior to data acquisition. For the proteinase K crystals, which would collapse if dried out prior to loading into the TEM, grids were only studied under frozen-hydrated conditions at 100 K. Crystals of the AVAAGA peptide were additionally studied under frozen-hydrated conditions to explore the impact of vitrification on BIR.

### 4.5. Slow continuous rotation electron diffraction data acquisition

To investigate the impact of BIR on diffraction tilt series a 0.09° s^−1^ rotation rate was used for each crystal. Data were acquired at the Talos F200C microscope at 200 kV, using the Apollo detector. Thirty native frames were integrated and binned to 2048 × 2048 arrays for an effective frame rate of 2 Hz in the final movies (0.045° oscillation per frame). Biotin and Zn(II)-me­thio­nine crystals were interrogated with this configuration at 100 K, while Co(II)-porphyrin crystals were interrogated at 293 K.

### 4.6. Fast event-based electron counting (EBEC) data collection for structure determination and multiple-sweep datasets

Tilt-series data collected for determination of representative structures of the compounds studied were acquired using the Talos F200C-DE Apollo setup, with a stage rotation rate of 1° s^−1^, over 60 to 100° wedges depending on the crystal. Sixty native frames were summed, such that each summed frame in the resulting tilt series represented a 1° oscillation of the sample within the beam [with the exception of two of the Zn(II)-me­thio­nine datasets used in that compound’s structure solution, acquired with a 3 frames s^−1^ rate and therefore 0.33° per frame oscillations]. Datasets were collected on multiple crystals of each compound in order to facilitate structure determination by merging datasets.

Using the same configuration detailed above, multiple subsequent sweeps of data were collected on individual crystals of biotin and Zn(II)-me­thio­nine at 100 K and Zn(II)-histidine and Co(II)-porphyrin at 293 K, where the same angular wedge of rotation was spanned for each crystal to assess the response of data reduction statistics from these series to increasing fluence. This was done at incident flux settings of either 0.01 or 0.03 e^−^ Å^−2^ s^−1^ (spot size 11 or 10).

The representative structure of Co(II)-porphyrin was determined using data collected from five crystals on the Tecnai F30, at 293 K, by rotating the stage 0.3° s^−1^ while acquiring 2 s exposures on the TVIPS camera.

### 4.7. Still diffraction series conversion and data analysis

Images were converted from MRC (from the Apollo camera) and TVIPS (from the TVIPS camera) format to SMV format using custom scripts developed in house and designed to run on *MATLAB* version 2023b. During this process, a spot-finding algorithm was used to detect peaks in diffraction series in a frame generated by applying a Gaussian filter with a one-element radius and taking the maximum intensity projection of the stack. Pixels exceeding 1.25× the intensity of the background (for the Apollo) and 2.5× (for the TVIPS XF416) after Gaussian filtration were masked with a diamond-shaped structured element centered at the detected peak and measuring 25 pixels from center to vertex. The total pixel intensity within each of these masked points was summed and recorded over each frame of each image series. The intensities of the brightest 20% of the reflections in each series, as judged by each reflection’s maximum intensity, wherever in the series it happened to occur, were summed to produce a 1D trace that varied with fluence. This trace was normalized to its maximum value and plotted as a function of accumulated fluence. The derivative with respect to fluence of this curve for each dataset was computed and plotted as a function of fluence as well. Individual reflection intensity traces were classified within a dataset by normalizing each to their maximum value and utilizing *k*-means clustering to sort them by the period in the dataset at which they were maximally excited, with five clusters specified to search for.

The output SMV stacks from the still crystal diffraction series were indexed using *nXDS* (Kabsch, 2014 ▸) with the known unit-cell parameters and space group of each crystal enforced, and orientation matrix elements were recorded from reflection files generated during peak integration (*INTEGRATE.HKL*). Ambiguities in orientation between settings deemed equally likely for a given frame by *nXDS* were resolved by taking advantage of the knowledge that each frame arose from successive exposures of the same crystal to arrive at a self-consistent set of orientations for each frame. This was done by considering the earliest determined orientation to be ground truth, and then iteratively changing the orientation determined for each subsequent frame to the symmetry-equivalent orientation nearest the previous one. Following this correction, if the difference in any orientation matrix component from the corresponding component in both the previous and the next frame (*i.e.a_x_* in frame 2 versus *a_x_* components in frame 1 and frame 3 of an image series) was greater than twice the standard deviation of all measured orientation matrix components of that index from that dataset, that frame was considered likely misindexed and discarded from further analysis. Following this, transformation matrices relating each orientation to the first frame’s orientation were calculated, which were subsequently converted to rotation axes and angles on a 3D Cartesian coordinate system describing the transformation experienced by the crystal orientation.

### 4.8. Processing and analysis of slow-rotation tilt series

*XDS* (Kabsch, 2010*b* ▸) was used to reduce data from slow-rotation tilt series, first without any refinements in the *IDXREF*, *INTEGRATE* or *CORRECT* steps permitted, and next with only refinements to crystal orientation permitted during these steps, to assess the impact on data reduction of accounting for unexpected changes in orientation. These refinements were performed on subsets of images in the series corresponding to 2.5° of rotation of the crystal. In both cases, a constant mosaicity of 0.2° was enforced. Data were scaled using *XSCALE* and data reduction statistics were recorded from the log files generated from this process (Kabsch, 2010*a* ▸). Additionally, estimated crystal mosaicity for each frame was recorded from the INTEGRATE.LP file (log file from the *XDSINTEGRATE* step), and plotted against accumulated fluence and TEM stage rotation for each data treatment.

### 4.9. Processing, analysis and structure determination from fast EBEC and 300 kV tilt series

All EBEC tilt series were indexed with *XDS*, allowing refinements to orientation, unit cell, direct beam position and rotation axis in the *IDXREF* and *CORRECT* steps, and direct beam position and orientation in the *INTEGRATE* step. All such refinements were performed over batches of images encompassing 5° of rotation. A high-resolution limit of 0.8 Å was enforced for integration. Data were scaled in *XSCALE* and data reduction statistics were recorded from the scaling output.

For successive tilt series acquired on the same crystals, the overall *R*_merge_ and *I*/σ were plotted and compared over the lifetime of each crystal. For individual datasets from biotin and Zn(II)-histidine, which routinely were sufficiently complete to yield a structure solution, phases were retrieved by direct methods using either *SHELXT* or *SHELXD* (Sheldrick, 2008 ▸), and refined in *SHELXLE* (Sheldrick, 2015 ▸) with the addition of riding hydrogen atoms and isotropic treatment of atomic displacement parameters (ADPs). The *R*_1_, *wR*_2_ and goodness of fit statistics after this treatment were recorded.

For the datasets used to determine and refine representative structures of each compound, either from 200 kV EBEC data or 300 kV data from the TVIPS camera, data were reduced and scaled as detailed above [though a high-resolution cutoff of 0.9 Å for integration was applied for 300 kV data from Co(II)-porphyrin], and multiple datasets were merged for each crystal as needed to improve completeness. Phases were retrieved by direct methods using either *SHELXT* or *SHELXD*, and refined in *SHELXLE* with the addition of riding hydrogen atoms and anisotropic treatment of ADPs, applying geometry restraints where needed for refinement to proceed stably. The *R*_1_, *wR*_2_ and goodness-of-fit statistics after this treatment were recorded, a CIF file saved, validated by the service *checkCIF* and deposited in the Cambridge Crystallographic Data Centre (CCDC) database.

### 4.10. Bend contour imaging and image analysis

Using the Talos F200C and DE Apollo detector, imaging-mode conditions at 200 kV were configured to match the flux and illuminated area size used to illuminate stationary crystals in diffraction mode. The condenser lenses were set to C1 = 41.317% and C2 = 45.449% at spot size 11, identical to those used for diffraction mode at spot size 11. A magnification of 4300× was selected, as at this magnification the beam under this lens configuration fully encompassed the area of the Apollo detector sensor. Low-dose presets were configured in serialEM, such that ‘Record’ mode loaded these imaging settings and ‘Search’ mode loaded the equivalent-flux diffraction-mode settings. Crystals dry-mounted on TEM grids at room temperature were located on a low-mag (155×) atlas, then imaged in cycles, where a single 1 s diffraction movie was collected followed by a 25 s imaging movie. In each mode, the detector was set to integrate 30 frames, such that each movie consisted of frames (2 per diffraction movie, 50 per imaging movie) integrating over 0.5 s exposures. Simple serialEM scripts were written to reconfigure the microscope optics and camera acquisition settings between each exposure while keeping the beam blanked at all times when not acquiring data. The beamstop and 100 µm selected area aperture were inserted manually prior to each diffraction movie. These were retracted, and a 100 µm objective aperture inserted and centered, prior to each imaging movie. Ten such ‘cycles’ were performed for each crystal studied.

The ten separate imaging-mode movies (saved in MRC file format) generated by the above approach for each crystal were read and compiled into the same 3D array to produce a pseudo time series, and analyzed using a custom script written in *MATLAB* version 2023b. Normalized cross-correlation-based alignment was performed between frames from different batches to reduce discontinuities present due to slight stage drift between successive imaging exposures. Stacks were then binned in all three dimensions by a factor of 5, and a low pass filter was applied in the time dimension. 2D seismograms were measured by manually defining a line in real space spanning the length of the crystal, and plotting pixel values along that line as a function of time. Intensity fluctuations at each position on this line as a function of time, or fluence, were measured by quantifying the absolute value difference between each pixel on the line and the mean pixel value of the stack with respect to time. The standard deviation of this fluctuation amplitude, over all pixel positions on the line, was plotted as a function of accumulated fluence.

## Supplementary Material

Crystal structure: contains datablock(s) biotin, CuIIserine, ZnIImethionine, ZnIIhistidine, CoIImesotetraphenylporphyrin. DOI: 10.1107/S2052252524012132/nf5001sup1.cif

Structure factors: contains datablock(s) biotin. DOI: 10.1107/S2052252524012132/nf5001biotinsup2.hkl

Structure factors: contains datablock(s) CuIIserine. DOI: 10.1107/S2052252524012132/nf5001CuIIserinesup3.hkl

Structure factors: contains datablock(s) ZnIImethionine. DOI: 10.1107/S2052252524012132/nf5001ZnIImethioninesup4.hkl

Structure factors: contains datablock(s) ZnIIhistidine. DOI: 10.1107/S2052252524012132/nf5001ZnIIhistidinesup5.hkl

Structure factors: contains datablock(s) CoIImesotetraphenylporphyrin. DOI: 10.1107/S2052252524012132/nf5001CoIImesotetraphenylporphyrinsup6.hkl

Movie S1. DOI: 10.1107/S2052252524012132/nf5001sup7.gif

Movie S2. DOI: 10.1107/S2052252524012132/nf5001sup8.gif

Movie S3. DOI: 10.1107/S2052252524012132/nf5001sup9.gif

Movie S4. DOI: 10.1107/S2052252524012132/nf5001sup10.gif

Movie S5. DOI: 10.1107/S2052252524012132/nf5001sup11.gif

Movie S6. DOI: 10.1107/S2052252524012132/nf5001sup12.gif

Movie S7. DOI: 10.1107/S2052252524012132/nf5001sup13.gif

Movie S8. DOI: 10.1107/S2052252524012132/nf5001sup14.gif

Movie S9. DOI: 10.1107/S2052252524012132/nf5001sup15.gif

Movie S10. DOI: 10.1107/S2052252524012132/nf5001sup16.gif

Supporting tables, figures, text, movie legends and checkCIF reports. DOI: 10.1107/S2052252524012132/nf5001sup17.pdf

CCDC references: 2349155, 2349156, 2349157, 2349158, 2349159

## Data Availability

Electron diffraction structures of the compounds studied here are available via CCDC deposition Nos: 2349155 (biotin), 2349156 [Cu(II)-serine], 2349157 [Zn(II)-histidine], 2349158 [Zn(II)-me­thio­nine] and 2349159 [Co(II)-porphyrin]. Diffraction data are available via Zenodo entries 10989028, 10989360, 10989502, 10989575, 10999587, 10995034, 10995139, 10999589, 10995169, 10993554, 10994067, 10994330, 10994691, 13308651, 10994067 and 11043930, listed in detail in Table S17. Any raw data or analysis scripts are available upon request from the authors.
