# Supplementary figures and images for: Accounting for electron-beam-induced warping of molecular nanocrystals in MicroED structure determination
Source: IUCrJ. 2025 Feb 10;12(Pt 2):223–38. doi: 10.1107/S2052252524012132 (PMC11878443; doi:10.1107/S2052252524012132)

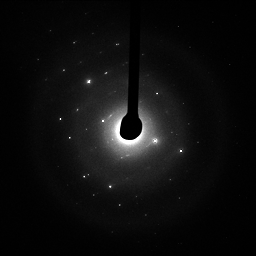

Supplement: Supplementary file 7 [file m-12-00223-sup7.gif]

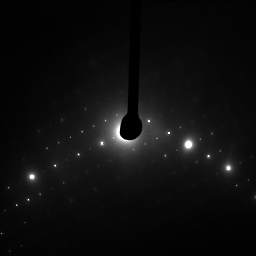

Supplement: Supplementary file 8 [file m-12-00223-sup8.gif]

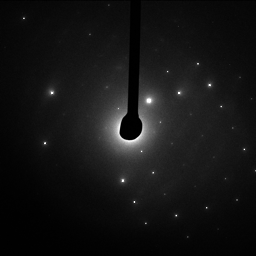

Supplement: Supplementary file 9 [file m-12-00223-sup9.gif]

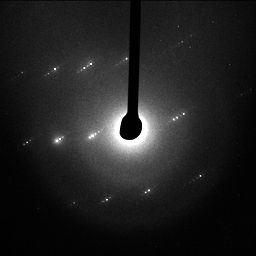

Supplement: Supplementary file 10 [file m-12-00223-sup10.gif]

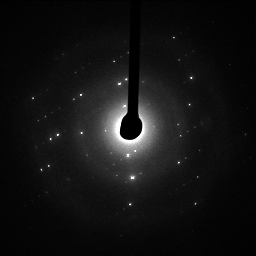

Supplement: Supplementary file 11 [file m-12-00223-sup11.gif]

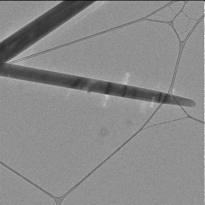

Supplement: Supplementary file 12 [file m-12-00223-sup12.gif]

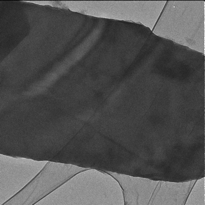

Supplement: Supplementary file 13 [file m-12-00223-sup13.gif]

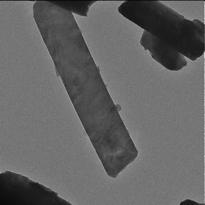

Supplement: Supplementary file 14 [file m-12-00223-sup14.gif]

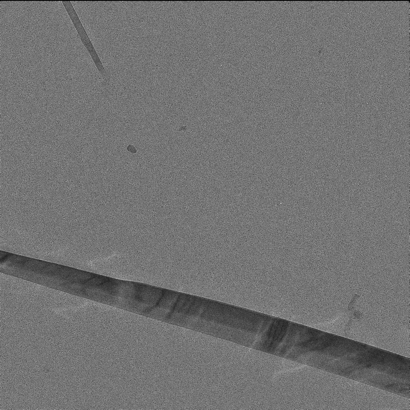

Supplement: Supplementary file 15 [file m-12-00223-sup15.gif]

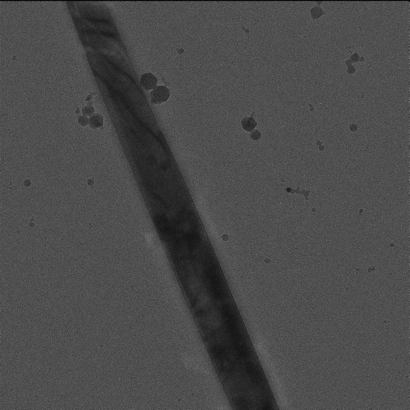

Supplement: Supplementary file 16 [file m-12-00223-sup16.gif]
