# Supplementary material for: Accounting for electron-beam-induced warping of molecular nanocrystals in MicroED structure determination
Source: IUCrJ. 2025 Feb 10;12(Pt 2):223–38. doi: 10.1107/S2052252524012132 (PMC11878443; doi:10.1107/S2052252524012132)
Supplement: Supplementary file 17 [file m-12-00223-sup17.pdf]

IUCrJ

Volume 12 (2025)

Supporting information for article:

Accounting for electron beam-induced warping of molecular nanocrystals in MicroED structure determination

Niko Vlahakis, Arden Clauss and Jose A. Rodriguez

Supplementary Materials for "Accounting for electron beam-induced warping of molecular nanocrystals in MicroED structure determination"

Niko Vlahakis[1], Arden Clauss[1], Jose A Rodriguez[1]*

[1]Department of Chemistry and Biochemistry; UCLA-DOE Institute for Genomics and Proteomics; STROBE, NSF Science and Technology Center; University of California, Los Angeles (UCLA); Los Angeles, CA 90095, USA.

*Correspondence to: Niko Vlahakis (nwvlahakis@g.ucla.edu) and Jose A. Rodriguez (jrodriguez@mbi.ucla.edu)

Supplementary Table 1. **Static microcrystalline samples diffracted by SAED under various environmental conditions**

Sample	No. crystals at 293 K and 200 kV	No. crystals at 100 K and 200 kV	No. crystals at 293 K and 300 kV	No. crystals at 293 K and 300 kV on extra-thick carbon supports	No. crystals at 100 K and 300 kV	τ at 200 keV and 293 K (e^-/Å^2)
Biotin	6	7	13	6	10	2.86
Cu(II) serine	6	N/A	12	N/A	N/A	2.27
Zn(II) methionine	6	10	11	6	8	0.85
Zn(II) histidine	5	6	8	6	5	6.11
Co(II) porphyrin	7 (3 additional for 100 e^-/Å^2 fluence series)	5	9	N/A	6	>100
AVAAGA peptide	4	5 (dry), 5 (frozen-hydrated)	4	N/A	5	~3
Thiostrepton	N/A	8	6	N/A	15	N/A
Proteinase K	N/A	5	N/A	N/A	N/A	N/A

The number of crystals interrogated at each temperature and electron beam energy is listed. τ is the average fluence to reduce diffraction intensity to $I_{max}/2$, deduced from the dose series yielded by these static crystals at 293 K, based on changes to the 20% brightest reflections detected from each diffraction series.

Supplementary Table 2. Crystallographic statistics for biotin

Empirical formula	C10 H16 N2 O3 S
Temperature	100 K
Wavelength	0.0251 Å
Detector	DE Apollo
Data processing	
No. crystals merged	4
Crystal system	Orthorhombic
Space group	$P2_12_12_1$
Unit cell dimensions:	
a, b, c (Å)	5.17, 10.11, 20.71
α, β, γ (°)	90.000, 90.000, 90.000
Volume (Å^3)	1083
Z	4
Resolution (Å)	9.05 – 0.8 (0.9 – 0.8)
No. observed reflections	15766
No. unique reflections	1194
R_{merge} (%)	18.8 (36.3)
R_{meas} (%)	19.7 (37.8)
Completeness (%)	92.5 (91.0)
I/σ	10.36 (7.28)
$CC_{1/2}$	98.5 (85.8)
Refinement	
R1 (%)	12.82
wR2 (%)	32.69
Goodness of fit	1.196

Statistics of crystallographic data reduction and refinement obtained during the determination of a structure from representative crystals of biotin.

Supplementary Table 3. Crystallographic statistics for Cu(II)-serine

Empirical formula	C6 H14 N2 O6 Cu
Temperature	100 K
Wavelength	0.0251 Å
Detector	DE Apollo
Data processing	
No. crystals merged	2
Crystal system	Monoclinic
Space group	$P2_1$
Unit cell dimensions:	
a, b, c (Å)	5.51, 8.24, 9.72
α, β, γ (°)	90.000, 90.330, 90.000
Volume (Å^3)	441
Z	2
Resolution (Å)	9.54 – 0.8 (0.9 – 0.8)
No. observed reflections	3266
No. unique reflections	844
R_{merge} (%)	13.2 (21.7)
R_{meas} (%)	15.2 (25.0)
Completeness (%)	88.4 (86.3)
I/σ	7.86 (5.76)
$CC_{1/2}$	97.9 (85.1)
Refinement	
R1 (%)	12.17
wR2 (%)	34.41
Goodness of fit	1.125

Statistics of crystallographic data reduction and refinement obtained during the determination of a structure from representative crystals of Cu(II)-serine.

Supplementary Table 4. Crystallographic statistics for Zn(II)-histidine

Empirical formula	C12 H16 N6 O4 Zn
Temperature	293 K
Wavelength	0.0251 Å
Detector	DE Apollo
Data processing	
No. crystals merged	1
Crystal system	Tetragonal
Space group	$P4_32_12$
Unit cell dimensions:	
a, b, c (Å)	7.09,7.09, 29.07
α, β, γ (°)	90.000, 90.000, 90.000
Volume ($Å^3$)	1461
Z	4
Resolution (Å)	7.27 – 0.8 (0.9 – 0.8)
No. observed reflections	5251
No. unique reflections	878
R_{merge} (%)	18.8 (43.4)
R_{meas} (%)	20.5 (46.9)
Completeness (%)	91.3 (91.2)
I/σ	6.04 (3.17)
$CC_{1/2}$	98.1 (64.8)
Refinement	
R1 (%)	16.54
wR2 (%)	41.90
Goodness of fit	1.191

Statistics of crystallographic data reduction and refinement obtained during the determination of a structure from representative crystals of Zn(II)-histidine.

Supplementary Table 5. Crystallographic statistics for Zn(II)-methionine

Empirical formula	C10 H20 N2 S2 Zn
Temperature	100 K
Wavelength	0.0251 Å
Detector	DE Apollo
Data processing	
No. crystals merged	4
Crystal system	Monoclinic
Space group	$P2_1$
Unit cell dimensions:	
a, b, c (Å)	9.07, 4.91, 14.97
α, β, γ (°)	90.000, 106.940, 90.000
Volume (Å^3)	638
Z	2
Resolution (Å)	8.82 – 0.8 (0.9 – 0.8)
No. observed reflections	13658
No. unique reflections	1294
R_{merge} (%)	23.4 (41.7)
R_{meas} (%)	24.6 (43.9)
Completeness (%)	83.8 (83.1)
I/σ	7.90 (5.13)
$CC_{1/2}$	95.2 (82.3)
Refinement	
R1 (%)	14.82
wR2 (%)	38.15
Goodness of fit	1.404

Statistics of crystallographic data reduction and refinement obtained during the determination of a structure from representative crystals of Zn(II)-methionine.

Supplementary Table 6. Crystallographic statistics for Co(II)-porphyrin

Empirical formula	C44 H28 N4 Co
Temperature	293 K
Wavelength	0.0197 Å
Detector	TVIPS XF416
Data processing	
No. crystals merged	5
Crystal system	Triclinic
Space group	$P\bar{1}$
Unit cell dimensions:	
a, b, c (Å)	9.60, 13.15, 13.28
α, β, γ (°)	79.530, 71.780, 69.390
Volume (Å^3)	1485
Z	2
Resolution (Å)	12.57 – 0.9 (1.0 – 0.9)
No. observed reflections	16550
No. unique reflections	4049
R_{merge} (%)	13.8 (56.5)
R_{meas} (%)	15.7 (64.3)
Completeness (%)	94.6 (96.1)
I/σ	5.47 (2.42)
$CC_{1/2}$	99.4 (87.4)
Refinement	
R1 (%)	16.12
wR2 (%)	45.20
Goodness of fit	1.589

Statistics of crystallographic data reduction and refinement obtained during the determination of a structure from representative crystals of Co(II)-meso-tetraphenyl porphyrin.

Supplementary Table 7. Crystallographic statistics from slowly rotating biotin crystals

Crystal	Crystal 1	Crystal 1	Crystal 2	Crystal 2
XDS orientation treatment	Fixed	Refined	Fixed	Refined
Resolution (Å)	10.11 – 0.8 (0.9 – 0.8)	10.11 – 0.8 (0.9 – 0.8)	10.11 – 0.8 (0.9 – 0.8)	10.11 – 0.8 (0.9 – 0.8)
No. observed reflections	1727	1703	1716	1666
No. unique reflections	515	517	613	598
R_{merge} (%)	12.0 (33.7)	14.0 (37.2)	11.7 (18.2)	10.9 (17.4)
R_{meas} (%)	14.2 (40.2)	16.7 (44.1)	14.4 (22.0)	13.4 (21.2)
Completeness (%)	39.2 (40.9)	39.3 (41.2)	46.7 (46.0)	45.5 (45.2)
I/σ	6.60 (3.40)	5.68 (3.03)	6.21 (5.61)	6.95 (5.76)
$CC_{1/2}$	99.1 (81.1)	97.7 (79.3)	98.6 (91.2)	98.5 (89.3)
**Crystal**	**Crystal 3**	**Crystal 3**	**Crystal 4**	**Crystal 4**
XDS orientation treatment	Fixed	Refined	Fixed	Refined
Resolution (Å)	10.11 – 0.8 (0.9 – 0.8)	10.11 – 0.8 (0.9 – 0.8)	10.11 – 0.8 (0.9 – 0.8)	10.11 – 0.8 (0.9 – 0.8)
No. observed reflections	1771	1613	1761	1570
No. unique reflections	608	603	682	689
R_{merge} (%)	9.9 (41.0)	9.6 (36.8)	7.7 (33.1)	7.9 (35.6)
R_{meas} (%)	12.4 (47.8)	12.1 (43.5)	9.7 (40.1)	10.3 (43.5)
Completeness (%)	46.3 (45.7)	45.9 (45.7)	51.9 (51.9)	52.4 (52.4)
I/σ	6.71 (2.37)	6.61 (2.60)	5.86 (1.17)	5.55 (1.24)
$CC_{1/2}$	99.0 (90.4)	98.6 (91.4)	99.6 (91.5)	98.8 (89.3)
**Crystal**	**Crystal 5**	**Crystal 5**	**Crystal 6**	**Crystal 6**
XDS orientation treatment	Fixed	Refined	Fixed	Refined
Resolution (Å)	10.11 – 0.8 (0.9 – 0.8)	10.11 – 0.8 (0.9 – 0.8)	10.11 – 0.8 (0.9 – 0.8)	10.11 – 0.8 (0.9 – 0.8)
No. observed reflections	1722	1654	1742	1587
No. unique reflections	640	649	629	673
R_{merge} (%)	9.3 (34.0)	11.2 (35.5)	16.7 (69.4)	12.7 (63.7)
R_{meas} (%)	11.6 (42.1)	14.0 (43.6)	20.7 (87.4)	16.2 (81.0)
Completeness (%)	48.7 (50.5)	49.4 (49.5)	47.9 (50.5)	51.2 (52.7)
I/σ	6.36 (1.83)	4.89 (1.41)	3.82 (1.18)	4.39 (1.10)
$CC_{1/2}$	98.8 (91.0)	98.1 (91.4)	96.8 (16.6)	97.3 (45.7)
**Crystal**	**Crystal 7**	**Crystal 7**	**Crystal 8**	**Crystal 8**
XDS orientation treatment	Fixed	Refined	Fixed	Refined
Resolution (Å)	10.11 – 0.8 (0.9 – 0.8)	10.11 – 0.8 (0.9 – 0.8)	10.11 – 0.8 (0.9 – 0.8)	10.11 – 0.8 (0.9 – 0.8)

No. observed reflections	1752	1715	1773	1513
No. unique reflections	489	505	702	683
R_{merge} (%)	21.1 (107.7)	21.8 (134.1)	68.3 (85.9)	61.5 (76.4)
R_{meas} (%)	24.8 (128.2)	25.4 (164.7)	87.1 (110.5)	78.0 (94.8)
Completeness (%)	37.2 (39.3)	38.4 (39.6)	53.4 (50.5)	52.0 (50.3)
I/σ	3.33 (0.33)	3.03 (0.30)	1.26 (0.49)	1.33 (0.59)
$CC_{1/2}$	97.0 (61.4)	97.7 (-4.0)	16.1 (1.6)	24.9 (40.5)

Statistics of crystallographic data reduction obtained from slowly rotating (0.09 degrees/second) crystals of biotin at 200 kV and 100 K, with and without refinement of orientation performed over 2.5 degree batches in *XDS*. Other parameters able to be refined by *XDS*, such as unit cell dimensions, are held fixed. Data for all crystals was reduced in $P2_12_12_1$ with a, b, c (Å) = 5.13, 10.17, 20.55 and α, β, γ (°) = 90.000, 90.000, 90.000.

Supplementary Table 8. Crystallographic statistics from slowly rotating Zn(II)-methionine crystals

Crystal	Crystal 1	Crystal 1	Crystal 2	Crystal 2
XDS orientation treatment	Fixed	Refined	Fixed	Refined
Resolution (Å)	8.52 – 0.8 (0.9 – 0.8)	8.52 – 0.8 (0.9 – 0.8)	8.52 – 0.8 (0.9 – 0.8)	8.52 – 0.8 (0.9 – 0.8)
No. observed reflections	1085	1014	958	948
No. unique reflections	436	438	370	373
R_{merge} (%)	30.3 (84.7)	24.4 (67.7)	20.9 (29.5)	18.7 (23.7)
R_{meas} (%)	38.5 (106.7)	31.4 (86.0)	26.2 (38.4)	23.9 (31.2)
Completeness (%)	28.6 (27.4)	28.7 (26.9)	24.3 (22.0)	24.5 (22.7)
I/σ	2.19 (1.35)	2.74 (1.81)	4.77 (3.10)	5.01 (3.53)
$CC_{1/2}$	89.3 (-6.2)	91.2 (22.4)	96.5 (90.9)	91.1 (91.4)
**Crystal**	**Crystal 3**	**Crystal 3**	**Crystal 4**	**Crystal 4**
XDS orientation treatment	Fixed	Refined	Fixed	Refined
Resolution (Å)	8.52 – 0.8 (0.9 – 0.8)	8.52 – 0.8 (0.9 – 0.8)	8.52 – 0.8 (0.9 – 0.8)	8.52 – 0.8 (0.9 – 0.8)
No. observed reflections	1036	1003	1018	972
No. unique reflections	347	320	415	436
R_{merge} (%)	37.2 (72.2)	34.8 (75.7)	14.4 (28.7)	15.0 (27.8)
R_{meas} (%)	45.2 (93.2)	42.1 (95.5)	18.1 (35.8)	19.1 (35.1)
Completeness (%)	22.8 (20.9)	21.0 (18.8)	27.2 (26.5)	28.6 (26.7)
I/σ	2.96 (2.25)	4.08 (3.17)	4.14 (3.07)	3.30 (2.47)
$CC_{1/2}$	66.0 (-16.1)	70.3 (-8.4)	98.0 (96.0)	98.9 (90.4)
**Crystal**	**Crystal 5**	**Crystal 5**		
XDS orientation treatment	Fixed	Refined		
Resolution (Å)	8.52 – 0.8 (0.9 – 0.8)	8.52 – 0.8 (0.9 – 0.8)		
No. observed reflections	1095	976		
No. unique reflections	478	464		
R_{merge} (%)	27.3 (25.0)	20.3 (22.0)		
R_{meas} (%)	36.1 (33.0)	27.1 (29.0)		
Completeness (%)	31.4 (28.3)	30.4 (27.6)		
I/σ	2.29 (2.81)	2.50 (3.01)		
$CC_{1/2}$	85.3 (47.3)	94.2 (60.4)		

Statistics of crystallographic data reduction obtained from slowly rotating (0.09 degrees/second) crystals of Zn(II)-methionine at 200 kV and 100 K, with and without refinement of orientation performed over 2.5 degree batches in *XDS*. Other parameters able to be refined by *XDS*, such

as unit cell dimensions, are held fixed. Data for all crystals was reduced in $P2_1$ with a, b, c (Å) = 9.35, 4.97, 14.92 and α, β, γ (°) = 90.000, 106.474, 90.000.

Supplementary Table 9. Crystallographic statistics from slowly rotating Co(II)-porphyrin crystals

**Crystal**	**Crystal 1**	**Crystal 1**	**Crystal 2**	**Crystal 2**
XDS orientation treatment	Fixed	Refined	Fixed	Refined
Resolution (Å)	12.57 – 0.8 (0.9 – 0.8)	12.57 – 0.8 (0.9 – 0.8)	12.57 – 0.8 (0.9 – 0.8)	12.57 – 0.8 (0.9 – 0.8)
No. observed reflections	2260	2203	2296	2063
No. unique reflections	1225	1294	1236	1239
R_{merge} (%)	8.6 (22.5)	9.1 (24.6)	6.1 (14.6)	6.0 (10.9)
R_{meas} (%)	12.1 (31.9)	12.9 (34.9)	8.6 (20.7)	8.5 (15.5)
Completeness (%)	20.1 (21.0)	21.3 (21.2)	20.3 (20.9)	20.4 (21.6)
I/σ	5.39 (2.40)	4.85 (2.06)	7.69 (4.19)	7.00 (5.65)
$CC_{1/2}$	98.7 (81.7)	98.6 (81.4)	99.4 (86.4)	99.5 (92.2)
**Crystal**	**Crystal 3**	**Crystal 3**	**Crystal 4**	**Crystal 4**
XDS orientation treatment	Fixed	Refined	Fixed	Refined
Resolution (Å)	12.57 – 0.8 (0.9 – 0.8)	12.57 – 0.8 (0.9 – 0.8)	12.57 – 0.8 (0.9 – 0.8)	12.57 – 0.8 (0.9 – 0.8)
No. observed reflections	2269	2229	2317	2302
No. unique reflections	1228	1224	1245	1277
R_{merge} (%)	5.1 (29.1)	5.9 (27.5)	8.8 (10.7)	8.8 (11.5)
R_{meas} (%)	7.2 (41.2)	8.3 (38.9)	12.4 (15.2)	12.4 (16.3)
Completeness (%)	20.2 (21.5)	20.1 (21.4)	20.5 (20.8)	21.0 (21.6)
I/σ	8.44 (1.61)	7.86 (1.70)	6.91 (5.81)	6.11 (4.69)
$CC_{1/2}$	99.6 (80.3)	99.3 (80.9)	98.2 (93.7)	98.6 (93.0)
**Crystal**	**Crystal 5**	**Crystal 5**		
XDS orientation treatment	Fixed	Refined		
Resolution (Å)	12.57 – 0.8 (0.9 – 0.8)	12.57 – 0.8 (0.9 – 0.8)		
No. observed reflections	2175	2088		
No. unique reflections	1176	1243		
R_{merge} (%)	4.6 (9.8)	6.3 (10.7)		
R_{meas} (%)	6.5 (13.8)	8.8 (15.1)		
Completeness (%)	19.3 (19.7)	20.4 (19.7)		
I/σ	10.54 (6.08)	7.52 (5.83)		
$CC_{1/2}$	99.6 (90.5)	99.2 (89.7)		

Statistics of crystallographic data reduction obtained from slowly rotating (0.09 degrees/second) crystals of Co(II) tetraphenyl porphyrin at 200 kV and 293 K, with and without refinement of orientation performed over 2.5 degree batches in *XDS*. Other parameters able to be refined by

XDS, such as unit cell dimensions, are held fixed. Data for all crystals was reduced in $P\bar{1}$ with a, b, c (Å) = 9.60, 13.27, 13.28 and α, β, γ (°) = 87.353, 108.223, 112.00.

Supplementary Table 10. Crystallographic statistics from fast tilt series (multiple sweeps) on biotin crystals at 0.01 $e^-/Å^2s$

**Crystal**	**Crystal 1**	**Crystal 1**	**Crystal 1**	**Crystal 1**
**Sweep**	**1**	**2**	**3**	**4**
Range of total fluence ($e^-/Å^2$)	0 – 0.9	0.9 – 1.8	1.8 – 2.7	2.7 – 3.6
Crystal system	Orthorhombic	Orthorhombic	Orthorhombic	Orthorhombic
Space group	$P2_12_12_1$	$P2_12_12_1$	$P2_12_12_1$	$P2_12_12_1$
Unit cell dimensions:				
a, b, c (Å)	5.14, 10.02, 20.47	5.15, 10.08, 20.51	5.16, 10.15, 20.54	5.16, 10.20, 20.58
α, β, γ (°)	90.000, 90.000, 90.000	90.000, 90.000, 90.000	90.000, 90.000, 90.000	90.000, 90.000, 90.000
Resolution (Å)	10.11 – 0.8 (0.9 – 0.8)	10.11 – 0.8 (0.9 – 0.8)	10.11 – 0.8 (0.9 – 0.8)	10.11 – 0.8 (0.9 – 0.8)
No. observed reflections	3983	4070	4067	4051
No. unique reflections	1108	1125	1142	1145
R_{merge} (%)	12.0 (24.6)	15.1 (31.4)	16.8 (43.6)	16.9 (55.6)
R_{meas} (%)	14.1 (28.5)	17.6 (36.4)	19.8 (50.9)	20.2 (65.4)
Completeness (%)	85.8 (85.1)	86.3 (86.1)	86.5 (85.4)	85.9 (83.5)
I/σ	7.17 (4.90)	5.24 (3.71)	4.35 (2.80)	3.73 (2.46)
$CC_{1/2}$	98.4 (78.9)	98.1 (70.2)	98.0 (34.4_	97.7 (28.9)
Refinement				
R1 (%)	17.70	17.77	20.11	24.20
wR2 (%)	45.54	46.20	51.16	58.75
Goodness of fit	1.652	1.443	1.443	1.523
**Crystal**	**Crystal 2**	**Crystal 2**	**Crystal 2**	**Crystal 2**
**Sweep**	**1**	**2**	**3**	**4**
Range of total fluence ($e^-/Å^2$)	0 – 0.9	0.9 – 1.8	1.8 – 2.7	2.7 – 3.6
Crystal system	Orthorhombic	Orthorhombic	Orthorhombic	Orthorhombic
Space group	$P2_12_12_1$	$P2_12_12_1$	$P2_12_12_1$	$P2_12_12_1$
Unit cell dimensions:				
a, b, c (Å)	5.13, 10.02, 20.47	5.14, 10.05, 20.51	5.15, 10.13, 20.55	5.16, 10.22, 20.56
α, β, γ (°)	90.000, 90.000, 90.000	90.000, 90.000, 90.000	90.000, 90.000, 90.000	90.000, 90.000, 90.000
Resolution (Å)	10.11 – 0.8 (0.9 – 0.8)	10.11 – 0.8 (0.9 – 0.8)	10.11 – 0.8 (0.9 – 0.8)	10.11 – 0.8 (0.9 – 0.8)
No. observed reflections	4005	3994	3870	3964
No. unique reflections	1088	1093	1123	1140
R_{merge} (%)	13.5 (27.7)	13.7 (30.9)	15.9 (35.8)	21.3 (47.1)
R_{meas} (%)	15.9 (32.3)	16.0 (36.0)	18.9 (42.2)	25.5 (56.0)

Completeness (%)	84.5 (83.8)	84.1 (82.0)	85.3 (84.6)	85.6 (83.9)
I/σ	6.86 (4.76)	6.04 (4.28)	4.77 (3.59)	3.27 (3.34)
$CC_{1/2}$	98.6 (74.4)	98.8 (58.4)	98.6 (38.9)	98.0 (13.8)
Refinement				
R1 (%)	16.70	17.02	22.56	N/A
wR2 (%)	43.84	45.06	55.07	N/A
Goodness of fit	1.526	1.487	1.635	N/A
**Crystal**	**Crystal 3**	**Crystal 3**	**Crystal 3**	**Crystal 3**
**Sweep**	**1**	**2**	**3**	**4**
Range of total fluence ($e^-/Å^2$)	0 – 0.9	0.9 – 1.8	1.8 – 2.7	2.7 – 3.6
Crystal system	Orthorhombic	Orthorhombic	Orthorhombic	Orthorhombic
Space group	$P2_12_12_1$	$P2_12_12_1$	$P2_12_12_1$	$P2_12_12_1$
Unit cell dimensions:				
a, b, c (Å)	5.13, 10.09, 20.42	5.14, 10.12, 20.43	5.16, 10.17, 20.51	5.17, 10.21, 20.55
α, β, γ (°)	90.000, 90.000, 90.000	90.000, 90.000, 90.000	90.000, 90.000, 90.000	90.000, 90.000, 90.000
Resolution (Å)	10.11 – 0.8 (0.9 – 0.8)	10.11 – 0.8 (0.9 – 0.8)	10.11 – 0.8 (0.9 – 0.8)	10.11 – 0.8 (0.9 – 0.8)
No. observed reflections	3979	3990	3982	4009
No. unique reflections	1100	1116	1135	1149
R_{merge} (%)	13.2 (28.3)	14.5 (36.3)	17.4 (52.5)	18.5 (83.8)
R_{meas} (%)	15.5 (32.9)	17.1 (42.4)	20.4 (61.1)	21.9 (98.3)
Completeness (%)	85.2 (85.9)	85.6 (86.0)	86.1 (86.5)	86.2 (86.4)
I/σ	6.55 (4.27)	5.42 (3.26)	4.21 (2.29)	3.13 (1.49)
$CC_{1/2}$	98.2 (78.4)	97.5 (72.4)	96.5 (38.3)	98.4 (28.4)
Refinement				
R1 (%)	18.16	18.54	18.51	20.87
wR2 (%)	47.97	49.84	50.48	57.35
Goodness of fit	1.678	1.595	1.398	1.364
**Crystal**	**Crystal 4**	**Crystal 4**	**Crystal 4**	**Crystal 4**
**Sweep**	**1**	**2**	**3**	**4**
Range of total fluence ($e^-/Å^2$)	0 – 0.9	0.9 – 1.8	1.8 – 2.7	2.7 – 3.6
Crystal system	Orthorhombic	Orthorhombic	Orthorhombic	Orthorhombic
Space group	$P2_12_12_1$	$P2_12_12_1$	$P2_12_12_1$	$P2_12_12_1$
Unit cell dimensions:				
a, b, c (Å)	5.11, 10.04, 20.56	5.13, 10.09, 20.58	5.14, 10.17, 20.60	5.14, 10.27, 20.64
α, β, γ (°)	90.000, 90.000, 90.000	90.000, 90.000, 90.000	90.000, 90.000, 90.000	90.000, 90.000, 90.000
Resolution (Å)	10.11 – 0.8 (0.9 – 0.8)	10.11 – 0.8 (0.9 – 0.8)	10.11 – 0.8 (0.9 – 0.8)	10.11 – 0.8 (0.9 – 0.8)

No. observed reflections	3962	4015	4014	3951
No. unique reflections	1177	1184	1208	1218
R_{merge} (%)	18.2 (27.8)	14.1 (30.1)	14.3 (34.0)	22.1 (46.7)
R_{meas} (%)	21.5 (32.8)	16.7 (35.6)	17.2 (40.6)	26.7 (55.9)
Completeness (%)	90.9 (88.7)	90.7 (88.9)	91.7 (90.8)	91.1 (88.7)
I/σ	5.20 (4.44)	5.86 (4.58)	5.36 (4.61)	3.17 (3.49)
$CC_{1/2}$	96.1 (64.6)	98.0 (49.6)	98.1 (25.0)	97.7 (27.8)
Refinement				
R1 (%)	21.89	23.19	29.40	N/A
wR2 (%)	52.71	55.39	63.05	N/A
Goodness of fit	1.661	1.839	2.039	N/A
**Crystal**	**Crystal 5**	**Crystal 5**	**Crystal 5**	**Crystal 5**
**Sweep**	**1**	**2**	**3**	**4**
Range of total fluence ($e^-/Å^2$)	0 – 0.9	0.9 – 1.8	1.8 – 2.7	2.7 – 3.6
Crystal system	Orthorhombic	Orthorhombic	Orthorhombic	Orthorhombic
Space group	$P2_12_12_1$	$P2_12_12_1$	$P2_12_12_1$	$P2_12_12_1$
Unit cell dimensions:				
a, b, c (Å)	5.11, 10.08, 20.42	5.12, 10.13, 20.46	5.13, 10.23, 20.50	5.15, 10.27, 20.54
α, β, γ (°)	90.000, 90.000, 90.000	90.000, 90.000, 90.000	90.000, 90.000, 90.000	90.000, 90.000, 90.000
Resolution (Å)	10.11 – 0.8 (0.9 – 0.8)	10.11 – 0.8 (0.9 – 0.8)	10.11 – 0.8 (0.9 – 0.8)	10.11 – 0.8 (0.9 – 0.8)
No. observed reflections	3951	3997	4023	3985
No. unique reflections	1077	1075	1085	1089
R_{merge} (%)	12.9 (26.8)	12.4 (34.1)	15.5 (50.4)	16.2 (68.7)
R_{meas} (%)	15.0 (31.3)	14.5 (40.3)	18.1 (58.9)	19.2 (80.3)
Completeness (%)	83.7 (85.0)	82.7 (84.6)	82.4 (83.6)	81.5 (81.4)
I/σ	6.90 (4.28)	6.62 (3.52)	4.58 (2.43)	3.96 (1.86)
$CC_{1/2}$	98.8 (87.2)	98.7 (76.1)	98.6 (70.3)	98.6 (19.6)
Refinement				
R1 (%)	16.82	17.55	19.90	22.81
wR2 (%)	43.06	44.44	51.64	57.07
Goodness of fit	1.512	1.502	1.473	1.525
**Crystal**	**Crystal 6**	**Crystal 6**	**Crystal 6**	**Crystal 6**
**Sweep**	**1**	**2**	**3**	**4**
Range of total fluence ($e^-/Å^2$)	0 – 0.9	0.9 – 1.8	1.8 – 2.7	2.7 – 3.6
Crystal system	Orthorhombic	Orthorhombic	Orthorhombic	Orthorhombic
Space group	$P2_12_12_1$	$P2_12_12_1$	$P2_12_12_1$	$P2_12_12_1$
Unit cell dimensions:				
a, b, c (Å)	5.09, 10.08, 20.65	5.11, 10.11, 20.68	5.13, 10.15, 20.72	5.15, 10.21, 20.72

α, β, γ (°)	90.000, 90.000, 90.000	90.000, 90.000, 90.000	90.000, 90.000, 90.000	90.000, 90.000, 90.000
Resolution (Å)	10.11 – 0.8 (0.9 – 0.8)	10.11 – 0.8 (0.9 – 0.8)	10.11 – 0.8 (0.9 – 0.8)	10.11 – 0.8 (0.9 – 0.8)
No. observed reflections	3904	4004	4037	4049
No. unique reflections	1082	1099	1108	1134
R_{merge} (%)	14.6 (35.4)	15.0 (43.4)	14.1 (54.5)	14.2 (76.7)
R_{meas} (%)	17.2 (41.3)	17.7 (50.9)	16.7 (63.3)	16.9 (89.7)
Completeness (%)	83.3 (83.5)	84.0 (84.1)	83.8 (84.5)	85.1 (84.7)
I/σ	5.49 (3.17)	4.93 (2.59)	4.79 (2.18)	4.10 (1.75)
$CC_{1/2}$	98.4 (79.9)	98.4 (58.8)	98.9 (42.6)	99.2 (10.7)
Refinement				
R1 (%)	16.79	15.75	17.50	20.91
wR2 (%)	44.46	43.79	47.50	53.68
Goodness of fit	1.408	1.296	1.364	1.414

Statistics of crystallographic data reduction and structure refinement obtained from biotin crystals rotated at 1 degree/second while illuminated at a rate of 0.01 $e^-/Å^2s$, at 200 kV and 100 K, with an oscillation range of 1 degree per frame. Multiple sweeps of data (0.9 $e^-/Å^2$ total fluence delivered during each) were collected on each crystal, where data from 4 sweeps could typically be processed readily.

Supplementary Table 11. Crystallographic statistics from fast tilt series (multiple sweeps) on biotin crystals at 0.03 $e^-/Å^2s$

**Crystal**	**Crystal 1**	**Crystal 1**	**Crystal 2**	**Crystal 2**
**Sweep**	**1**	**2**	**1**	**2**
Range of total fluence ($e^-/Å^2$)	0 – 2.7	2.7 – 5.4	0 – 2.7	2.7 – 5.4
Crystal system	Orthorhombic	Orthorhombic	Orthorhombic	Orthorhombic
Space group	$P2_12_12_1$	$P2_12_12_1$	$P2_12_12_1$	$P2_12_12_1$
Unit cell dimensions:				
a, b, c (Å)	5.13, 10.19, 20.48	5.15, 10.38, 20.59	5.12, 10.13, 19.97	5.16, 10.24, 20.19
α, β, γ (°)	90.000, 90.000, 90.000	90.000, 90.000, 90.000	90.000, 90.000, 90.000	90.000, 90.000, 90.000
Resolution (Å)	10.11 – 0.8 (0.9 – 0.8)	10.11 – 0.8 (0.9 – 0.8)	10.11 – 0.8 (0.9 – 0.8)	10.11 – 0.8 (0.9 – 0.8)
No. observed reflections	3744	3345	3928	3920
No. unique reflections	1189	1107	736	682
R_{merge} (%)	14.4 (27.2)	13.1 (33.0)	19.7 (54.4)	28.8 (221.7)
R_{meas} (%)	17.4 (32.2)	15.7 (40.6)	21.8 (60.3)	31.7 (243.7)
Completeness (%)	90.8 (91.1)	81.9 (82.5)	58.1 (60.2)	52.5 (54.7)
I/σ	4.84 (3.05)	4.33 (3.11)	5.28 (2.93)	3.13 (0.74)
$CC_{1/2}$	97.3 (92.4)	97.7 (81.8)	97.9 (76.1)	98.6 (9.9)
Refinement				
R1 (%)	N/A	N/A	N/A	N/A
wR2 (%)	N/A	N/A	N/A	N/A
Goodness of fit	N/A	N/A	N/A	N/A
**Crystal**	**Crystal 3**	**Crystal 3**	**Crystal 4**	**Crystal 4**
**Sweep**	**1**	**2**	**1**	**2**
Range of total fluence ($e^-/Å^2$)	0 – 2.7	2.7 – 5.4	0 – 2.7	2.7 – 5.4
Crystal system	Orthorhombic	Orthorhombic	Orthorhombic	Orthorhombic
Space group	$P2_12_12_1$	$P2_12_12_1$	$P2_12_12_1$	$P2_12_12_1$
Unit cell dimensions:				
a, b, c (Å)	5.10, 10.09, 20.60	5.12, 10.19, 20.76	5.09, 10.11, 20.70	5.14, 10.25, 20.85
α, β, γ (°)	90.000, 90.000, 90.000	90.000, 90.000, 90.000	90.000, 90.000, 90.000	90.000, 90.000, 90.000
Resolution (Å)	10.11 – 0.8 (0.9 – 0.8)	10.11 – 0.8 (0.9 – 0.8)	10.11 – 0.8 (0.9 – 0.8)	10.11 – 0.8 (0.9 – 0.8)
No. observed reflections	4058	3953	4010	4203
No. unique reflections	1039	1061	1120	961
R_{merge} (%)	15.4 (35.4)	20.9 (182.8)	22.5 (60.3)	31.3 (479.7)
R_{meas} (%)	17.9 (40.9)	24.5 (213.8)	25.4 (68.0)	35.5 (543.4)

Completeness (%)	79.9 (81.9)	80.0 (81.7)	70.2 (72.0)	71.5 (73.6)
I/σ	5.91 (3.46)	2.88 (0.59)	4.69 (2.49)	2.06 (0.30)
$CC_{1/2}$	97.8 (88.6)	96.9 (40.0)	96.9 (68.2)	97.9 (4.8)
Refinement				
R1 (%)	16.82	22.87	18.74	N/A
wR2 (%)	46.38	61.04	51.57	N/A
Goodness of fit	1.521	1.427	1.519	N/A
**Crystal**	**Crystal 5**	**Crystal 5**		
**Sweep**	**1**	**2**		
Range of total fluence ($e^-/Å^2$)	0 – 0.9	0.9 – 1.8		
Crystal system	Orthorhombic	Orthorhombic		
Space group	$P2_12_12_1$	$P2_12_12_1$		
Unit cell dimensions:				
a, b, c (Å)	5.11, 10.11, 20.61	5.09, 10.14, 20.62		
α, β, γ (°)	90.000, 90.000, 90.000	90.000, 90.000, 90.000		
Resolution (Å)	10.11 – 0.8 (0.9 – 0.8)	10.11 – 0.8 (0.9 – 0.8)		
No. observed reflections	3967	3982		
No. unique reflections	1061	1144		
R_{merge} (%)	27.4 (44.7)	37.9 (72.9)		
R_{meas} (%)	31.7 (51.2)	44.6 (87.8)		
Completeness (%)	81.2 (82.2)	87.5 (87.2)		
I/σ	4.18 (2.69)	2.78 (1.84)		
$CC_{1/2}$	95.4 (73.3)	95.8 (67.1)		
Refinement				
R1 (%)	20.30			
wR2 (%)	53.11			
Goodness of fit	1.462			

Statistics of crystallographic data reduction and structure refinement obtained from biotin crystals rotated at 1 degree/second while illuminated at a rate of 0.03 $e^-/Å^2s$, at 200 kV and 100 K, with an oscillation range of 1 degree per frame. Multiple sweeps of data (2.7 $e^-/Å^2$ total fluence delivered during each) were collected on each crystal, where data from 2 sweeps could typically be processed readily, and the third was generally too compromised by radiolytic damage-induced decay to be indexed. Not every indexable dataset readily yielded a structure solution by direct methods; for those that did not, refinement statistics are omitted (N/A).

Supplementary Table 12. Crystallographic statistics from fast tilt series (multiple sweeps) on Zn(II)-methionine crystals at 0.01 $e^{-}/Å^{2}s$

**Crystal**	**Crystal 1**	**Crystal 1**	**Crystal 2**	**Crystal 2**
**Sweep**	**1**	**2**	**1**	**2**
Range of total fluence ($e^{-}/Å^{2}$)	0 – 1.0	1.0 – 2.0	0 – 1.0	1.0 – 2.0
Crystal system	Monoclinic	Monoclinic	Monoclinic	Monoclinic
Space group	$P2_1$	$P2_1$	$P2_1$	$P2_1$
Unit cell dimensions:				
a, b, c (Å)	9.21, 5.01, 15.21	9.27, 5.01, 15.53	9.21, 5.01, 15.26	9.30, 5.01, 15.49
α, β, γ (°)	90.000, 107.625, 90.000	90.000, 107.279, 90.000	90.000, 107.355, 90.000	90.000, 107.412, 90.000
Resolution (Å)	8.52 – 0.8 (0.9 – 0.8)	8.52 – 0.8 (0.9 – 0.8)	8.52 – 0.8 (0.9 – 0.8)	8.52 – 0.8 (0.9 – 0.8)
No. observed reflections	2971	2871	2788	2849
No. unique reflections	936	951	1055	1089
R_{merge} (%)	13.0 (22.3)	11.9 (40.0)	12.3 (21.2)	11.2 (36.4)
R_{meas} (%)	15.7 (26.8)	14.3 (48.2)	15.4 (26.2)	14.1 (45.7)
Completeness (%)	61.4 (59.9)	60.2 (59.8)	68.6 (68.2)	68.8 (68.4)
I/σ	6.15 (4.04)	5.39 (2.44)	6.27 (4.11)	4.88 (2.22)
$CC_{1/2}$	97.6 (91.0)	98.8 (82.5)	97.1 (88.0)	98.8 (74.9)
**Crystal**	**Crystal 3**	**Crystal 3**	**Crystal 4**	**Crystal 4**
**Sweep**	**1**	**2**	**1**	**2**
Range of total fluence ($e^{-}/Å^{2}$)	0 – 1.0	1.0 – 2.0	0 – 1.0	1.0 – 2.0
Crystal system	Monoclinic	Monoclinic	Monoclinic	Monoclinic
Space group	$P2_1$	$P2_1$	$P2_1$	$P2_1$
Unit cell dimensions:				
a, b, c (Å)	9.26, 4.98, 15.17	9.32, 4.98, 15.38	9.24, 5.01, 15.17	9.33, 5.01, 15.44
α, β, γ (°)	90.000, 107.512, 90.000	90.000, 107.351, 90.000	90.000, 107.497, 90.000	90.000, 107.199, 90.000
Resolution (Å)	8.52 – 0.8 (0.9 – 0.8)	8.52 – 0.8 (0.9 – 0.8)	8.52 – 0.8 (0.9 – 0.8)	8.52 – 0.8 (0.9 – 0.8)
No. observed reflections	2815	2866	2783	2835
No. unique reflections	1041	1070	994	1033
R_{merge} (%)	12.6 (21.5)	13.0 (42.0)	12.3 (21.5)	16.5 (59.0)
R_{meas} (%)	15.8 (26.4)	16.3 (52.1)	15.1 (26.5)	20.6 (73.3)
Completeness (%)	68.4 (68.0)	68.3 (66.5)	65.0 (64.9)	65.1 (63.6)
I/σ	5.81 (3.86)	4.72 (2.30)	5.85 (3.72)	2.85 (1.57)
$CC_{1/2}$	97.6 (86.4)	98.5 (56.5)	98.0 (89.1)	98.3 (23.1)

Statistics of crystallographic data reduction obtained from Zn(II)-methionine crystals rotated at 1 degree/second while illuminated at a rate of 0.01 $e^-/Å^2s$, at 200 kV and 100 K, with an oscillation range of 1 degree/frame. Multiple sweeps of data (1.0 $e^-/Å^2$ total fluence delivered during each) were collected on each crystal. Analysis of refinement statistics is omitted, as 100° tilt series do not reliably afford sufficiently high completeness to yield *ab initio* phasing solutions with direct methods from single crystals of Zn(II)-methionine.

Supplementary Table 13. Crystallographic statistics from fast tilt series (multiple sweeps) on Zn(II)-histidine crystals at 0.01 $e^{-}/Å^{2}s$

**Crystal**	**Crystal 1**	**Crystal 1**	**Crystal 1**	**Crystal 1**
**Sweep**	**1**	**2**	**3**	**4**
Range of total fluence ($e^{-}/Å^{2}$)	0 – 0.9	0.9 – 1.8	1.8 – 2.7	2.7 – 3.6
Crystal system	Tetragonal	Tetragonal	Tetragonal	N/A
Space group	$P4_32_12$	$P4_32_12$	$P4_32_12$	N/A
Unit cell dimensions:				
a, b, c (Å)	7.06, 7.06, 28.89	7.05, 7.05, 28.96	7.04, 7.04, 28.99	N/A
α, β, γ (°)	90.000, 90.000, 90.000	90.000, 90.000, 90.000	90.000, 90.000, 90.000	N/A
Resolution (Å)	7.218 – 0.8 (0.9 – 0.8)	7.218 – 0.8 (0.9 – 0.8)	7.218 – 0.8 (0.9 – 0.8)	N/A
No. observed reflections	5446	5447	5597	N/A
No. unique reflections	646	665	668	N/A
R_{merge} (%)	20.0 (55.7)	19.8 (70.5)	21.1 (82.2)	N/A
R_{meas} (%)	21.4 (59.2)	21.3 (74.9)	22.6 (87.5)	N/A
Completeness (%)	68.1 (69.3)	70.0 (69.4)	70.2 (69.0)	N/A
I/σ	6.62 (3.27)	6.03 (2.68)	5.25 (2.52)	N/A
$CC_{1/2}$	99.0 (64.6)	99.3 (28.8)	99.3 (6.5)	N/A
Refinement				
R1 (%)	20.17	19.89	21.45	N/A
wR2 (%)	51.12	49.63	52.62	N/A
Goodness of fit	1.832	1.597	1.579	N/A
**Crystal**	**Crystal 2**	**Crystal 2**	**Crystal 2**	**Crystal 2**
**Sweep**	**1**	**2**	**3**	**4**
Range of total fluence ($e^{-}/Å^{2}$)	0 – 0.9	0.9 – 1.8	1.8 – 2.7	2.7 – 3.6
Crystal system	Tetragonal	Tetragonal	Tetragonal	Tetragonal
Space group	$P4_32_12$	$P4_32_12$	$P4_32_12$	$P4_32_12$
Unit cell dimensions:				
a, b, c (Å)	7.08, 7.08, 29.09	7.07, 7.07, 29.19	7.05, 7.05, 29.22	7.05, 7.05, 29.23
α, β, γ (°)	90.000, 90.000, 90.000	90.000, 90.000, 90.000	90.000, 90.000, 90.000	90.000, 90.000, 90.000
Resolution (Å)	7.218 – 0.8 (0.9 – 0.8)	7.218 – 0.8 (0.9 – 0.8)	7.218 – 0.8 (0.9 – 0.8)	7.218 – 0.8 (0.9 – 0.8)
No. observed reflections	5251	5320	5523	5195
No. unique reflections	878	873	873	872
R_{merge} (%)	18.8 (43.4)	18.6 (54.2)	16.4 (64.1)	27.6 (82.9)
R_{meas} (%)	20.5 (40.9)	20.3 (58.4)	17.9 (69.1)	30.2 (89.5)

Completeness (%)	91.3 (91.2)	90.4 (90.9)	90.7 (88.6)	90.7 (91.2)
I/σ	6.04 (3.17)	5.69 (2.69)	6.31 (2.36)	3.26 (1.85)
$CC_{1/2}$	98.1 (64.8)	98.7 (48.9)	99.2 (37.8)	96.7 (17.8)
Refinement				
R1 (%)	19.87	19.89	19.46	N/A
wR2 (%)	51.66	49.85	50.81	N/A
Goodness of fit	1.785	1.629	1.656	N/A
**Crystal**	**Crystal 3**	**Crystal 3**	**Crystal 3**	**Crystal 3**
**Sweep**	**1**	**2**	**3**	**4**
Range of total fluence (e^-/Å^2)	0 – 0.9	0.9 – 1.8	1.8 – 2.7	2.7 – 3.6
Crystal system	Tetragonal	Tetragonal	Tetragonal	Tetragonal
Space group	$P4_32_12$	$P4_32_12$	$P4_32_12$	$P4_32_12$
Unit cell dimensions:				
a, b, c (Å)	7.06, 7.06, 29.03	7.06, 7.06, 29.12	7.04, 7.04, 29.12	7.04, 7.04, 29.12
α, β, γ (°)	90.000, 90.000, 90.000	90.000, 90.000, 90.000	90.000, 90.000, 90.000	90.000, 90.000, 90.000
Resolution (Å)	7.218 – 0.8 (0.9 – 0.8)	7.218 – 0.8 (0.9 – 0.8)	7.218 – 0.8 (0.9 – 0.8)	7.218 – 0.8 (0.9 – 0.8)
No. observed reflections	5508	5489	5612	5545
No. unique reflections	946	947	943	945
R_{merge} (%)	23.6 (57.5)	27.6 (67.7)	29.0 (72.9)	33.3 (85.1)
R_{meas} (%)	26.0 (62.9)	30.5 (74.2)	31.9 (79.9)	36.7 (93.2)
Completeness (%)	99.0 (98.5)	99.1 (99.3)	99.0 (99.3)	99.2 (99.6)
I/σ	5.07 (2.72)	4.10 (2.25)	4.02 (2.19)	3.68 (1.85)
$CC_{1/2}$	98.8 (27.9)	98.2 (21.0)	98.6 (1.1)	99.0 (9.5)
Refinement				
R1 (%)	23.21	22.88	25.41	N/A
wR2 (%)	52.66	53.51	56.14	N/A
Goodness of fit	1.620	1.478	1.500	N/A
**Crystal**	**Crystal 4**	**Crystal 4**	**Crystal 4**	**Crystal 4**
**Sweep**	**1**	**2**	**3**	**4**
Range of total fluence (e^-/Å^2)	0 – 0.9	0.9 – 1.8	1.8 – 2.7	2.7 – 3.6
Crystal system	Tetragonal	Tetragonal	Tetragonal	Tetragonal
Space group	$P4_32_12$	$P4_32_12$	$P4_32_12$	$P4_32_12$
Unit cell dimensions:				
a, b, c (Å)	7.07, 7.07, 29.03	7.06, 7.06, 29.09	7.05, 7.05, 29.11	7.05, 7.05, 29.10
α, β, γ (°)	90.000, 90.000, 90.000	90.000, 90.000, 90.000	90.000, 90.000, 90.000	90.000, 90.000, 90.000
Resolution (Å)	7.218 – 0.8 (0.9 – 0.8)	7.218 – 0.8 (0.9 – 0.8)	7.218 – 0.8 (0.9 – 0.8)	7.218 – 0.8 (0.9 – 0.8)

No. observed reflections	5370	5013	5485	5060
No. unique reflections	774	770	773	773
R_{merge} (%)	24.0 (55.2)	24.6 (56.1)	22.0 (67.1)	33.9 (86.0)
R_{meas} (%)	24.6 (48.8)	26.6 (60.3)	23.7 (71.6)	36.7 (92.3)
Completeness (%)	80.8 (79.3)	80.5 (79.3)	81.0 (81.4)	81.1 (81.8)
I/σ	5.68 (3.37)	4.99 (2.62)	5.52 (2.51)	2.98 (1.78)
$CC_{1/2}$	97.7 (59.2)	97.7 (38.4)	98.7 (3.7)	95.8 (22.2)
Refinement				
R1 (%)	18.66	21.13	20.08	N/A
wR2 (%)	47.99	52.32	49.75	N/A
Goodness of fit	1.618	1.640	1.571	N/A

Statistics of crystallographic data reduction and structure refinement obtained from Zn(II)-histidine crystals rotated at 1 degree/second while illuminated at a rate of 0.01 $e^-/Å^2s$, at 200 kV and room temperature, with an oscillation range of 1 degree per frame. Multiple sweeps of data (0.8 $e^-/Å^2$ total fluence delivered during each) were collected on each crystal, where data from 4 sweeps could typically be processed readily.

Supplementary Table 14. Crystallographic statistics from fast tilt series (multiple sweeps) on Zn(II)-histidine crystals at 0.03 $e^-/Å^2s$

**Crystal**	**Crystal 1**	**Crystal 1**	**Crystal 2**	**Crystal 2**
**Sweep**	**1**	**2**	**1**	**2**
Range of total fluence ($e^-/Å^2$)	0 – 2.7	2.7 – 5.4	0 – 2.7	2.7 – 5.4
Crystal system	Tetragonal	Tetragonal	Tetragonal	Tetragonal
Space group	$P4_32_12$	$P4_32_12$	$P4_32_12$	$P4_32_12$
Unit cell dimensions:				
a, b, c (Å)	7.07, 7.07, 29.00	7.05, 7.05, 29.02	7.06, 7.06, 28.93	7.05, 7.05, 28.97
α, β, γ (°)	90.000, 90.000, 90.000	90.000, 90.000, 90.000	90.000, 90.000, 90.000	90.000, 90.000, 90.000
Resolution (Å)	7.218 – 0.8 (0.9 – 0.8)	7.218 – 0.8 (0.9 – 0.8)	7.218 – 0.8 (0.9 – 0.8)	7.218 – 0.8 (0.9 – 0.8)
No. observed reflections	5423	5477	4650	4697
No. unique reflections	906	904	684	682
R_{merge} (%)	15.5 (43.5)	21.4 (181.0)	22.3 (104.8)	34.3 (334.8)
R_{meas} (%)	17.0 (47.3)	23.6 (197.2)	24.3 (113.4)	37.6 (361.1)
Completeness (%)	94.6 (94.1)	94.9 (94.1)	71.8 (71.7)	71.6 (71.9)
I/σ	7.27 (3.41)	3.59 (0.76)	5.65 (2.00)	2.91 (0.49)
$CC_{1/2}$	99.0 (82.0)	98.7 (19.6)	98.2 (20.1)	94.2 (7.2)
Refinement				
R1 (%)	22.15	29.27	17.11	N/A
wR2 (%)	56.51	68.51	46.18	N/A
Goodness of fit	2.181	1.908	1.449	N/A
**Crystal**	**Crystal 3**	**Crystal 3**	**Crystal 4**	**Crystal 4***
**Sweep**	**1**	**2**	**1**	**2**
Range of total fluence ($e^-/Å^2$)	0 – 2.7	2.7 – 5.4	0 – 2.7	2.7 – 5.4
Crystal system	Tetragonal	Tetragonal	Tetragonal	Tetragonal
Space group	$P4_32_12$	$P4_32_12$	$P4_32_12$	$P4_32_12$
Unit cell dimensions:				
a, b, c (Å)	7.07, 7.07, 29.01	7.07, 7.07, 28.89	7.06, 7.06, 29.07	N/A
α, β, γ (°)	90.000, 90.000, 90.000	90.000, 90.000, 90.000	90.000, 90.000, 90.000	N/A
Resolution (Å)	7.218 – 0.8 (0.9 – 0.8)	7.218 – 0.8 (0.9 – 0.8)	7.218 – 0.8 (0.9 – 0.8)	N/A
No. observed reflections	5420	5461	5365	N/A
No. unique reflections	950	945	949	N/A
R_{merge} (%)	21.3 (140.7)	38.8 (764.9)	20.2 (132.7)	N/A
R_{meas} (%)	23.7 (154.4)	43.1 (837.0)	22.3 (145.4)	N/A

Completeness (%)	99.3 (98.1)	99.4 (98.1)	99.4 (98.9)	N/A
I/σ	4.76 (1.10)	2.32 (0.17)	5.26 (1.17)	N/A
$CC_{1/2}$	98.8 (36.3)	99.1 (9.3)	99.2 (23.1)	N/A
Refinement				
R1 (%)	20.14	N/A	19.03	N/A
wR2 (%)	53.40	N/A	52.92	N/A
Goodness of fit	1.574	N/A	1.626	N/A
**Crystal**	**Crystal 5**	**Crystal 5***	**Crystal 6**	**Crystal 6**
**Sweep**	**1**	**2**	**1**	**2**
Range of total fluence ($e^-/Å^2$)	0 – 2.7	2.7 – 5.4	0 – 2.7	2.7 – 5.4
Crystal system	Tetragonal	Tetragonal	Tetragonal	Tetragonal
Space group	$P4_32_12$	$P4_32_12$	$P4_32_12$	$P4_32_12$
Unit cell dimensions:				
a, b, c (Å)	7.03, 7.03, 29.24	N/A	7.08, 7.08, 28.87	7.06, 7.06, 28.93
α, β, γ (°)	90.000, 90.000, 90.000	N/A	90.000, 90.000, 90.000	90.000, 90.000, 90.000
Resolution (Å)	7.218 – 0.8 (0.9 – 0.8)	N/A	7.218 – 0.8 (0.9 – 0.8)	7.218 – 0.8 (0.9 – 0.8)
No. observed reflections	5417	N/A	5564	5033
No. unique reflections	951	N/A	854	841
R_{merge} (%)	26.8 (98.1)	N/A	24.6 (155.8)	46.6 (848.4)
R_{meas} (%)	29.9 (108.0)	N/A	27.0 (169.0)	51.6 (924.0)
Completeness (%)	99.2 (97.5)	N/A	89.7 (87.9)	88.3 (83.6)
I/σ	4.10 (1.56)	N/A	4.39 (1.17)	1.79 (0.16)
$CC_{1/2}$	97.0 (35.3)	N/A	98.2 (7.1)	94.3 (13.1)
Refinement				
R1 (%)	22.24	N/A	18.29	N/A
wR2 (%)	55.72	N/A	48.45	N/A
Goodness of fit	1.593	N/A	1.356	N/A

Statistics of crystallographic data reduction and structure refinement obtained from Zn(II)-histidine crystals rotated at 1 degree/second while illuminated at a rate of 0.03 $e^-/Å^2s$, at 200 kV and room temperature, with an oscillation range of 1 degree per frame. Multiple sweeps of data (2.7 $e^-/Å^2$ total fluence delivered during each) were collected on each crystal, where data from 2 sweeps could typically be processed readily. Data from the second sweep acquired from crystals 4 and 5 (marked with *) could not be readily indexed.

Supplementary Table 15. Crystallographic statistics from fast tilt series (multiple sweeps) on Co(II)-porphyrin crystals at 0.01 $e^{-}/Å^{2}s$

**Crystal**	**Crystal 1**	**Crystal 1**	**Crystal 1**	**Crystal 1**
**Sweep**	**1**	**2**	**3**	**4**
Range of total fluence ($e^{-}/Å^{2}$)	0 – 1.0	1.0 – 2.0	2.0 – 3.0	3.0 – 4.0
Crystal system	Triclinic	Triclinic	Triclinic	Triclinic
Space group	$P\bar{1}$	$P\bar{1}$	$P\bar{1}$	$P\bar{1}$
Unit cell dimensions:				
a, b, c (Å)	9.66, 13.30, 13.49	9.67, 13.31, 13.49	9.67, 13.33, 13.50	9.68, 13.17, 13.42
α, β, γ (°)	87.374, 108.334, 111.875	87.155, 108.324, 111.897	87.108, 108.089, 111.772	87.403, 108.277, 111.720
Resolution (Å)	12.57 – 0.8 (0.9 – 0.8)	12.57 – 0.9 (1.0 – 0.9)	12.57 – 0.9 (1.0 – 0.9)	12.57 – 0.9 (1.0 – 0.9)
No. observed reflections	6171	6299	6103	6174
No. unique reflections	3267	3324	3278	3265
R_{merge} (%)	12.5 (59.1)	14.4 (53.2)	16.3 (69.0)	13.2 (55.2)
R_{meas} (%)	17.6 (83.5)	20.4 (75.2)	23.0 (97.6)	18.6 (78.0)
Completeness (%)	52.3 (52.4)	53.1 (53.1)	52.3 (53.5)	52.9 (52.3)
I/σ	3.32 (1.25)	2.99 (1.31)	2.40 (1.00)	2.99 (1.34)
$CC_{1/2}$	98.2 (20.4)	97.1 (18.7)	97.6 (11.9)	97.5 (18.5)
**Crystal**	**Crystal 1**	**Crystal 1**	**Crystal 2**	**Crystal 2**
**Sweep**	**5**	**6**	**1**	**2**
Range of total fluence ($e^{-}/Å^{2}$)	4.0 – 5.0	5.0 – 6.0	0 – 1.0	1.0 – 2.0
Crystal system	Triclinic	Triclinic	Triclinic	Triclinic
Space group	$P\bar{1}$	$P\bar{1}$	$P\bar{1}$	$P\bar{1}$
Unit cell dimensions:				
a, b, c (Å)	9.67, 13.32, 13.49	9.68, 13.24, 13.46	9.66, 13.43, 13.40	9.56, 13.34, 13.41
α, β, γ (°)	87.307, 108.009, 111.746	87.250, 108.192, 112.028	87.554, 108.189, 111.865	87.466, 108.171, 111.790
Resolution (Å)	12.57 – 0.8 (0.9 – 0.8)	12.57 – 0.8 (0.9 – 0.8)	12.57 – 0.8 (0.9 – 0.8)	12.57 – 0.8 (0.9 – 0.8)
No. observed reflections	5987	5461	6126	6012
No. unique reflections	3198	3277	3318	3275
R_{merge} (%)	15.5 (70.9)	14.1 (58.6)	12.4 (26.8)	11.5 (29.2)
R_{meas} (%)	21.9 (100.2)	20.0 (82.9)	17.6 (37.9)	16.2 (41.3)
Completeness (%)	51.0 (52.6)	52.8 (52.9)	53.0 (49.7)	52.6 (50.6)
I/σ	2.58 (1.00)	2.75 (1.17)	3.65 (3.45)	3.84 (3.21)
$CC_{1/2}$	98.5 (9.2)	98.4 (15.9)	97.8 (48.3)	99.0 (43.0)
**Crystal**	**Crystal 2**	**Crystal 2**	**Crystal 2**	**Crystal 2**

Sweep	3	4	5	6
Range of total fluence ($e^-/Å^2$)	2.0 – 3.0	3.0 – 4.0	4.0 – 5.0	5.0 – 6.0
Crystal system	Triclinic	Triclinic	Triclinic	Triclinic
Space group	$P\bar{1}$	$P\bar{1}$	$P\bar{1}$	$P\bar{1}$
Unit cell dimensions:				
a, b, c (Å)	9.66, 13.29, 13.40	9.66, 13.36, 13.40	9.66, 13.36, 13.40	9.65, 13.26, 13.40
α, β, γ (°)	87.674, 108.112, 111.863	87.704, 108.111, 111.902	87.629, 108.119, 111.931	87.726, 108.061, 111.759
Resolution (Å)	12.57 – 0.8 (0.9 – 0.8)	12.57 – 0.8 (0.9 – 0.8)	12.57 – 0.8 (0.9 – 0.8)	12.57 – 0.8 (0.9 – 0.8)
No. observed reflections	6046	6014	5638	6093
No. unique reflections	3280	3287	3179	3300
R_{merge} (%)	13.7 (31.5)	14.4 (31.3)	14.4 (29.4)	15.9 (30.5)
R_{meas} (%)	19.4 (44.6)	20.3 (44.3)	20.4 (41.5)	22.5 (43.1)
Completeness (%)	52.9 (49.9)	52.8 (51.3)	51.1 (50.1)	53.3 (51.2)
I/σ	3.45 (3.09)	3.19 (3.06)	3.33 (3.12)	3.11 (3.00)
$CC_{1/2}$	98.1 (36.2)	97.9 (38.8)	98.9 (36.3)	97.2 (31.5)
**Crystal**	**Crystal 3**	**Crystal 3**	**Crystal 3**	**Crystal 3**
**Sweep**	**1**	**2**	**3**	**4**
Range of total fluence ($e^-/Å^2$)	0 – 1.0	1.0 – 2.0	2.0 – 3.0	3.0 – 4.0
Crystal system	Triclinic	Triclinic	Triclinic	Triclinic
Space group	$P\bar{1}$	$P\bar{1}$	$P\bar{1}$	$P\bar{1}$
Unit cell dimensions:				
a, b, c (Å)	9.67, 13.34, 13.41	9.68, 13.25, 13.40	9.68, 13.25, 13.40	9.68, 13.26, 13.40
α, β, γ (°)	86.940, 108.449, 111.810	87.359, 108.574, 111.928	87.321, 108.467, 111.866	87.430, 108.546, 112.008
Resolution (Å)	12.57 – 0.8 (0.9 – 0.8)	12.57 – 0.8 (0.9 – 0.8)	12.57 – 0.8 (0.9 – 0.8)	12.57 – 0.8 (0.9 – 0.8)
No. observed reflections	6152	6255	6080	6152
No. unique reflections	3306	3254	3229	3247
R_{merge} (%)	11.7 (55.9)	11.7 (59.0)	11.1 (55.6)	12.3 (60.0)
R_{meas} (%)	16.5 (79.0)	16.5 (76.1)	15.6 (78.6)	17.3 (84.9)
Completeness (%)	53.1 (52.8)	52.7 (52.6)	52.2 (52.5)	52.6 (52.6)
I/σ	3.38 (1.25)	3.53 (1.29)	3.65 (1.32)	3.18 (1.21)
$CC_{1/2}$	98.8 (22.6)	98.9 (30.7)	98.7 (22.0)	98.7 (22.6)
**Crystal**	**Crystal 3**	**Crystal 3**		
**Sweep**	**5**	**6**		
Range of total fluence ($e^-/Å^2$)	4.0 – 5.0	5.0 – 6.0		

Crystal system	Triclinic	Triclinic		
Space group	$P\bar{1}$	$P\bar{1}$		
Unit cell dimensions:				
a, b, c (Å)	9.67, 13.30, 13.41	9.68, 13.27, 13.40		
α, β, γ (°)	87.152, 108.439, 111.901	87.263, 108.443, 111.947		
Resolution (Å)	12.57 – 0.8 (0.9 – 0.8)	12.57 – 0.8 (0.9 – 0.8)		
No. observed reflections	6159	6337		
No. unique reflections	3246	3296		
R_{merge} (%)	10.4 (57.2)	11.0 (60.6)		
R_{meas} (%)	14.6 (81.0)	15.6 (85.7)		
Completeness (%)	52.2 (52.6)	53.3 (53.0)		
I/σ	3.94 (1.25)	3.62 (1.13)		
$CC_{1/2}$	99.0 (16.9)	99.2 (19.8)		

Statistics of crystallographic data reduction and structure refinement obtained from Co(II)-porphyrin crystals rotated at 1 degree/second while illuminated at a rate of 0.01 $e^-/Å^2s$, at 200 kV and room temperature, with an oscillation range of 1 degree per frame. Multiple sweeps of data (1.0 $e^-/Å^2$ total fluence delivered during each) were collected on each crystal, where data from at least 6 sweeps could be processed readily without obvious detriment to data reduction statistics. Analysis of refinement statistics is omitted, as 100° tilt series do not reliably afford sufficiently high completeness to yield *ab initio* phasing solutions with direct methods from single crystals of Co(II)-porphyrin.

Supplementary Table 16. Crystallographic statistics from fast tilt series (multiple sweeps) on Co(II)-porphyrin crystals at 0.03 e⁻/Å²s

**Crystal**	**Crystal 1**	**Crystal 1**	**Crystal 1**	**Crystal 1**
**Sweep**	**1**	**2**	**3**	**4**
Range of total fluence ($e^-/Å^2$)	0 – 3.0	3.0 – 6.0	6.0 – 9.0	9.0 – 12.0
Crystal system	Triclinic	Triclinic	Triclinic	Triclinic
Space group	$P\bar{1}$	$P\bar{1}$	$P\bar{1}$	$P\bar{1}$
Unit cell dimensions:				
a, b, c (Å)	9.64, 13.31, 13.38	9.64, 13.31, 13.37	9.64, 13.34, 13.40	9.64, 13.32, 13.37
α, β, γ (°)	87.906, 108.056, 111.694	88.000, 108.014, 111.730	87.722, 108.007, 111.813	88.029, 107.920, 111.779
Resolution (Å)	12.57 – 0.8 (0.9 – 0.8)	12.57 – 0.8 (0.9 – 0.8)	12.57 – 0.8 (0.9 – 0.8)	12.57 – 0.8 (0.9 – 0.8)
No. observed reflections	6178	6142	6096	6179
No. unique reflections	3286	3294	3329	3285
R_{merge} (%)	9.5 (56.9)	10.6 (59.4)	11.0 (68.9)	9.6 (70.0)
R_{meas} (%)	13.4 (1.29)	15.0 (84.0)	15.5 (97.5)	13.6 (98.9)
Completeness (%)	53.0 (52.4)	53.2 (53.4)	53.6 (53.5)	53.0 (52.7)
I/σ	3.94 (1.29)	3.48 (1.16)	3.21 (1.01)	3.65 (1.00)
$CC_{1/2}$	98.9 (30.0)	98.9 (22.8)	98.7 (19.1)	99.3 (19.0)
**Crystal**	**Crystal 1**	**Crystal 1**	**Crystal 2**	**Crystal 2**
**Sweep**	**5**	**6**	**1**	**2**
Range of total fluence ($e^-/Å^2$)	12.0 – 15.0	15.0 – 18.0	0 – 3.0	3.0 – 6.0
Crystal system	Triclinic	Triclinic	Triclinic	Triclinic
Space group	$P\bar{1}$	$P\bar{1}$	$P\bar{1}$	$P\bar{1}$
Unit cell dimensions:				
a, b, c (Å)	9.64, 13.36, 13.39	9.65, 13.32, 13.38	9.72, 13.31, 13.34	9.72, 13.37, 13.34
α, β, γ (°)	87.767, 107.858, 111.768	87.935, 107.868, 111.867	87.498, 108.501, 111.810	87.513, 108.483, 111.732
Resolution (Å)	12.57 – 0.8 (0.9 – 0.8)	12.57 – 0.8 (0.9 – 0.8)	12.57 – 0.8 (0.9 – 0.8)	12.57 – 0.8 (0.9 – 0.8)
No. observed reflections	6051	6294	6141	6128
No. unique reflections	3293	3318	3270	3309
R_{merge} (%)	11.0 (66.9)	10.5 (77.0)	9.8 (40.1)	9.7 (47.7)
R_{meas} (%)	15.6 (94.6)	14.9 (108.9)	13.8 (56.7)	13.7 (67.5)
Completeness (%)	52.9 (52.5)	53.5 (53.6)	52.6 (52.7)	53.0 (53.5)
I/σ	3.17 (1.04)	3.32 (0.98)	4.03 (1.79)	3.76 (1.41)
$CC_{1/2}$	98.8 (20.2)	99.1 (10.6)	97.5 (48.3)	98.8 (40.5)
**Crystal**	**Crystal 2**	**Crystal 2**	**Crystal 2**	**Crystal 2**

**Sweep**	**3**	**4**	**5**	**6**
Range of total fluence ($e^-/Å^2$)	6.0 – 9.0	9.0 – 12.0	12.0 – 15.0	15.0 – 18.0
Crystal system	Triclinic	Triclinic	Triclinic	Triclinic
Space group	$P\bar{1}$	$P\bar{1}$	$P\bar{1}$	$P\bar{1}$
Unit cell dimensions:				
a, b, c (Å)	9.72, 13.34, 13.34	9.73, 13.21, 13.34	9.72, 13.20, 13.34	9.72, 13.76, 13.34
α, β, γ (°)	87.580, 108.428, 111.753	87.451, 108.363, 112.013	87.400, 108.324, 112.095	87.616, 108.306, 111.876
Resolution (Å)	12.57 – 0.8 (0.9 – 0.8)	12.57 – 0.8 (0.9 – 0.8)	12.57 – 0.8 (0.9 – 0.8)	12.57 – 0.8 (0.9 – 0.8)
No. observed reflections	6034	6201	6069	6166
No. unique reflections	3240	3268	3292	3317
R_{merge} (%)	11.5 (52.8)	10.5 (54.5)	12.3 (64.6)	11.4 (69.3)
R_{meas} (%)	16.3 (74.7)	14.8 (77.0)	17.4 (91.3)	16.1 (98.1)
Completeness (%)	52.0 (52.6)	53.0 (52.8)	53.4 (52.8)	53.5 (53.3)
I/σ	3.18 (1.29)	3.54 (1.29)	2.90 (1.10)	3.08 (1.03)
$CC_{1/2}$	97.3 (33.7)	98.7 (39.2)	97.5 (22.0)	98.0 (25.8)
**Crystal**	**Crystal 3**	**Crystal 3**	**Crystal 3**	**Crystal 3**
**Sweep**	**1**	**2**	**3**	**4**
Range of total fluence ($e^-/Å^2$)	0 – 3.0	3.0 – 6.0	6.0 – 9.0	9.0 – 12.0
Crystal system	Triclinic	Triclinic	Triclinic	Triclinic
Space group	$P\bar{1}$	$P\bar{1}$	$P\bar{1}$	$P\bar{1}$
Unit cell dimensions:				
a, b, c (Å)	9.66, 13.30, 13.37	9.67, 13.29, 13.37	9.67, 13.30, 13.37	9.67, 13.30, 13.37
α, β, γ (°)	87.559, 107.912, 112.000	87.483, 107.873, 111.912	87.516, 107.829, 111.991	87.551, 107.780, 112.057
Resolution (Å)	12.57 – 0.8 (0.9 – 0.8)	12.57 – 0.8 (0.9 – 0.8)	12.57 – 0.8 (0.9 – 0.8)	12.57 – 0.8 (0.9 – 0.8)
No. observed reflections	5914	5902	5894	5911
No. unique reflections	3077	3092	3065	3069
R_{merge} (%)	6.3 (30.0)	6.1 (29.7)	6.2 (33.0)	6.4 (33.5)
R_{meas} (%)	8.9 (42.4)	8.7 (42.0)	8.8 (46.6)	9.1 (47.4)
Completeness (%)	49.7 (51.4)	49.9 (51.5)	49.5 (51.6)	49.5 (51.4)
I/σ	6.64 (2.31)	6.43 (2.23)	6.61 (2.29)	6.31 (36.4)
$CC_{1/2}$	99.6 (81.2)	99.6 (82.7)	99.7 (77.8)	99.7 (75.9)
**Crystal**	**Crystal 3**	**Crystal 3**	**Crystal 4**	**Crystal 4**
**Sweep**	**5**	**6**	**1**	**2**
Range of total fluence ($e^-/Å^2$)	6.0 – 9.0	9.0 – 12.0	0 – 3.0	3.0 – 6.0

Crystal system	Triclinic	Triclinic	Triclinic	Triclinic
Space group	$P\bar{1}$	$P\bar{1}$	$P\bar{1}$	$P\bar{1}$
Unit cell dimensions:				
a, b, c (Å)	9.67, 13.31, 13.38	9.67, 13.31, 13.38	9.68, 13.33, 13.33	9.67, 13.34, 13.33
α, β, γ (°)	87.649, 107.790, 111.956	87.687, 107.745, 112.048	87.531, 107.962, 112.187	87.534, 107.905, 112.285
Resolution (Å)	12.57 – 0.8 (0.9 – 0.8)	12.57 – 0.8 (0.9 – 0.8)	12.57 – 0.8 (0.9 – 0.8)	12.57 – 0.8 (0.9 – 0.8)
No. observed reflections	5839	6001	5766	6141
No. unique reflections	3096	3123	3046	3179
R_{merge} (%)	6.9 (37.0)	6.9 (39.2)	11.3 (42.7)	10.3 (42.0)
R_{meas} (%)	9.7 (52.3)	9.7 (55.4)	15.9 (60.4)	14.6 (59.4)
Completeness (%)	49.8 (51.4)	50.3 (51.9)	49.2 (49.9)	51.4 (51.8)
I/σ	5.72 (2.25)	5.95 (2.22)	4.12 (1.66)	4.38 (1.64)
$CC_{1/2}$	99.7 (68.3)	99.7 (65.5)	99.2 (33.8)	99.4 (38.7)
**Crystal**	**Crystal 4**	**Crystal 4**	**Crystal 4**	**Crystal 4**
**Sweep**	**3**	**4**	**5**	**6**
Range of total fluence ($e^-/Å^2$)	6.0 – 9.0	9.0 – 12.0	12.0 – 15.0	15.0 – 18.0
Crystal system	Triclinic	Triclinic	Triclinic	Triclinic
Space group	$P\bar{1}$	$P\bar{1}$	$P\bar{1}$	$P\bar{1}$
Unit cell dimensions:				
a, b, c (Å)	9.69, 13.34, 13.34	9.68, 13.34, 13.33	9.69, 13.34, 13.33	9.69, 13.35, 13.33
α, β, γ (°)	87.755, 108.040, 112.141	87.359, 108.574, 111.928	87.596, 107.782, 112.256	87.625, 107.751, 112.294
Resolution (Å)	12.57 – 0.8 (0.9 – 0.8)	12.57 – 0.8 (0.9 – 0.8)	12.57 – 0.8 (0.9 – 0.8)	12.57 – 0.8 (0.9 – 0.8)
No. observed reflections	6112	6182	6104	6027
No. unique reflections	3183	3205	3176	3131
R_{merge} (%)	10.2 (43.2)	10.0 (41.2)	10.1 (46.2)	9.7 (49.9)
R_{meas} (%)	14.4 (61.1)	14.2 (1.62)	14.2 (65.3)	13.7 (70.5)
Completeness (%)	51.3 (52.3)	51.7 (52.1)	51.2 (51.7)	50.5 (51.8)
I/σ	4.38 (1.66)	4.25 (1.62)	4.37 (1.50)	4.67 (1.47)
$CC_{1/2}$	99.4 (32.9)	99.3 (42.2)	99.4 (31.7)	99.6 (35.9)

Statistics of crystallographic data reduction and structure refinement obtained from Co(II)-porphyrin crystals rotated at 1 degree/second while illuminated at a rate of 0.03 $e^-/Å^2s$, at 200 kV and room temperature, with an oscillation range of 1 degree per frame. Multiple sweeps of data (3.0 $e^-/Å^2$ total fluence delivered during each) were collected on each crystal, where data from at least 6 sweeps could be processed readily without obvious detriment to data reduction statistics.

Supplementary Table 17. Online locations of data deposition (Zenodo)

Data type	Sample	Accelerating Voltage (kV)	Deposition Code	DOI
Static crystal diffraction series	Biotin	200	10989028	https://doi.org/10.5281/zenodo.10989028
Static crystal diffraction series	Cu(II)-serine	200	10989028	https://doi.org/10.5281/zenodo.10989028
Static crystal diffraction series	Zn(II)-histidine	200	10989028	https://doi.org/10.5281/zenodo.10989028
Static crystal diffraction series	Zn(II)-methionine	200	10989360	https://doi.org/10.5281/zenodo.10989360
Static crystal diffraction series	Co(II)-porphyrin	200	10989502	https://doi.org/10.5281/zenodo.10989502
Static crystal diffraction series (high fluence)	Co(II)-porphyrin	200	10989575	https://doi.org/10.5281/zenodo.10989575
Static crystal diffraction series	AVAAGA	200	10999587	https://doi.org/10.5281/zenodo.10999587
Static crystal diffraction series	Thiostrepton	200	10999587	https://doi.org/10.5281/zenodo.10999587
Static crystal diffraction series	Proteinase K	200	10999587	https://doi.org/10.5281/zenodo.10999587
Static crystal diffraction series	Biotin	300	10995034	https://doi.org/10.5281/zenodo.10995034
Static crystal diffraction series	Cu(II)-serine	300	10995034	https://doi.org/10.5281/zenodo.10995034
Static crystal diffraction series	Zn(II)-methionine	300	10995034	https://doi.org/10.5281/zenodo.10995034
Static crystal diffraction series	Zn(II)-histidine	300	10995139	https://doi.org/10.5281/zenodo.10995139
Static crystal diffraction series	Co(II)-porphyrin	300	10995139	https://doi.org/10.5281/zenodo.10995139
Static crystal diffraction series	AVAAGA	300	10995139	https://doi.org/10.5281/zenodo.10995139
Static crystal diffraction series	Thiostrepton	300	10999589	https://doi.org/10.5281/zenodo.10999589
Static crystal diffraction series (extra thick carbon support film)	Biotin	300	10995169	https://doi.org/10.5281/zenodo.10995169
Static crystal diffraction series (extra thick carbon support film)	Zn(II)-methionine	300	10995169	https://doi.org/10.5281/zenodo.10995169
Static crystal diffraction series (extra thick carbon support film)	Zn(II)-histidine	300	10995169	https://doi.org/10.5281/zenodo.10995169

Tilt series (0.09 deg/s slow rotation)	Biotin	200	10993554	https://doi.org/10.5281/zenodo.10993554
Tilt series (0.09 deg/s slow rotation)	Zn(II)-methionine	200	10993554	https://doi.org/10.5281/zenodo.10993554
Tilt series (0.09 deg/s slow rotation)	Co(II)-porphyrin	200	10993554	https://doi.org/10.5281/zenodo.10993554
Tilt series (1 deg/s, multiple sweeps)	Biotin	200	10994067	https://doi.org/10.5281/zenodo.10994067
Tilt series (1 deg/s, multiple sweeps)	Zn(II)-methionine	200	10994067	https://doi.org/10.5281/zenodo.10994067
Tilt series (1 deg/s, multiple sweeps)	Zn(II)-Histidine	200	10994330	https://doi.org/10.5281/zenodo.10994330
Tilt series (1 deg/s, multiple sweeps)	Co(II)-porphyrin	200	10994691	https://doi.org/10.5281/zenodo.10994691
TEM image series/diffraction snapshots for bend contour analysis	Biotin	200	13308651	https://doi.org/10.5281/zenodo.12208651
TEM image series/diffraction snapshots for bend contour analysis	Zn(II)-methionine	200	13308651	https://doi.org/10.5281/zenodo.12208651
TEM image series/diffraction snapshots for bend contour analysis	Co(II)-porphryin	200	13308651	https://doi.org/10.5281/zenodo.12208651
TEM image series/diffraction snapshots for bend contour analysis (100K dry and frozen-hydrated)	AVAAGA	200	13308651	https://doi.org/10.5281/zenodo.12208651
Tilt series data for structure determination	Biotin	200	10994067	https://doi.org/10.5281/zenodo.10994067
Tilt series data for structure determination	Cu(II)-serine	200	11043930	https://doi.org/10.5281/zenodo.11043930
Tilt series data for structure determination	Zn(II)-methionine	200	11043930	https://doi.org/10.5281/zenodo.11043930
Tilt series data for structure determination	Zn(II)-histidine	200	11043930	https://doi.org/10.5281/zenodo.11043930
Tilt series data for structure determination	Co(II)-porphryin	300	11043930	https://doi.org/10.5281/zenodo.11043930

Deposition information for all electron diffraction and imaging datasets analyzed in this report. All datasets of the same type, acquired under the same conditions, are uploaded in the deposition records on Zenodo noted here, and are readily accessible for download.

Supplementary movie legends

All supplementary movies are converted from their native MRC file format, binned to 256x256 pixel arrays (for diffraction movies) or 205x205 arrays (for imaging movies), and presented in GIF format for viewing.

Supplementary movie 1. Electron diffraction series from a representative static biotin nanocrystal at 200 kV and 293 K.

Supplementary movie 2. Electron diffraction series from a representative static Cu(II)-serine nanocrystal at 200 kV and 293 K.

Supplementary movie 3. Electron diffraction series from a representative static Zn(II)-methionine nanocrystal at 200 kV and 293 K.

Supplementary movie 4. Electron diffraction series from a representative static Zn(II)-histidine nanocrystal at 200 kV and 293 K.

Supplementary movie 5. Electron diffraction series from a representative static Co(II)-porphyrin nanocrystal at 200 kV and 293 K.

Supplementary movie 6. Fast TEM imaging series from a representative static biotin nanocrystal at 200 kV and 293 K revealing motion of bend contours.

Supplementary movie 7. Fast TEM imaging series from a representative static Zn(II)-methionine nanocrystal at 200 kV and 293 K revealing motion of bend contours.

Supplementary movie 8. Fast TEM imaging series from a representative static Co(II)-porphyrin nanocrystal at 200 kV and 293 K revealing static bend contours.

Supplementary movie 9. Fast TEM imaging series from a representative static dry AVAAGA nanocrystal at 200 kV and 100 K revealing motion of bend contours.

Supplementary movie 10. Fast TEM imaging series from a representative static AVAAGA nanocrystal embedded in vitreous ice at 200 kV and 100 K revealing motion of bend contours.

Supplementary figures

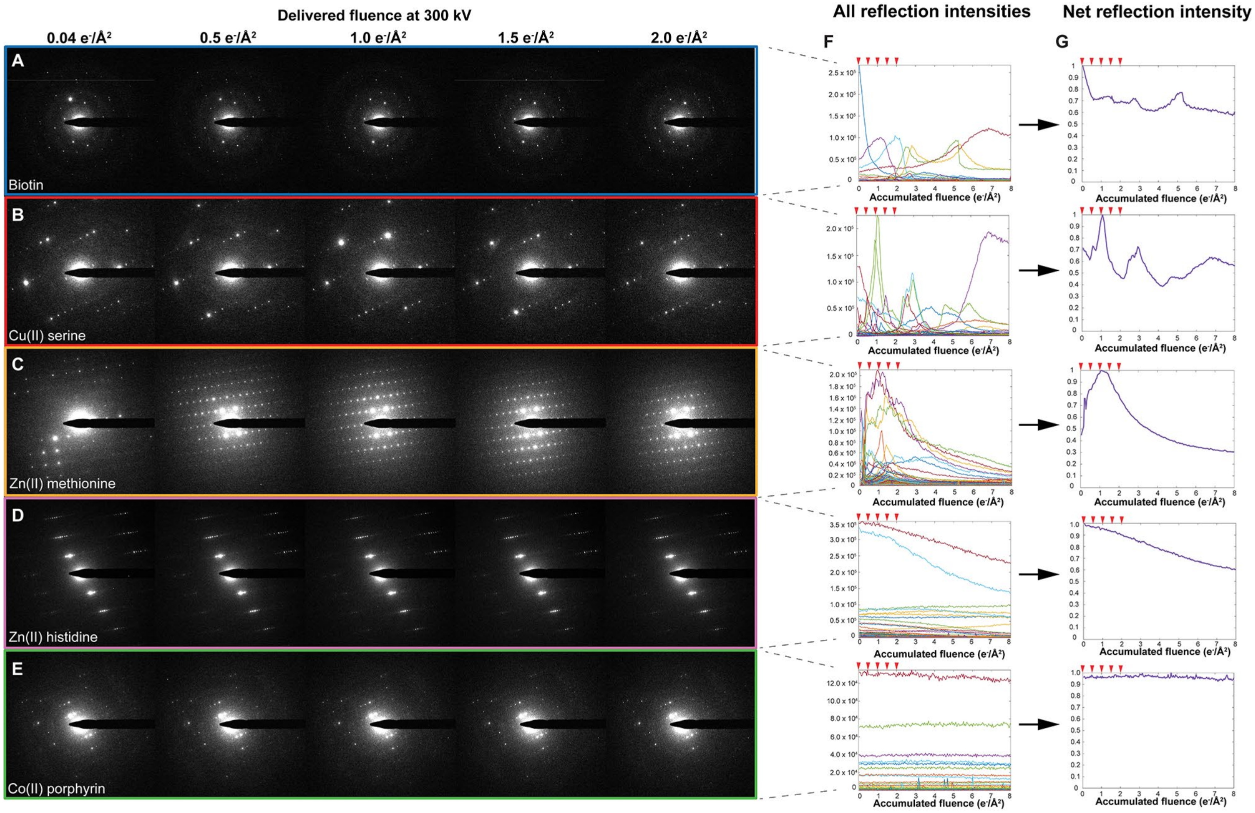

Figure S1. Changes in diffraction from stationary, room-temperature, crystals at 300 kV. Initial diffraction pattern, and frames acquired following an accumulated fluence of 0.5, 1.0, 1.5, and 2.0 e$^-$/Å^2, from left to right, for a representative crystal each of biotin (A), Cu(II) serine (B), Zn(II) methionine (C), Zn(II) histidine (D), and Co(II) meso-tetraphenyl porphine (E). Measured intensities of each reflection as a function of accumulated fluence for each of these diffraction series (F), and the total reflection intensity in each (normalized to its maximum value) as a function of accumulated fluence (G).

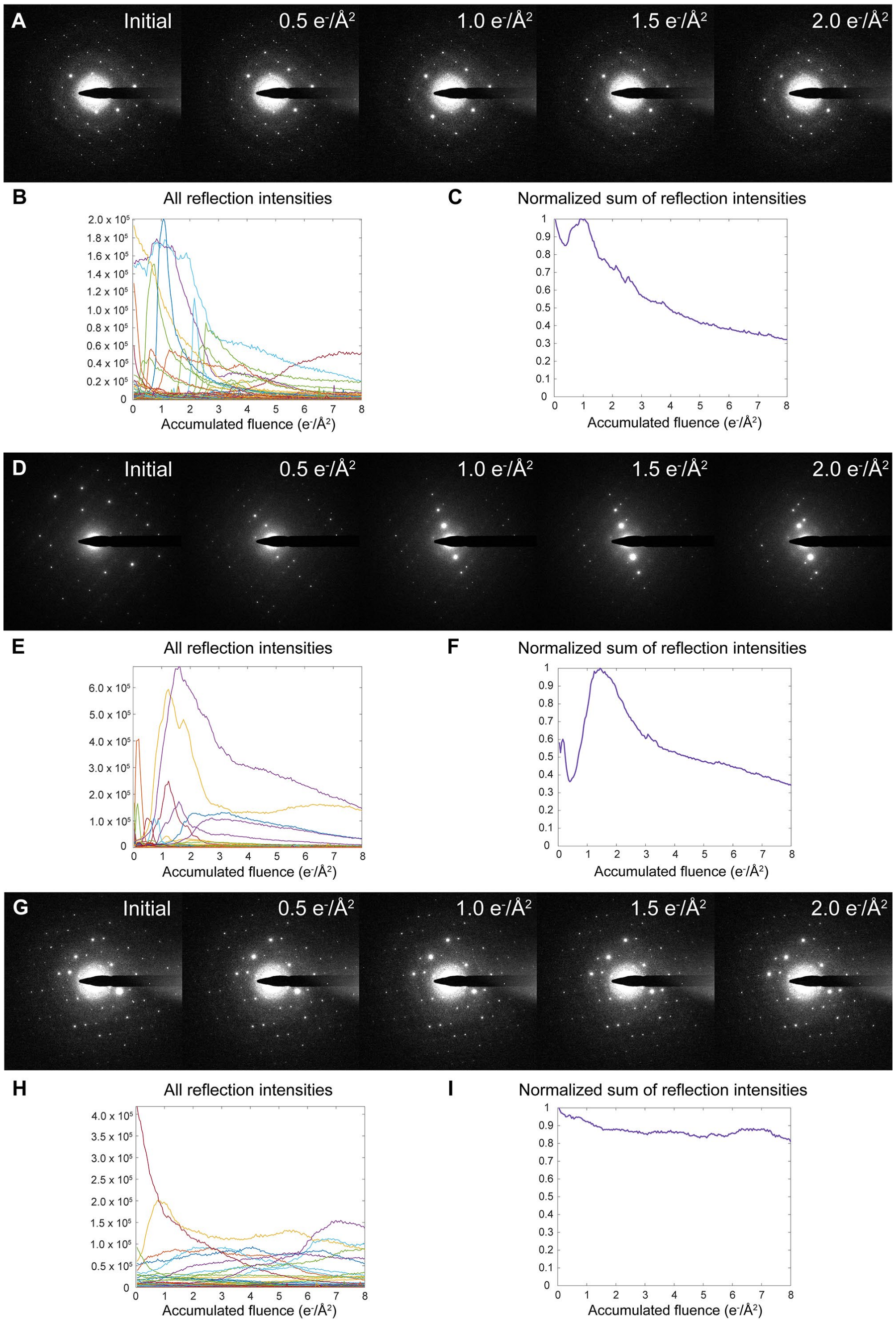

Figure S2. Changes in diffraction from stationary crystals on extra thick support films (3-4 nm thick carbon on 25-50 nm thick formvar). Initial diffraction pattern, and frames acquired following an accumulated fluence of 0.5, 1.0, 1.5, and 2.0 $e^-/Å^2$, from left to right, for a representative crystal of biotin (A), plots of each reflection's intensity as a function of accumulated fluence (B), and plot of the sum of all reflection intensities as a function of accumulated fluence, normalized to its maximum value (C). The same is shown for a representative crystal of Zn(II) methionine (D-F) and Zn(II) histidine (H-I) on the same support film type, all at room temperature and 300 kV.

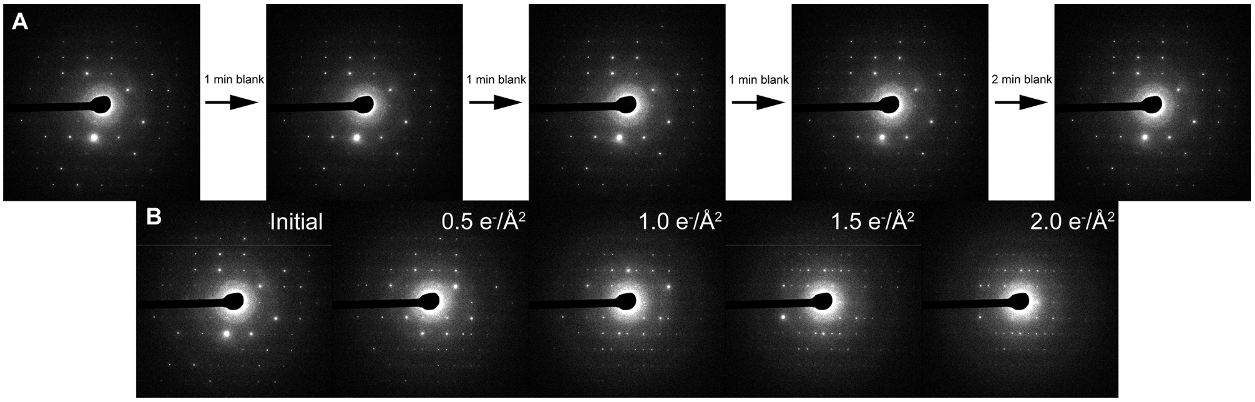

Figure S3. Non-monotonic diffraction intensity fluctuations are not due to unaccounted-for sample stage motion. A sub-micron thick biotin crystal was exposed for 1 second to an electron beam delivering approximately 0.01 $e^-/Å^2$ during that interval four times, separated by 1 minute intervals with the beam blanked, and a fifth time following an additional 2 minute wait period with the beam blanked. Minimal changes are observed from one diffraction snapshot to the next (A). The stationary crystal was then continuously illuminated while collecting a diffraction series. The initial diffraction pattern, and those captured after the delivery of an additional 0.5, 1.0, 1.5, and 2.0 $e^-/Å^2$, are shown, and display clear non-monotonic fluctuations in reflection intensities, and changes in the set of reflections intersecting the Ewald sphere (B).

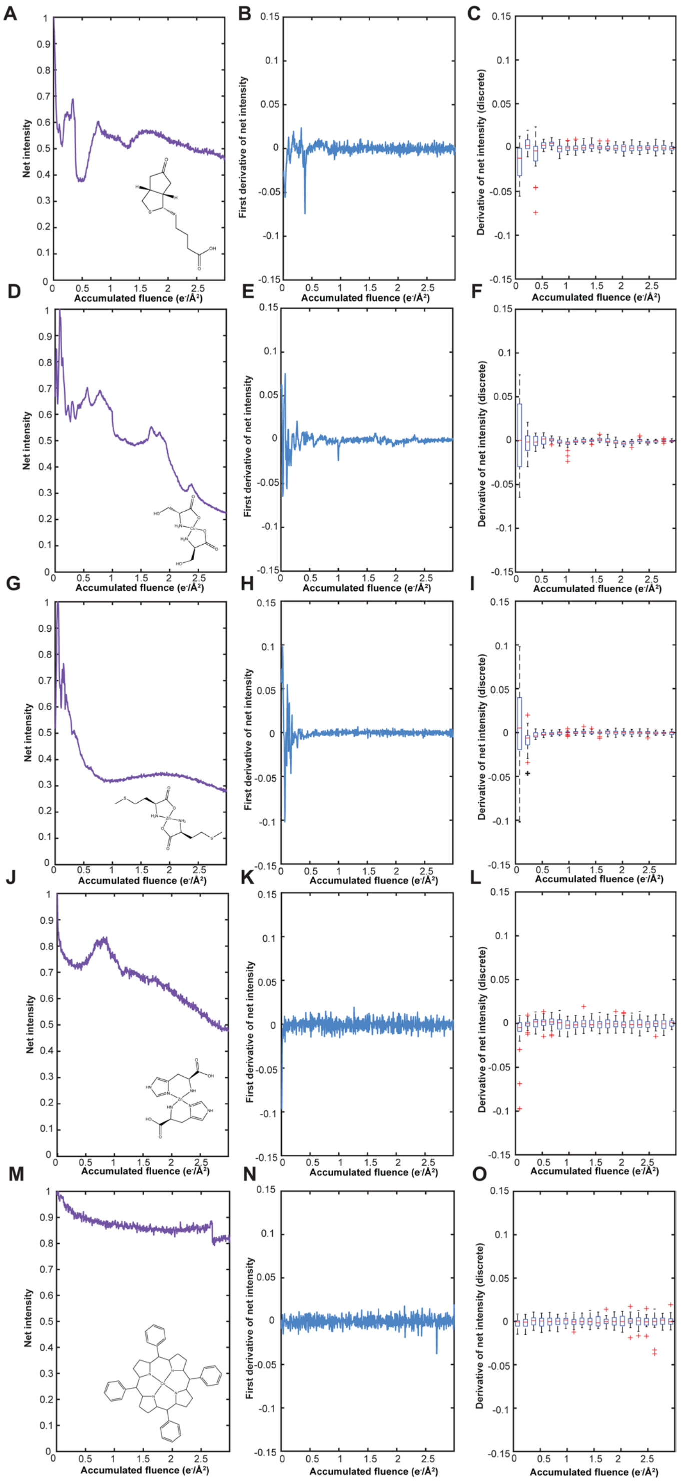

Figure S4. Quantifying degree of dose-dependent non-monotonic intensity fluctuation in electron diffraction series from stationary crystals. For a representative crystal each of biotin (A-C), Cu(II) serine (D-F), Zn(II) methionine (G-I), Zn(II) histidine (J-L), and Co(II) meso-tetraphenyl porphine (M-O): plot of the total reflection intensity, plot of the first derivative of the summed reflection intensity with respect to fluence, and discretized plot of values of the derivative profile in 20 equally sized bins between 0 and 3.0 $e^{-}/Å^{2}$ along the fluence axis.

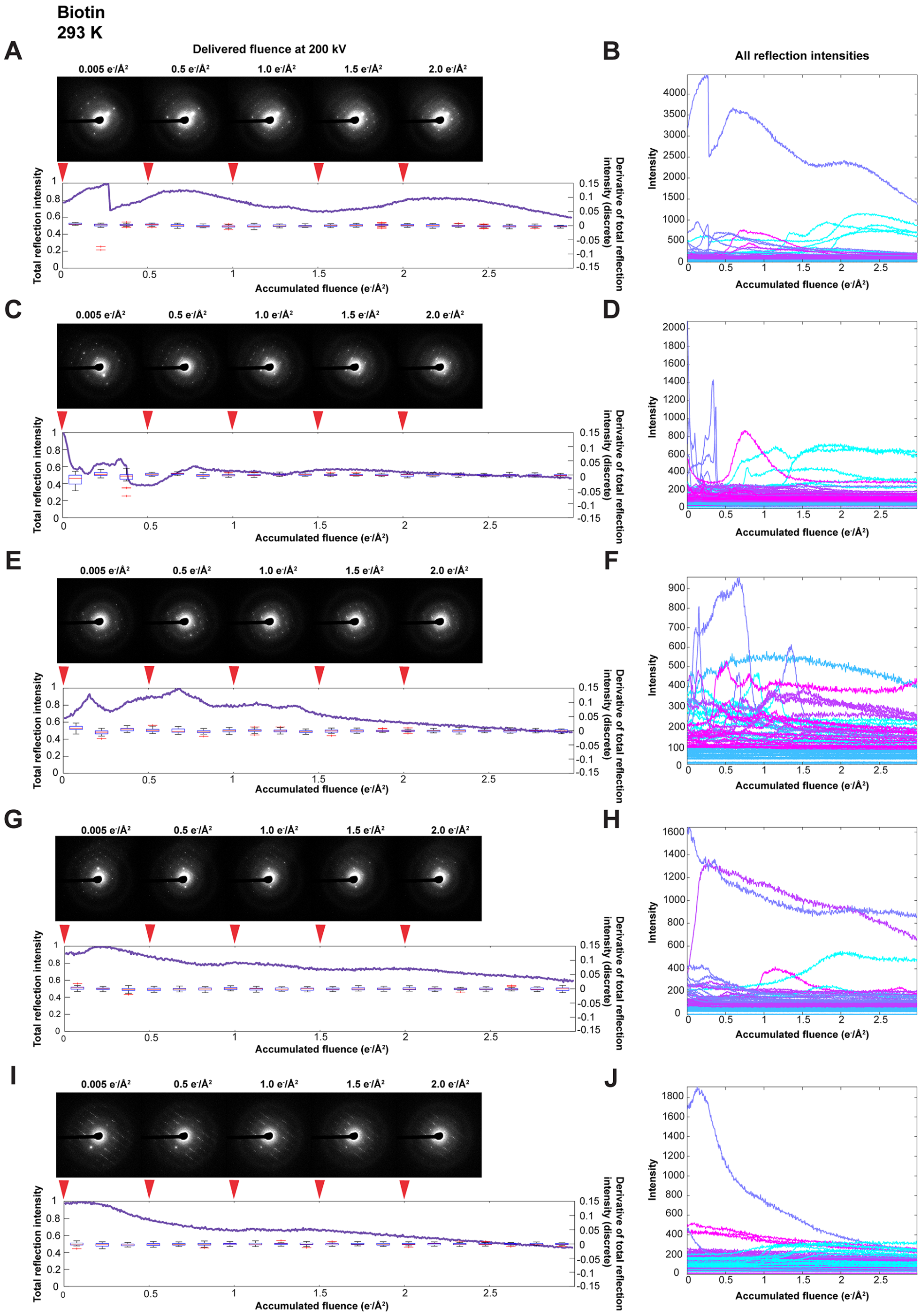

Figure S5. Changes in reflection intensity in response to electron beam exposure for stationary, room-temperature, biotin crystals at 200 kV. For five different crystals diffracted under the same conditions, initial diffraction pattern, and frames acquired following an accumulated fluence of 0.5, 1.0, 1.5, and 2.0 e$^-$/Å^2, from left to right, plot of normalized total reflection intensity (considering the brightest 20% of detected reflections, left-hand y-axis) as a function of accumulated fluence, and discretized plot of values of the derivative of this curve with respect to fluence (right-hand y-axis) (A,C,E,G,I). Plots of all reflection intensities for each diffraction series, with traces colored by k-means cluster (B,D,F,H,J).

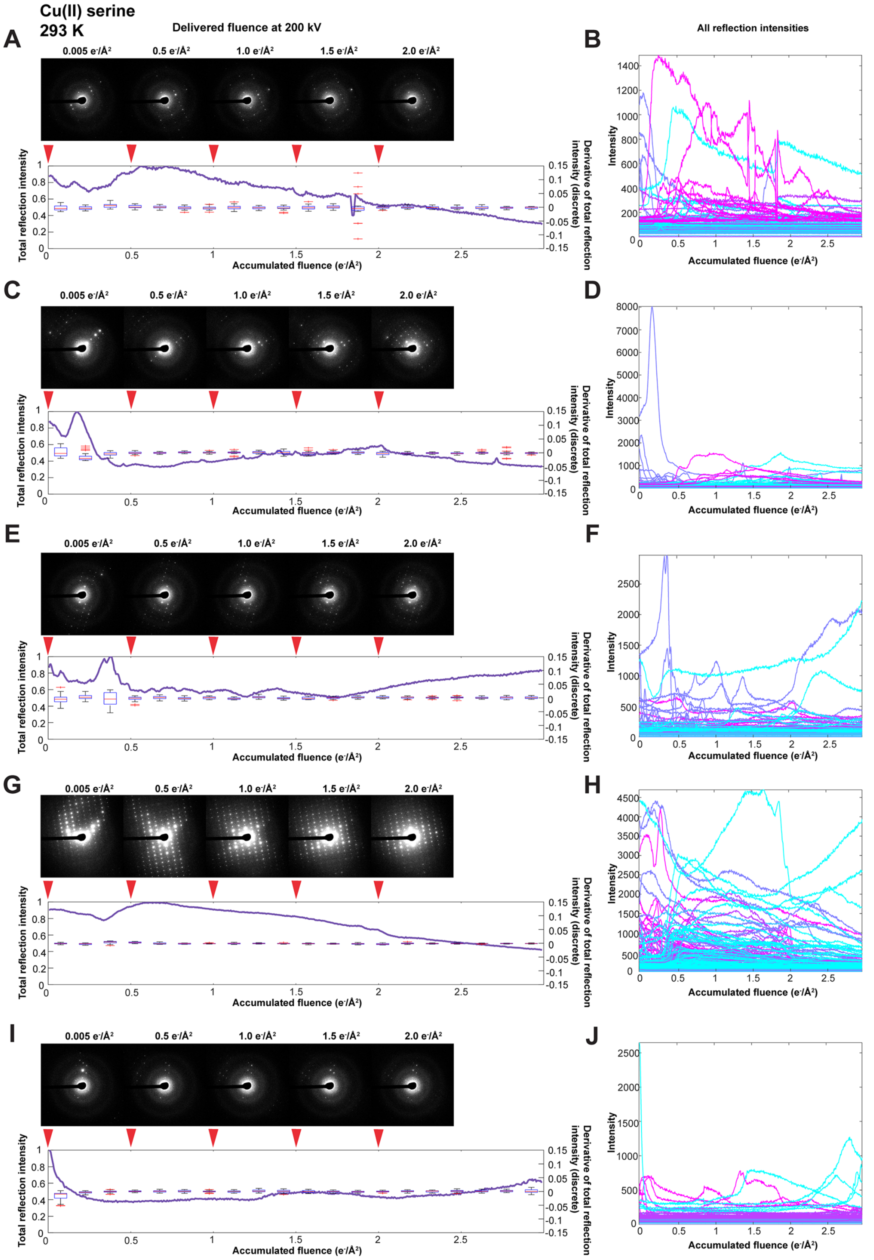

Figure S6. Changes in reflection intensity in response to electron beam exposure for stationary, room-temperature, Cu(II) serine crystals at 200 kV. For five different crystals diffracted under the same conditions, initial diffraction pattern, and frames acquired following an accumulated fluence of 0.5, 1.0, 1.5, and 2.0 e$^-$/Å^2, from left to right, plot of normalized total reflection intensity (considering the brightest 20% of detected reflections, left-hand y-axis) as a function of accumulated fluence, and discretized plot of values of the derivative of this curve with respect to fluence (right-hand y-axis) (A,C,E,G,I). Plots of all reflection intensities for each diffraction series, with traces colored by k-means cluster (B,D,F,H,J).

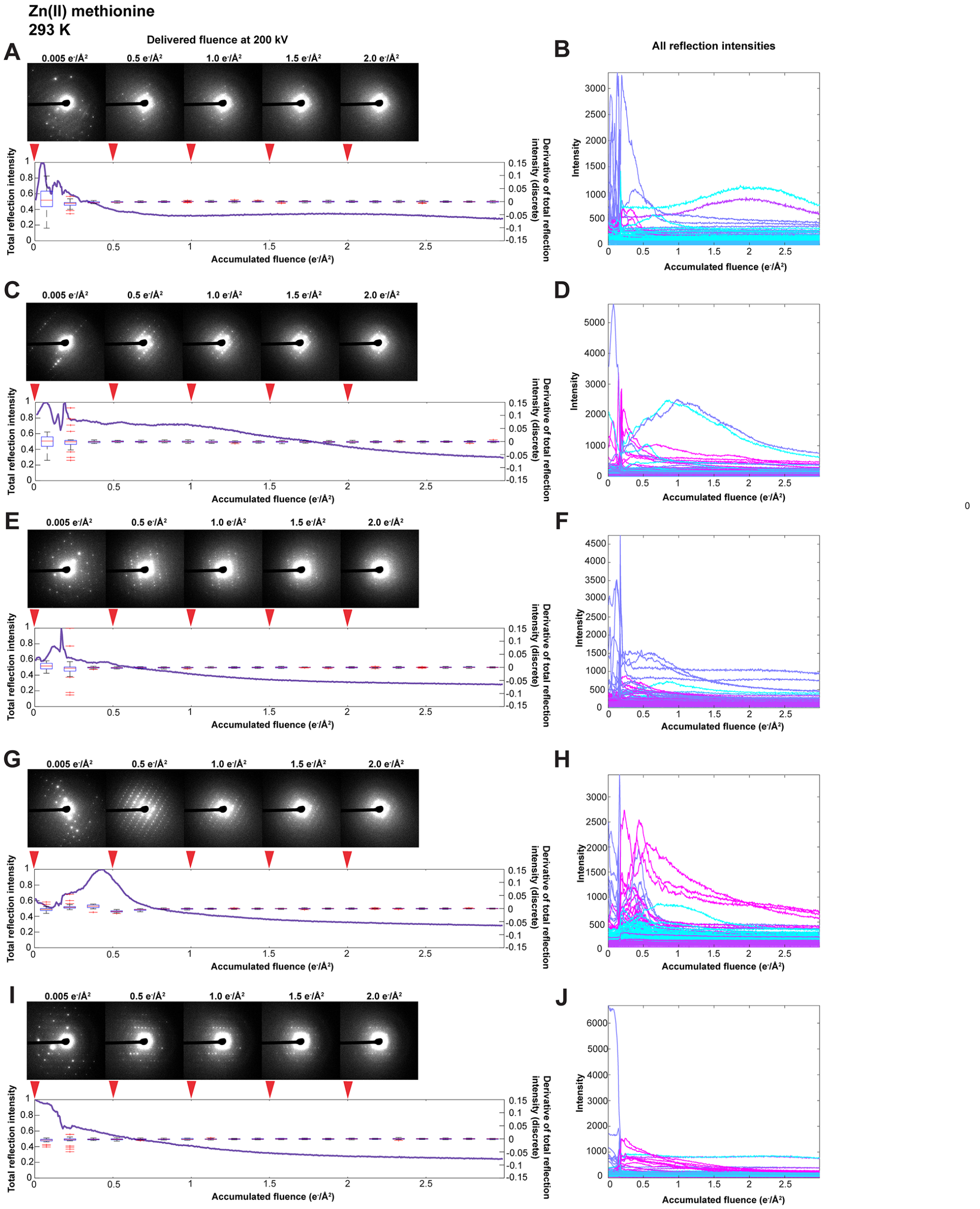

Figure S7. Changes in reflection intensity in response to electron beam exposure for stationary, room-temperature, Zn(II) methionine crystals at 200 kV. For five different crystals diffracted under the same conditions, initial diffraction pattern, and frames acquired following an accumulated fluence of 0.5, 1.0, 1.5, and 2.0 $e^{-}/Å^2$, from left to right, plot of normalized total reflection intensity (considering the brightest 20% of detected reflections, left-hand y-axis) as a function of accumulated fluence, and discretized plot of values of the derivative of this curve with respect to fluence (right-hand y-axis) (A,C,E,G,I). Plots of all reflection intensities for each diffraction series, with traces colored by k-means cluster (B,D,F,H,J).

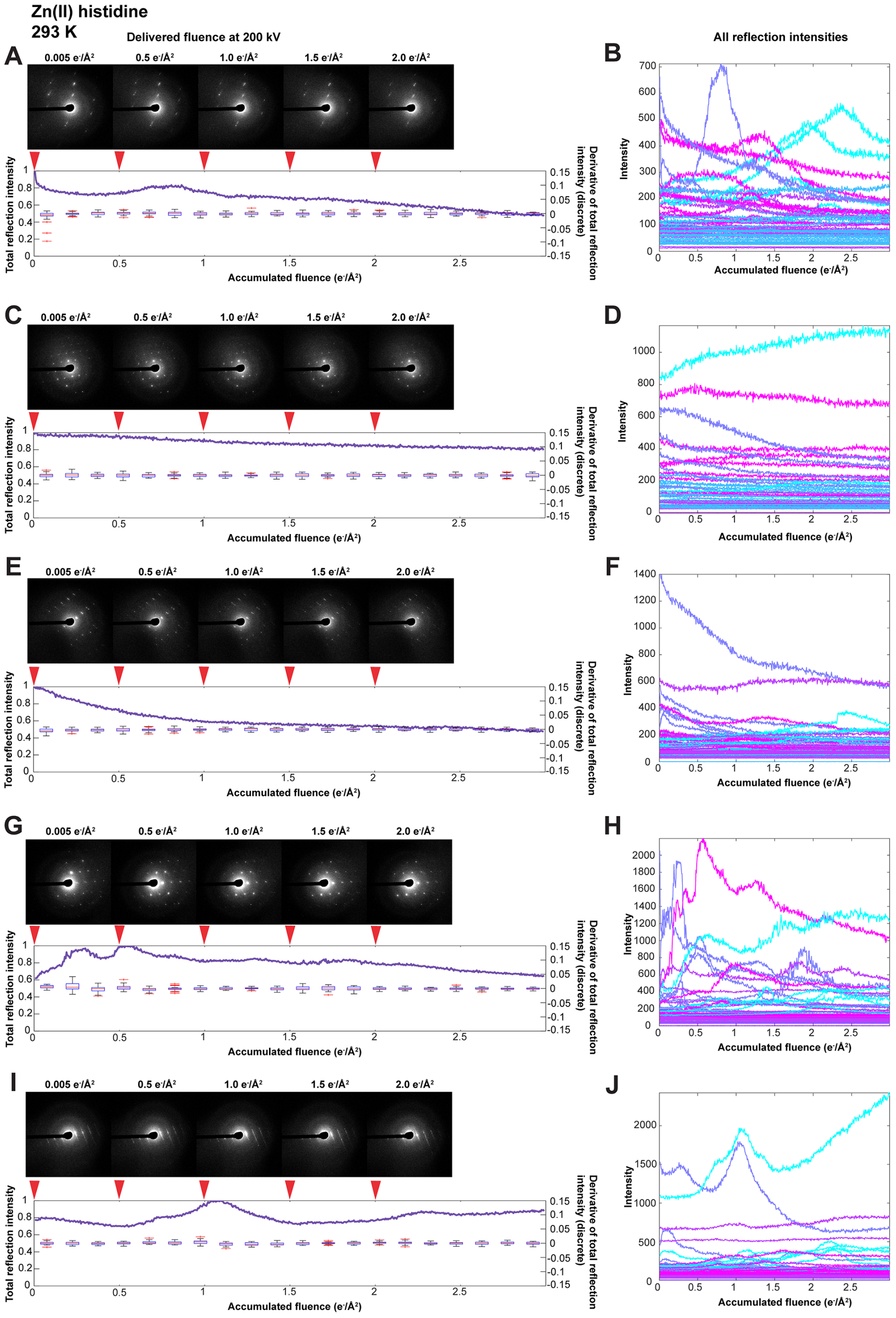

Figure S8. Changes in reflection intensity in response to electron beam exposure for stationary, room-temperature, Zn(II) histidine crystals at 200 kV. For five different crystals diffracted under the same conditions, initial diffraction pattern, and frames acquired following an accumulated fluence of 0.5, 1.0, 1.5, and 2.0 e-/Å^2, from left to right, plot of normalized total reflection intensity (considering the brightest 20% of detected reflections, left-hand y-axis) as a function of accumulated fluence, and discretized plot of values of the derivative of this curve

with respect to fluence (right-hand y-axis) (A,C,E,G,I). Plots of all reflection intensities for each diffraction series, with traces colored by k-means cluster (B,D,F,H,J).

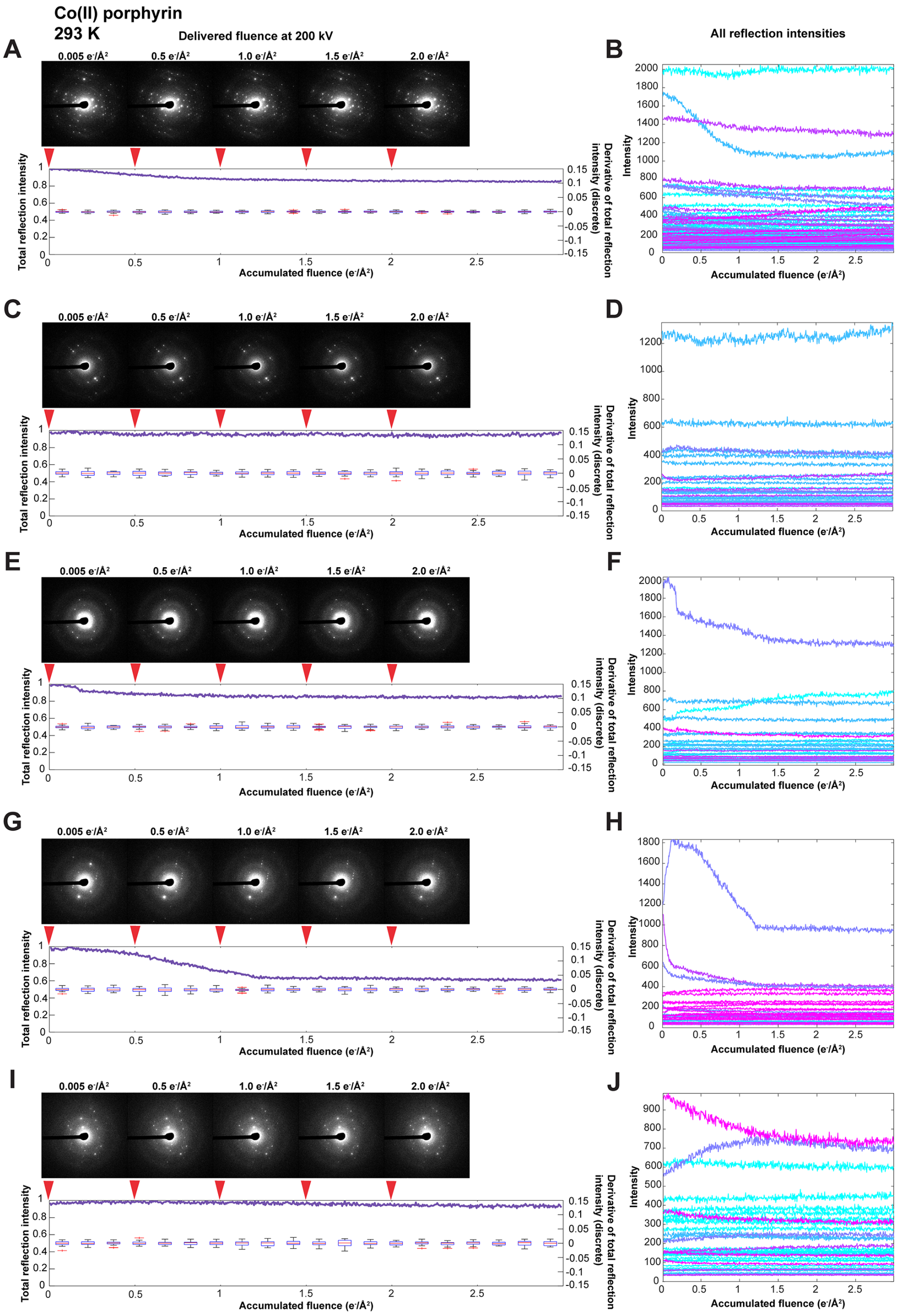

Figure S9. Changes in reflection intensity in response to electron beam exposure for stationary, room-temperature, Co(II) porphyrin crystals at 200 kV. For five different crystals diffracted under the same conditions, initial diffraction pattern, and frames acquired following an accumulated fluence of 0.5, 1.0, 1.5, and 2.0 $e^-/Å^2$, from left to right, plot of normalized total reflection intensity (considering the brightest 20% of detected reflections, left-hand y-axis) as a function of accumulated fluence, and discretized plot of values of the derivative of this curve

with respect to fluence (right-hand y-axis) (A,C,E,G,I). Plots of all reflection intensities for each diffraction series, with traces colored by k-means cluster (B,D,F,H,J).

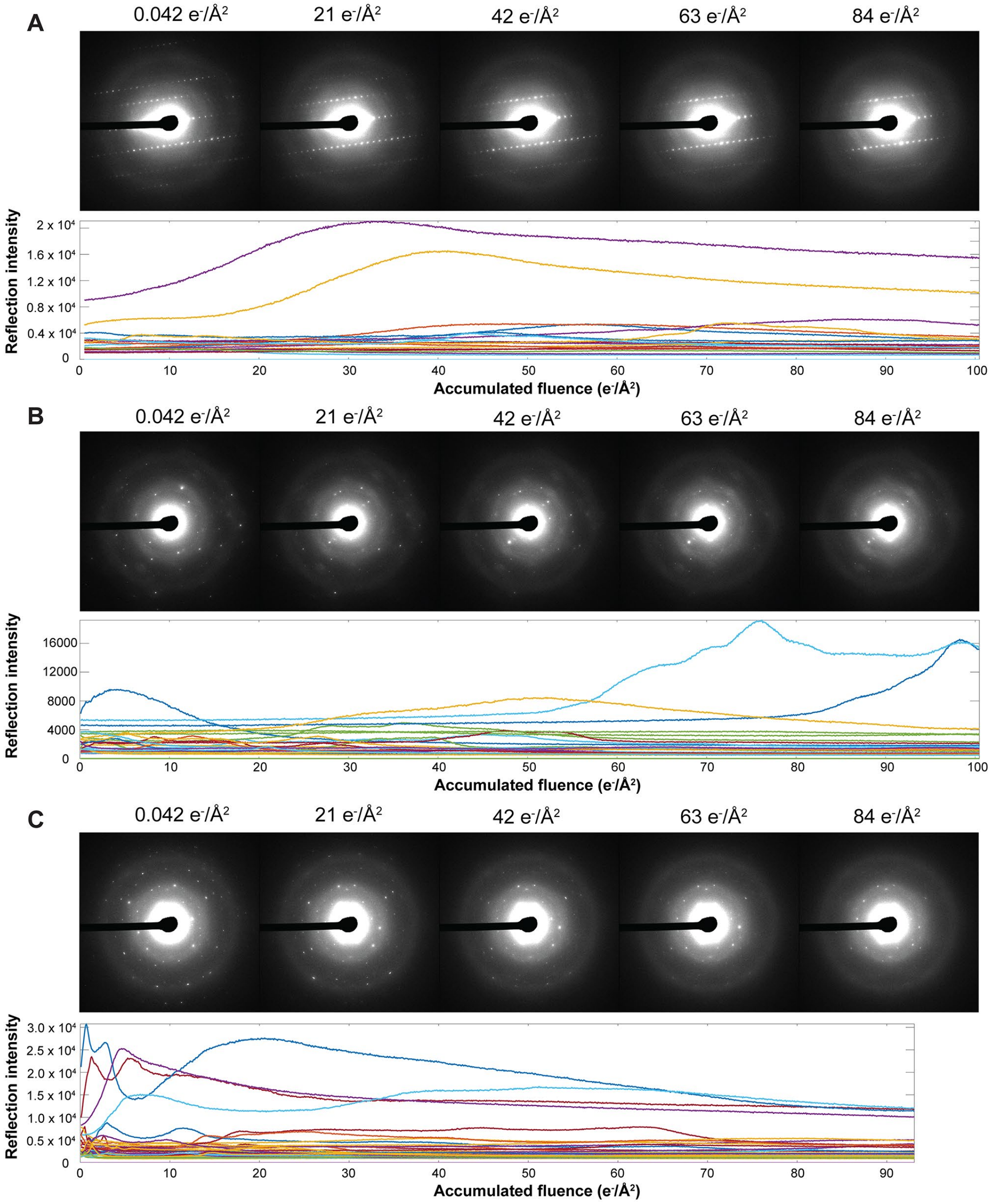

Figure S10. Beam-induced reorientation of Co(II) porphyrin crystals at 293 K in response to high total fluence. For three representative Co(II) porphyrin crystals illuminated with an incident flux density of 0.084 $e^-/Å^2s$, series of diffraction patterns and plots of brightest 20% of reflections' intensity as a function of fluence.

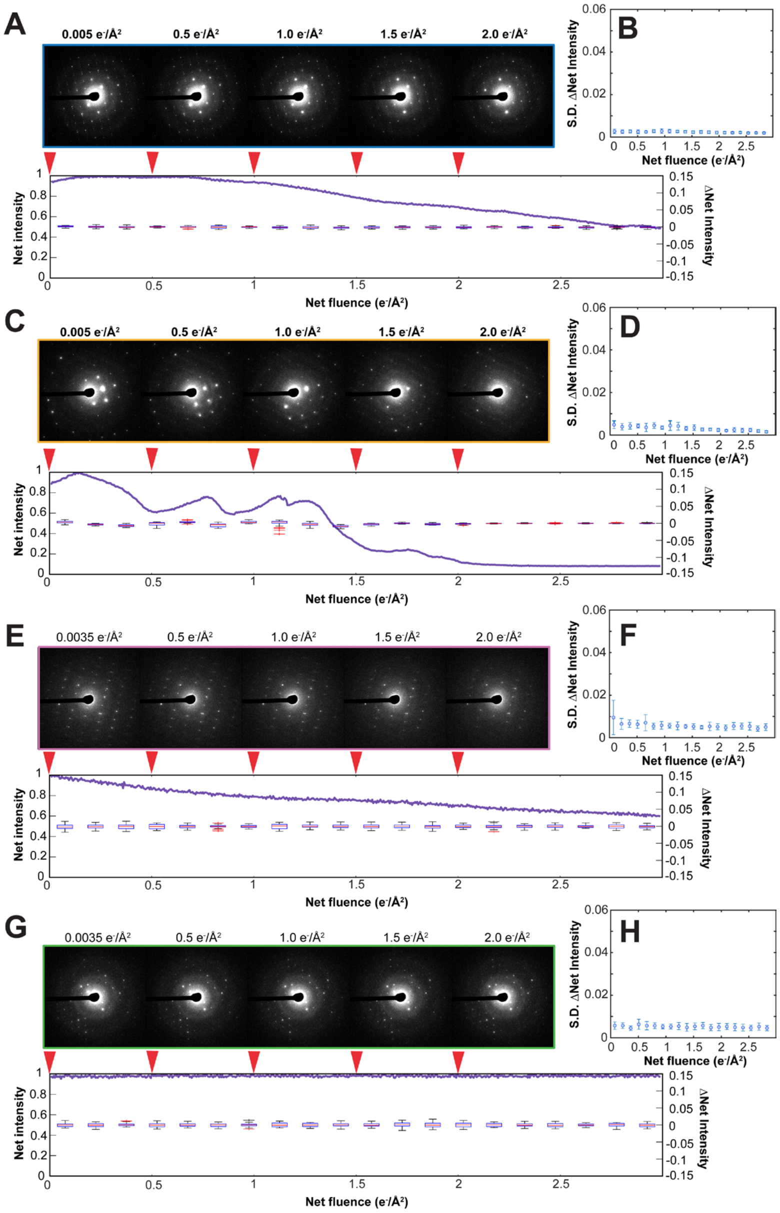

Figure S11. Non-monotonic decay in diffraction from stationary crystals at 200 kV under cryogenic temperatures (100 K). For a representative crystal each of biotin (A), Zn(II) methionine (C), Zn(II) histidine (E), and Co(II) meso-tetraphenyl porphyrin (G): initial diffraction pattern, and frames acquired following an accumulated fluence of 0.5, 1.0, 1.5, and 2.0 $e^{-}/Å^{2}$, from left to right, plot of normalized total reflection intensity (considering the brightest 20% of detected reflections, left-hand y-axis) as a function of accumulated fluence, and discretized plot of values of the derivative of this curve with respect to fluence (right-hand y-axis). Plots of the average standard deviation of first derivative values in each such discrete bin for all crystals of biotin (B), Zn(II) methionine (D), Zn(II) histidine (F), and Co(II) meso-tetraphenyl porphyrin (H) interrogated at 200 kV and room temperature. Error bars equal to one standard deviation from the mean.

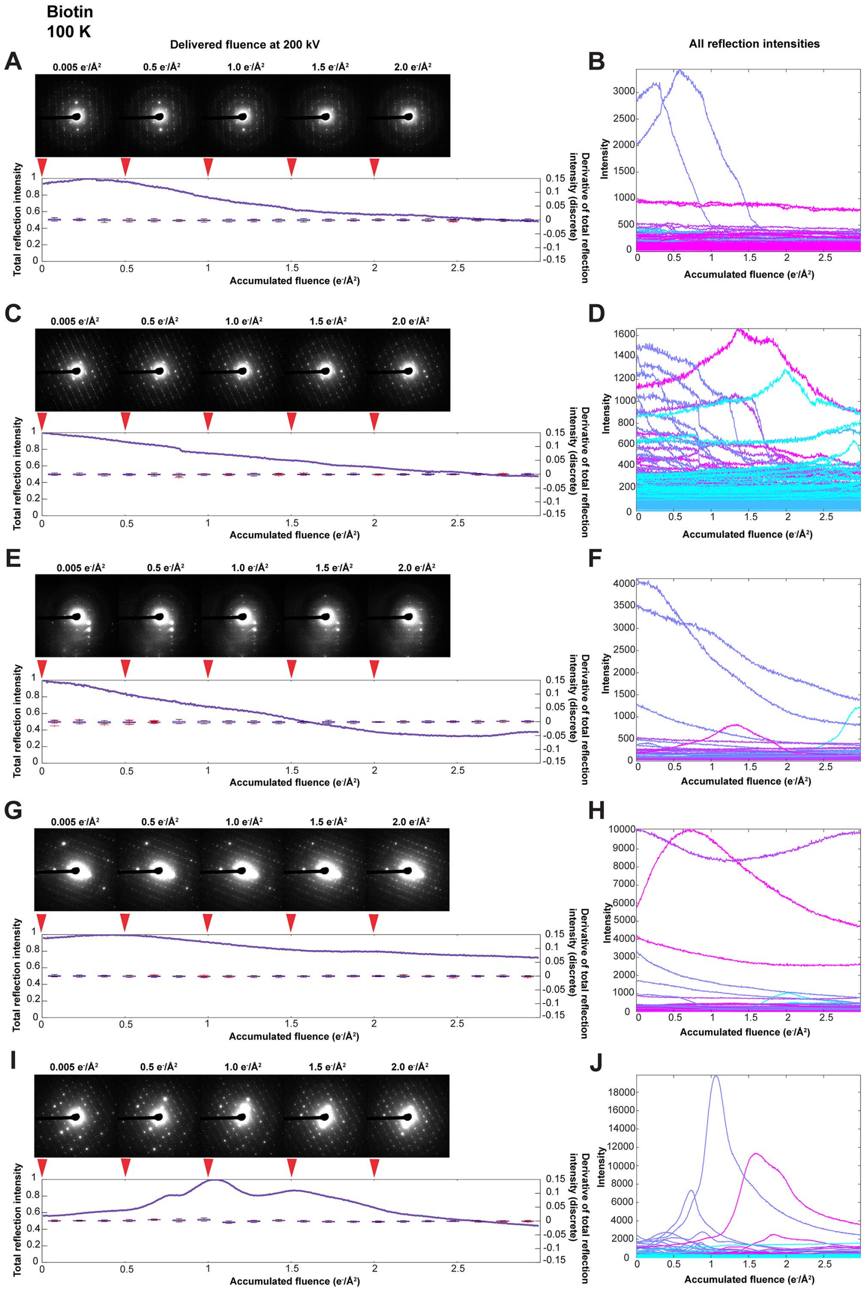

Figure S12. Changes in reflection intensity in response to electron beam exposure for stationary, biotin crystals at 200 kV and cryogenic (100 K) temperature. For five different crystals diffracted under the same conditions, initial diffraction pattern, and frames acquired following an accumulated fluence of 0.5, 1.0, 1.5, and 2.0 e-/Å^2, from left to right, plot of normalized total reflection intensity (considering the brightest 20% of detected reflections, left-hand y-axis) as a function of accumulated fluence, and discretized plot of values of the derivative of this curve with respect to fluence (right-hand y-axis) (A,C,E,G,I). Plots of all reflection intensities for each diffraction series, with traces colored by k-means cluster (B,D,F,H,J).

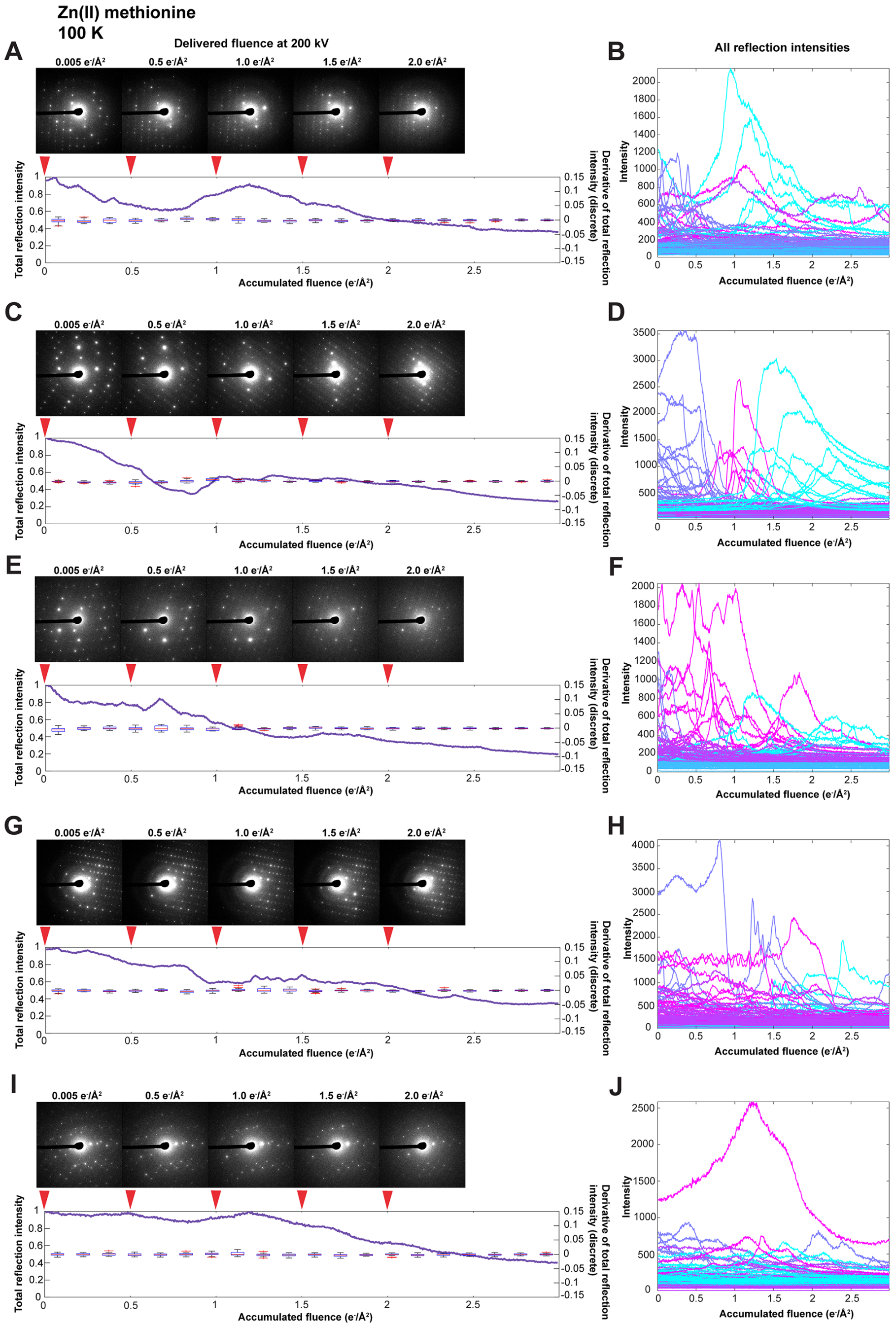

Figure S13. Changes in reflection intensity in response to electron beam exposure for stationary, Zn(II) methionine crystals at 200 kV and cryogenic (100 K) temperature. For five different crystals diffracted under the same conditions, initial diffraction pattern, and frames acquired following an accumulated fluence of 0.5, 1.0, 1.5, and 2.0 e-/Å^2, from left to right, plot of normalized total reflection intensity (considering the brightest 20% of detected reflections, left-hand y-axis) as a function of accumulated fluence, and discretized plot of values of the derivative of this curve with respect to fluence (right-hand y-axis) (A,C,E,G,I). Plots of all reflection intensities for each diffraction series, with traces colored by k-means cluster (B,D,F,H,J).

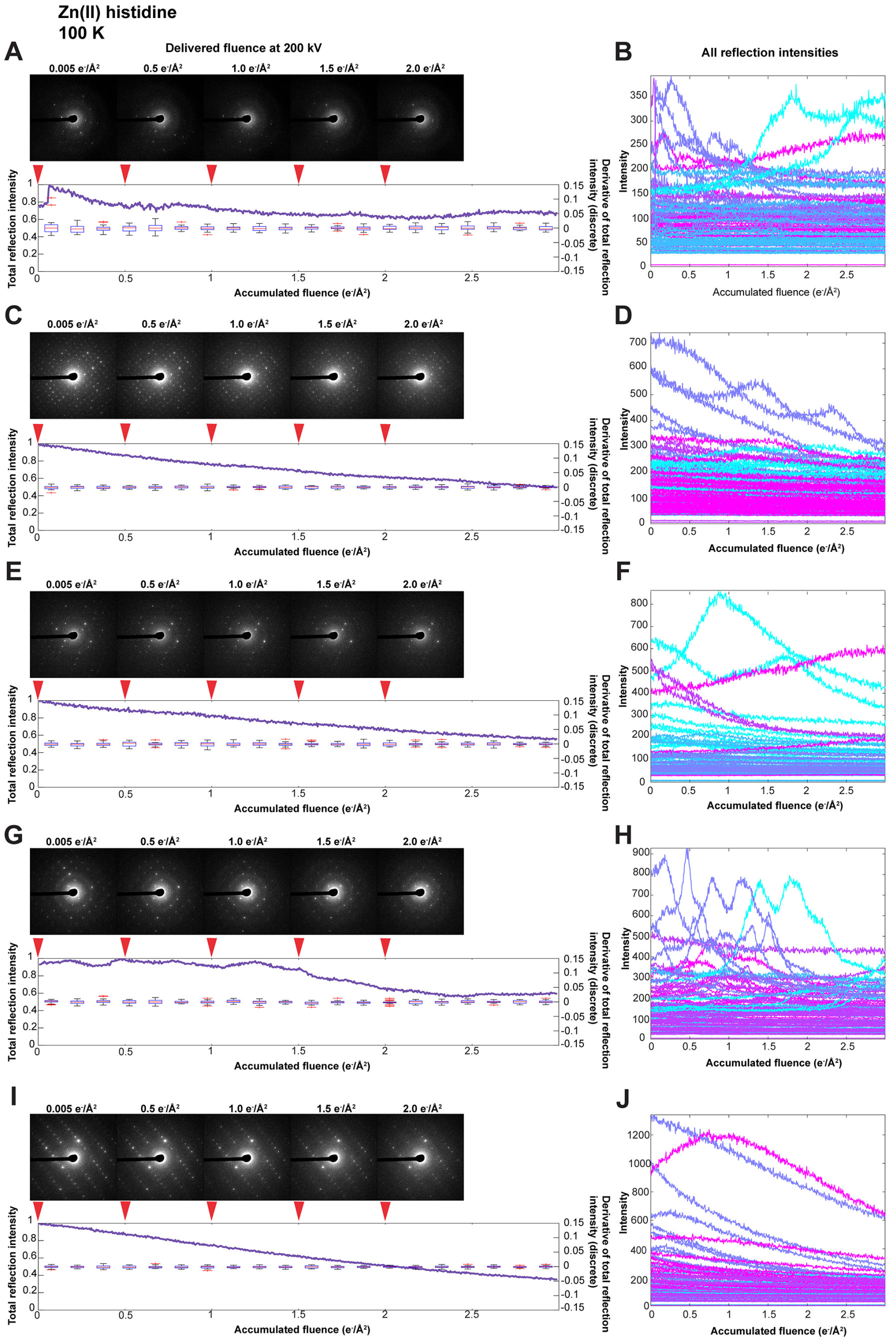

Figure S14. Changes in reflection intensity in response to electron beam exposure for stationary Zn(II) histidine crystals at 200 kV and cryogenic (100 K) temperature. For five different crystals diffracted under the same conditions, initial diffraction pattern, and frames acquired following an accumulated fluence of 0.5, 1.0, 1.5, and 2.0 e-/Å^2, from left to right, plot of normalized total reflection intensity (considering the brightest 20% of detected reflections, left-hand y-axis) as a function of accumulated fluence, and discretized plot of values of the derivative of this curve with respect to fluence (right-hand y-axis) (A,C,E,G,I). Plots of all reflection intensities for each diffraction series, with traces colored by k-means cluster (B,D,F,H,J).

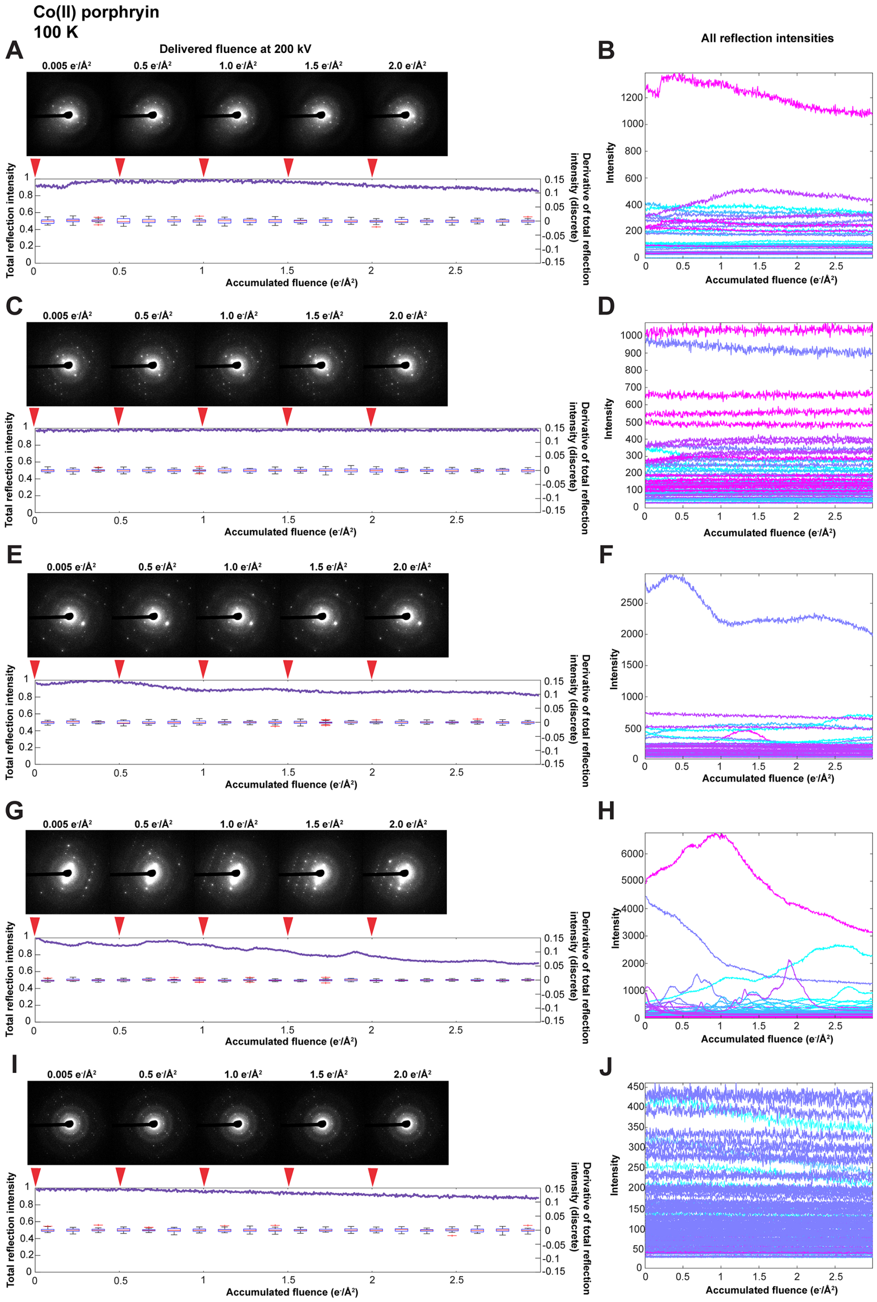

Figure S15. Changes in reflection intensity in response to electron beam exposure for stationary Co(II) porphyrin crystals at 200 kV and cryogenic (100 K) temperature. For five different crystals diffracted under the same conditions, initial diffraction pattern, and frames acquired following an accumulated fluence of 0.5, 1.0, 1.5, and 2.0 $e^-/Å^2$, from left to right, plot of normalized total reflection intensity (considering the brightest 20% of detected reflections, left-hand y-axis) as a function of accumulated fluence, and discretized plot of values of the derivative of this curve with respect to fluence (right-hand y-axis) (A,C,E,G,I). Plots of all reflection intensities for each diffraction series, with traces colored by k-means cluster (B,D,F,H,J).

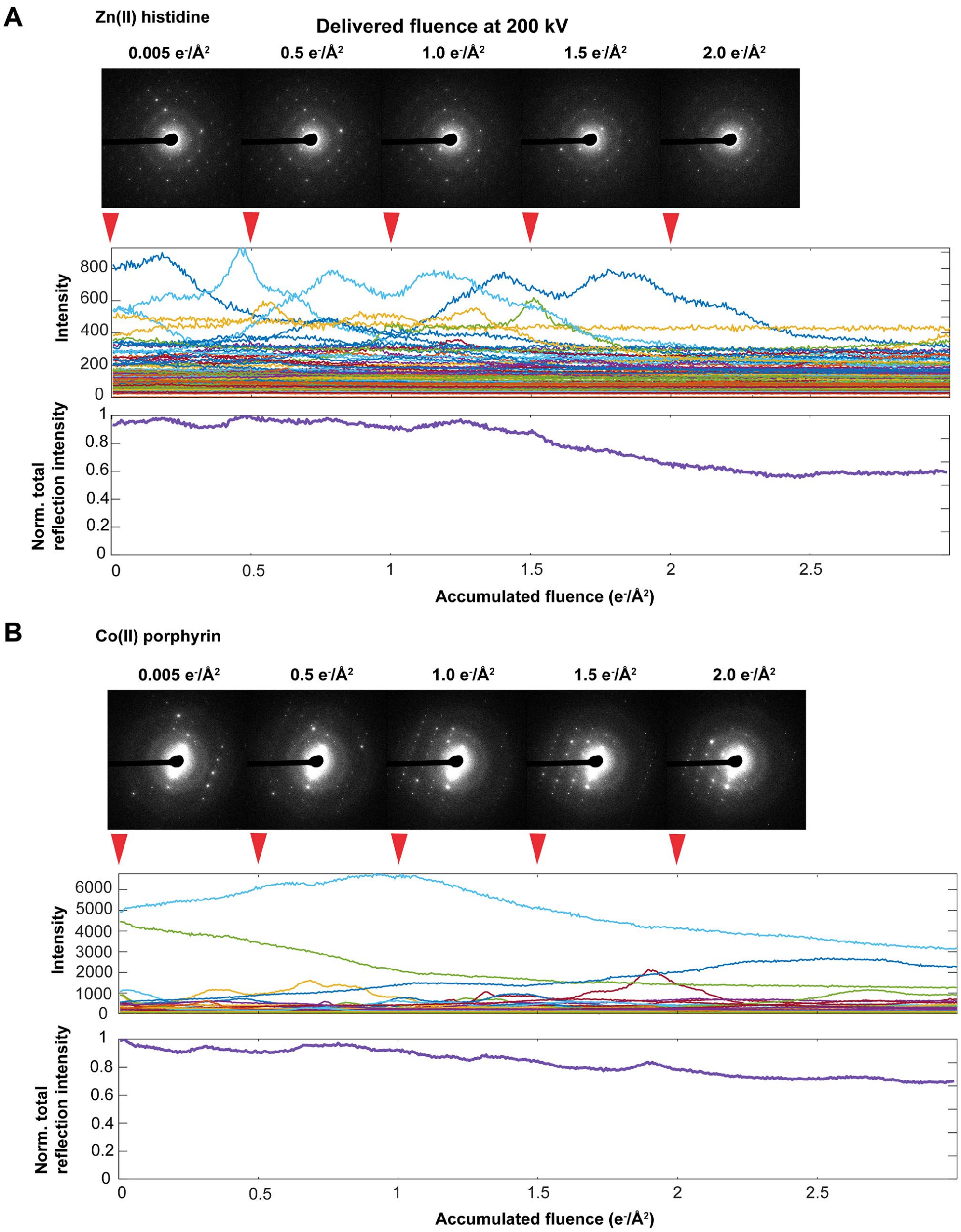

Figure S16. Instances of non-monotonic reflection intensity fluctuations in static Zn(II) histidine and Co(II) porphyrin crystals diffracted at cryogenic temperature (100 K). For a crystal of Zn(II) histidine (A) and a crystal of Co(II) porphyrin (B): initial diffraction pattern, and frames acquired following an accumulated fluence of 0.5, 1.0, 1.5, and 2.0 $e^-/Å^2$, from left to right, plot of all reflection intensities as a function of fluence, and plot of normalized total reflection intensity as a function of accumulated fluence. Note that these cases were not representative of the majority of Zn(II) histidine and Co(II) porhyrin crystals studied under these conditions; the majority showed mild to absent levels of non-monotonic decay, more akin to the examples in Fig. S10(E-G).

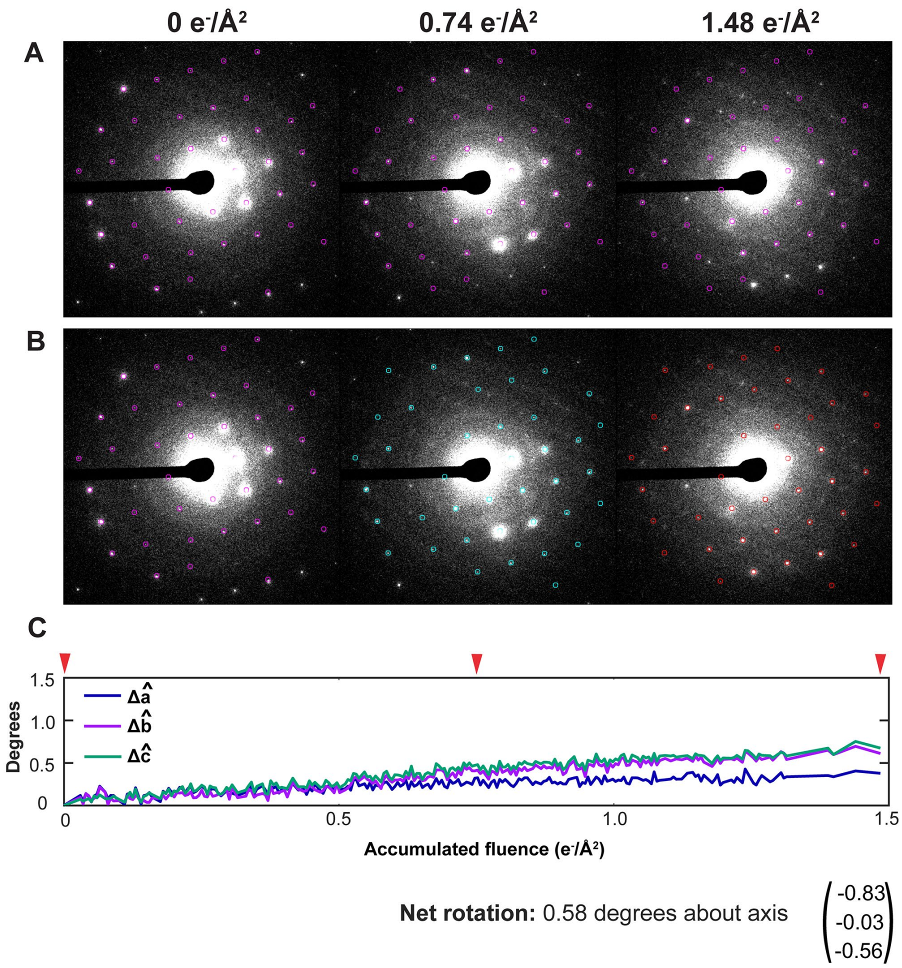

Figure S17. Tracking reorientation of a Zn(II) methionine crystal at 100 K. Diffraction frames from three points in a series on a stationary crystal (200 kV), overlaid with markers (magenta) at spot positions predicted for the orientation determined for the initial frame by *nXDS* (A). Predicted spot positions from orientations determined for each frame individually, (magenta, teal, and red markers), overlaid on the same three frames (B). Calculated angular change in each unit cell vector from its initial position as a function of fluence, and net rotation of the unit cell from its initial orientation following delivery of 1.48 $e^{-}/Å^{2}$ accumulated fluence (C). At 100 K, Zn(II) methionine crystals undergo smooth reorientations rather than abrupt crystal quakes.

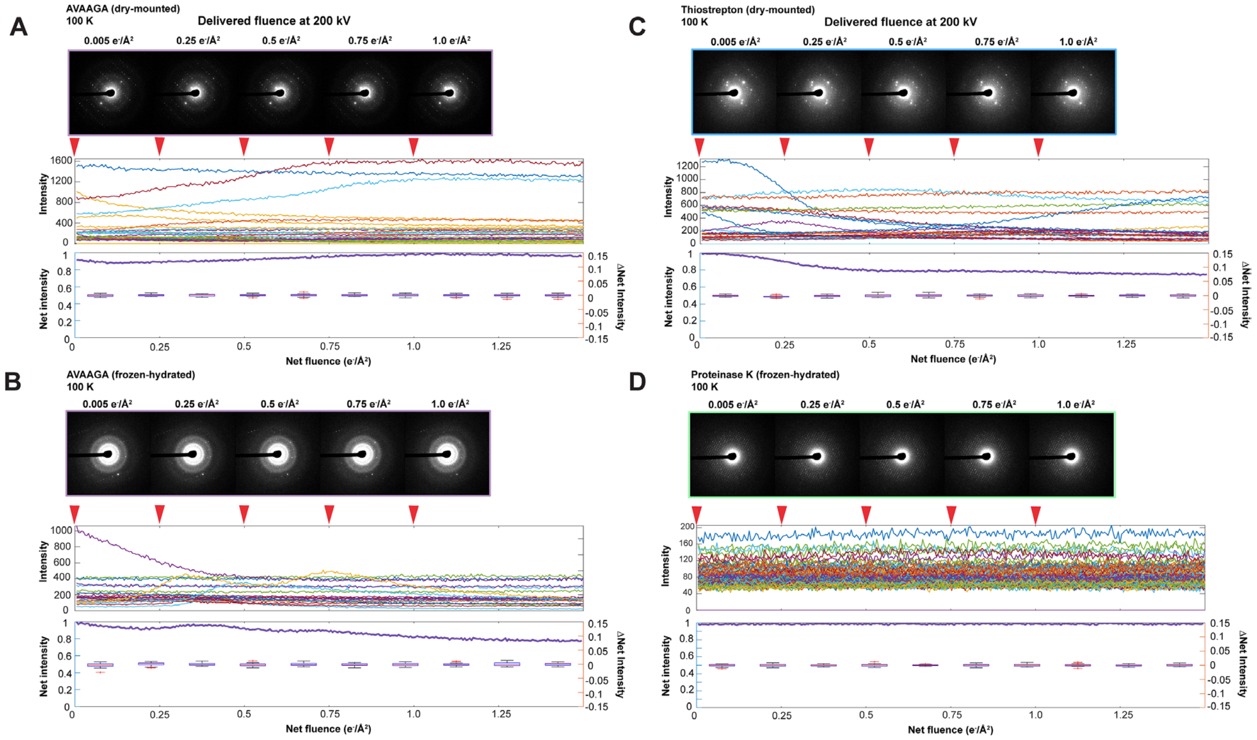

Figure S18. Changes in reflection intensity in response to electron beam exposure for stationary biomolecular crystals at 200 kV and cryogenic temperatures (100 K) . For a representative crystal each of AVAAGA (mounted dry on grids) (A), AVAAGA (embedded in vitreous ice) (B), thiostrepton (C), and proteinase K (D): initial diffraction pattern, and frames acquired following an accumulated fluence of 0.25, 0.5, 0.75, and 1.0 $e^{-}/Å^{2}$, from left to right. Beneath is a plot of the 20% brightest reflection intensities measured in the series as a function of fluence, and plot of normalized total reflection intensity (considering the brightest 20% of detected reflections, left-hand y-axis) as a function of accumulated fluence, and discretized plot of values of the derivative of this curve with respect to fluence (right-hand y-axis).

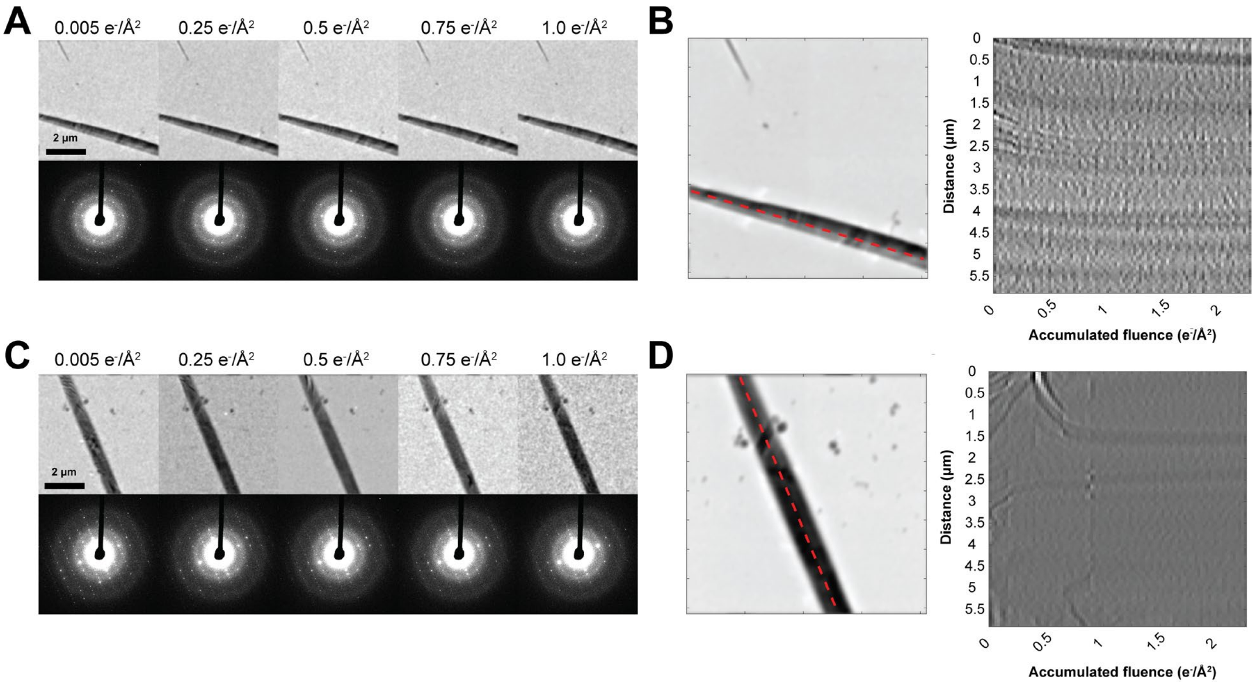

Figure S19. Beam-induced bend contour motion occurs in static crystals, either dry or vitrified, of the peptide AVAAGA at 100K. Images of dry (A) and frozen-hydrated (C) nanocrystals of AVAAGA following a delivery of increasing total fluence, alongside selected area diffraction patterns acquired immediately prior to each image. Following application of a bandpass filter in the time dimension of each image stack, projections of pixel intensities along manually defined directions (red dashed line) as a function of fluence (B and D).

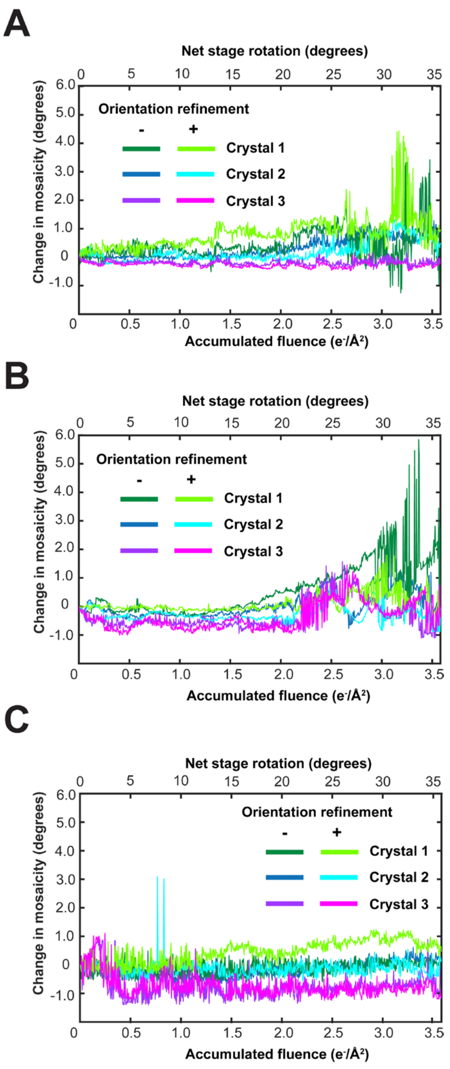

Figure S20. For biotin (A) and Zn(II) methionine crystals (B) at 100 K, and Co(II) porphyrin crystals at room temperature (C) all rotating at 0.09 degrees per second during diffraction data acquisition: plots of the change in mosaicity determined by *XDS* for each data frame, relative to the first frame, as a function of both accumulated fluence and rotation angle from the sample stage's initial position, for three representative crystals of each type, both with all geometric parameters held fixed during processing (dull colors) and with crystal orientation refined in batches corresponding to 2.5 degrees of rotation each (bright colors).

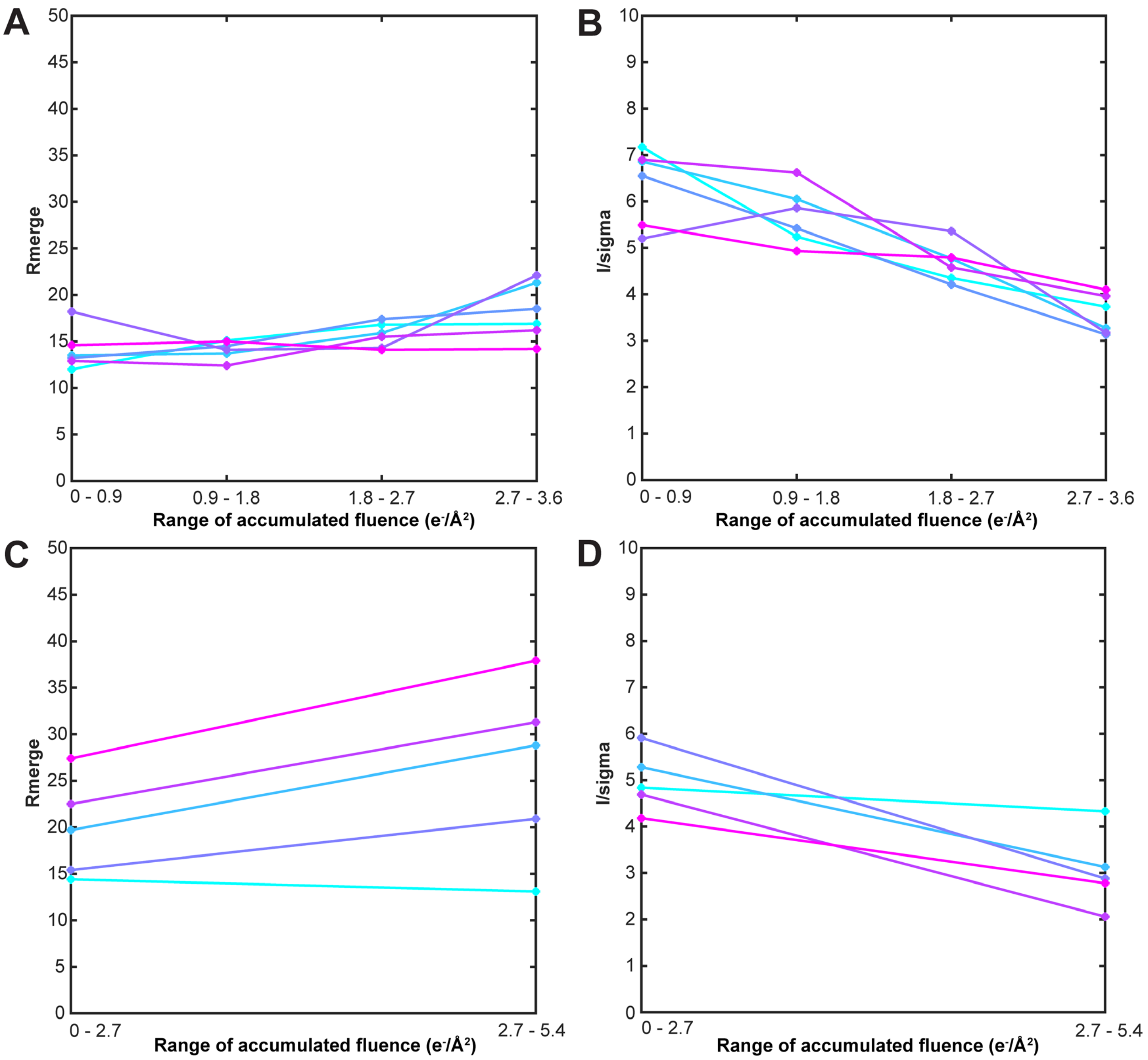

Figure S21. Data reduction statistics from processing successive sweeps of data collected on crystals of biotin at 100 K and 200 keV. Overall R_{merge} (A) and I/sigma (B) from 4 subsequent sweeps of data spanning the same 90 degree wedge of angular rotation with an incident flux of 0.01 $e^-/Å^2$, shown for 6 different crystals (different line and marker colors). Overall R_{merge} (C) and I/sigma (D) from 2 subsequent sweeps of data spanning the same 90 degree wedge of angular rotation with an incident flux of 0.03 $e^-/Å^2$, shown for 5 different crystals (2 of which did not yield a second sweep that could be readily indexed). All crystals were rotated at a speed of 1 degree/second, with integrated frames 1 second in duration.

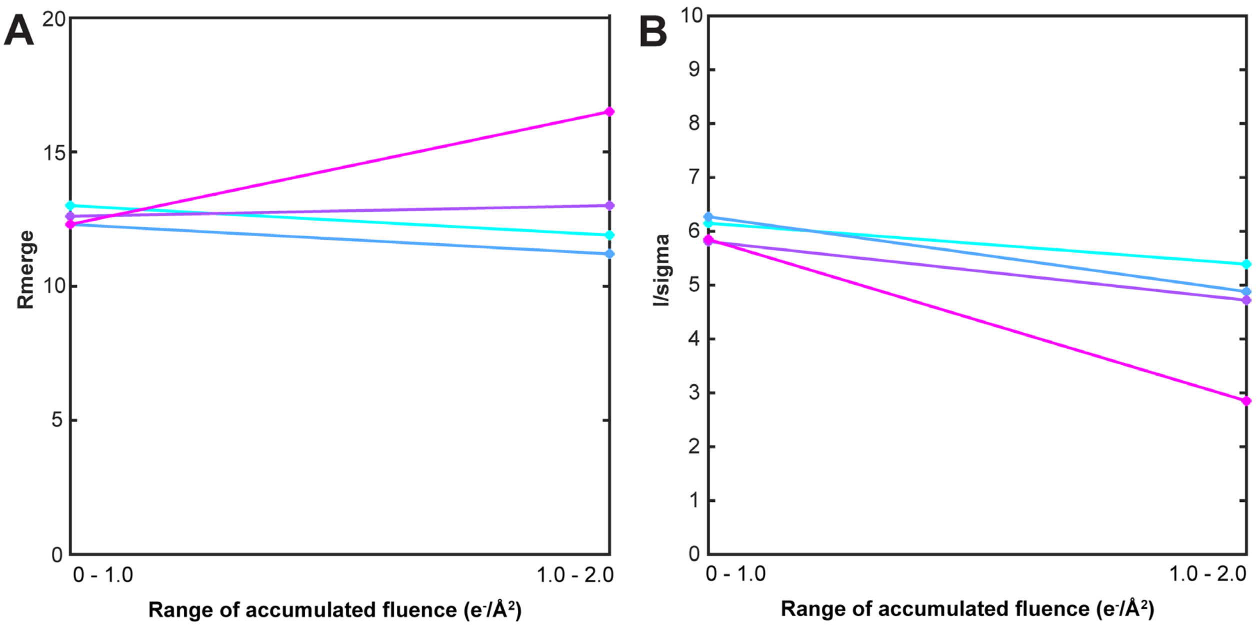

Figure S22. Data reduction statistics from processing successive sweeps of data collected on crystals of Zn(II) methionine at 100 K and 200 keV. Overall R_{merge} (A) and I/sigma (B) from 2 subsequent sweeps of data spanning the same 100 degree wedge of angular rotation with an incident flux of 0.01 $e^{-}/Å^{2}$, shown for 4 different crystals (different line and marker colors). All crystals were rotated at a speed of 1 degree/second, with integrated frames 1 second in duration.

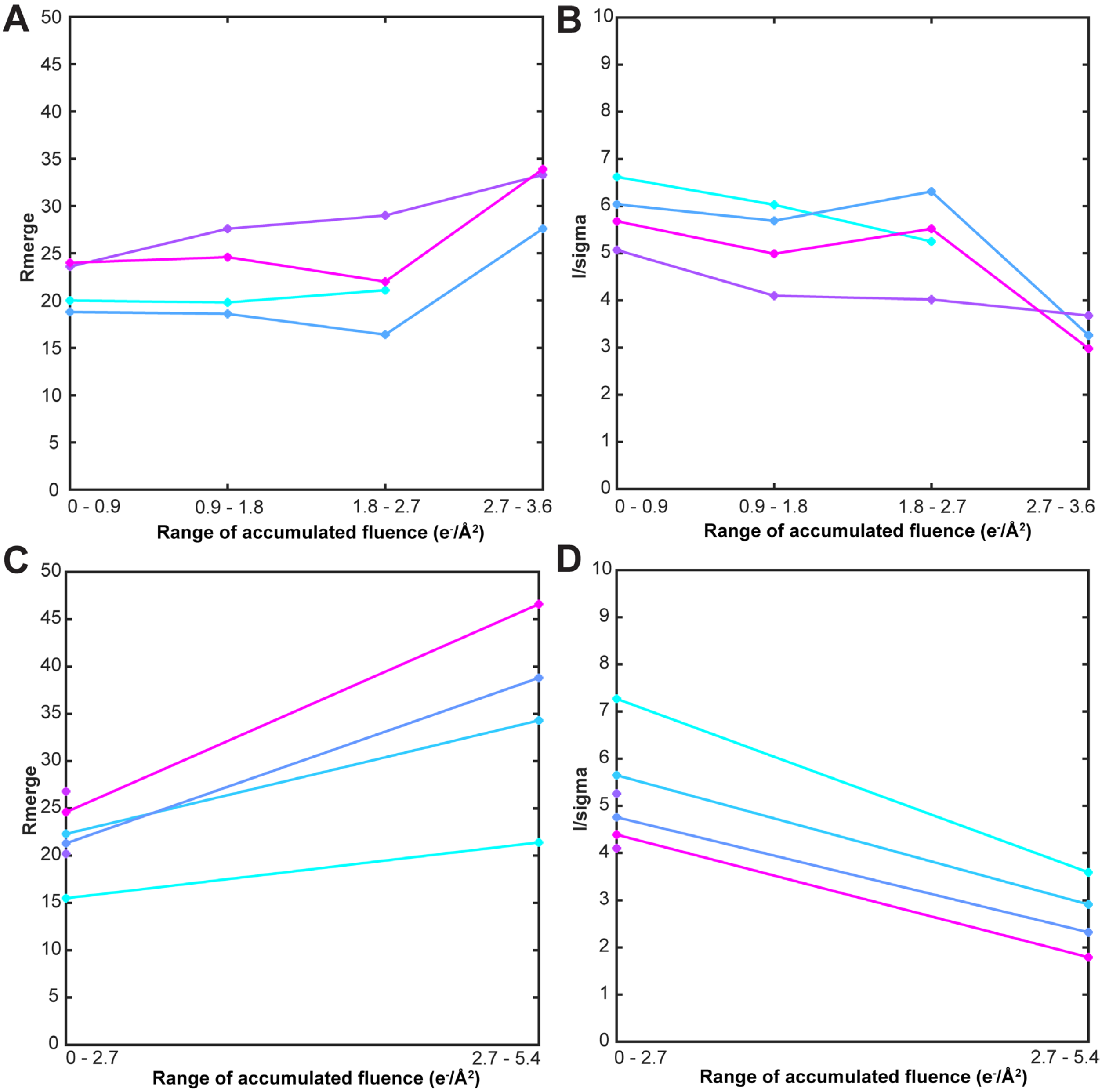

Figure S23. Data reduction statistics from processing successive sweeps of data collected on crystals of Zn(II) histidine at 293 K and 200 keV. Overall R_{merge} (A) and I/sigma (B) from 4 subsequent sweeps of data spanning the same 90 degree wedge of angular rotation with an incident flux of 0.01 $e^-/Å^2$, shown for 4 different crystals (different line and marker colors). Note data from the 4th sweep for one of these crystals could not be readily indexed. Overall R_{merge} (C) and I/sigma (D) from 2 subsequent sweeps of data spanning the same 90 degree wedge of angular rotation with an incident flux of 0.03 $e^-/Å^2$, shown for 6 different crystals (2 of which did not yield a second sweep that could be readily indexed). All crystals were rotated at a speed of 1 degree/second, with integrated frames 1 second in duration.

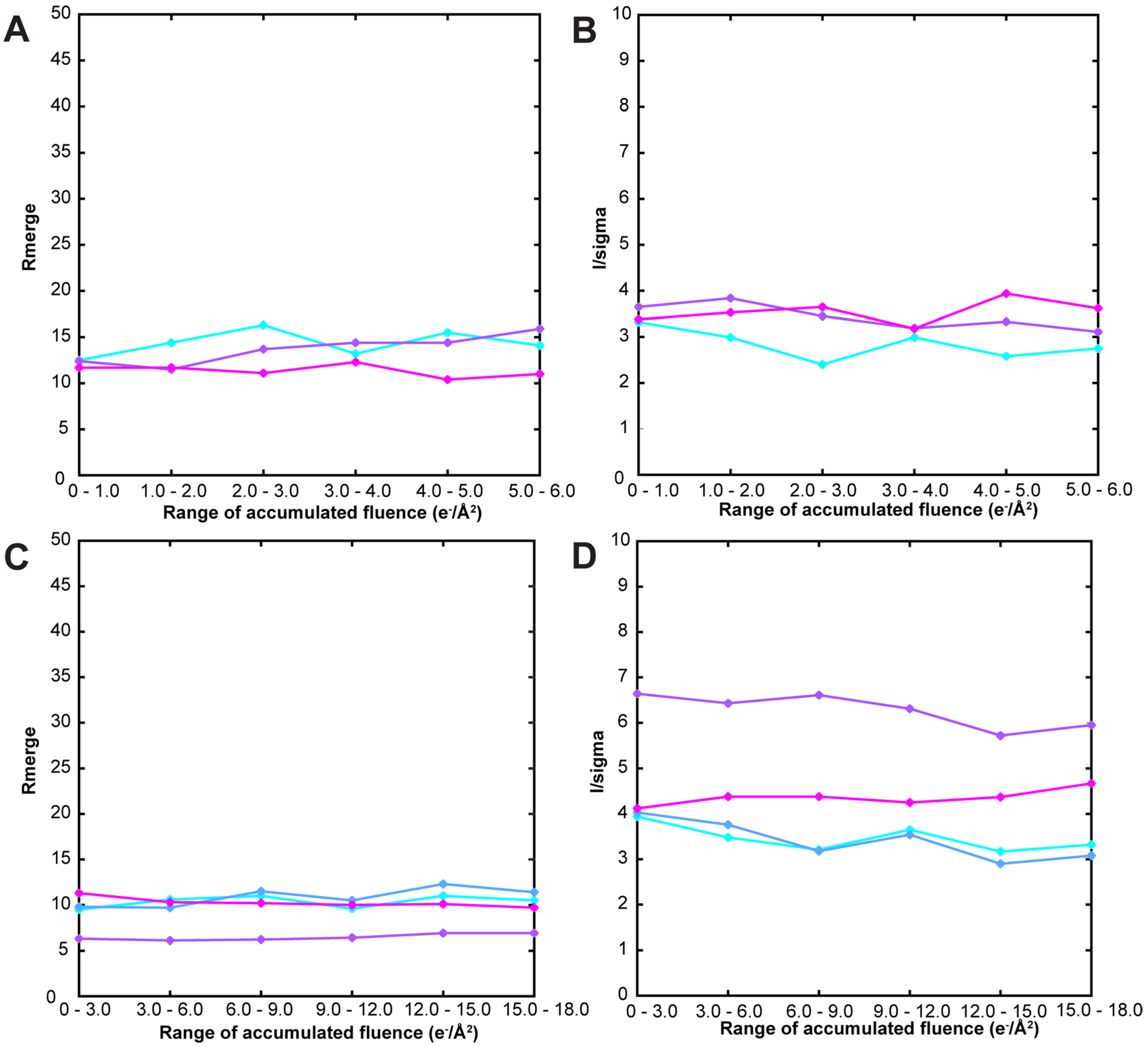

Figure S24. Data reduction statistics from processing successive sweeps of data collected on crystals of Co(II) porphyrin at 293 K and 200 keV. Overall R_{merge} (A) and I/sigma (B) from 6 subsequent sweeps of data spanning the same 100 degree wedge of angular rotation with an incident flux of 0.01 $e^-/Å^2$, shown for three different crystals (different line and marker colors). Overall R_{merge} (C) and I/sigma (D) from 6 subsequent sweeps of data spanning the same 100 degree wedge of angular rotation with an incident flux of 0.03 $e^-/Å^2$, shown for four different crystals. All crystals were rotated at a speed of 1 degree/second, with integrated frames 1 second in duration

Structure validation (CheckCIF) responses

Biotin

B-level alerts

```
RINTA01_ALERT_3_B  The value of Rint is greater than 0.18
            Rint given   0.181
PLAT020_ALERT_3_B The Value of Rint is Greater Than 0.12 .........      0.181 Report
```

An R_{int} value between 15-20% is not atypical for current microED datasets. Furthermore, merging of datasets from multiple crystals (4) afforded greater data completeness and redundancy, which improved structure refinement but with a penalty to merge R-factors, which were as low as 12% for individual crystals.

C-level alerts

```
PLAT082_ALERT_2_C High R1 Value ..................................       0.13 Report
PLAT084_ALERT_3_C High wR2 Value (i.e. > 0.25) ...................       0.33 Report
```

These R1 and wR2 values are typical for a current small molecule microED structure, and mirror the higher data reduction R-factors seen in microED data resulting from distortions/sources of inaccuracy present in data collection.

```
PLAT340_ALERT_3_C Low Bond Precision on  C-C Bonds ...............    0.00513 Ang.
PLAT355_ALERT_3_C Long   O-H (X0.82,N0.98A)  O3       - H11      .       1.01 Ang.
```

Data completeness is limited in microED datasets, which often leads to systematically missing/undersampled regions of reciprocal space, and resolution anisotropy in the corresponding direction of the final map. This is likely to reduce the precision of some bond lengths.

Cu(II) serine

B-level alerts

```
PLAT250_ALERT_2_B Large U3/U1 Ratio for <U(i,j)> Tensor(Resd    1)        5.4 Note
```

Data completeness is limited in microED datasets, which often leads to systematically missing/undersampled regions of reciprocal space, and resolution anisotropy in the corresponding direction of the final map. This is likely to yield some anisotropically stretched ADP ellipsoids, contributing to this high ratio.

C-level alerts

```
PLAT082_ALERT_2_C High R1 Value ..................................       0.12 Report
PLAT084 ALERT 3 C High wR2 Value (i.e. > 0.25) ...................       0.34 Report
```

These R1 and wR2 values are typical for a current small molecule microED structure, and mirror the higher data reduction R-factors seen in microED data resulting from distortions/sources of inaccuracy present in data collection.

```
PLAT341_ALERT_3_C Low Bond Precision on  C-C Bonds ...............      0.007 Ang.
```

Data completeness is limited in microED datasets, which often leads to systematically missing/undersampled regions of reciprocal space, and resolution anisotropy in the corresponding direction of the final map. This is likely to reduce the precision of some C-C bond lengths.

Zn(II) histidine

B-level alerts

```
RINTA01_ALERT_3_B  The value of Rint is greater than 0.18
            Rint given   0.181
PLAT020 ALERT 3 B The Value of Rint is Greater Than 0.12 .........      0.181 Report
```

Rint values are higher for current microED datasets than for XRD on similar targets (0.10 – 0.20 is typical), due to additional sources of inaccuracy present in data collection.

```
PLAT082_ALERT_2_B High R1 Value ..................................      0.17 Report
PLAT084_ALERT_3_B High wR2 Value (i.e. > 0.25) ...................      0.42 Report
```

These R1 and wR2 values are typical for a current small molecule microED structure, and mirror the higher data reduction R-factors seen in microED data resulting from distortions/sources of inaccuracy present in data collection.

C-level alerts

```
PLAT250_ALERT_2_C Large U3/U1 Ratio for <U(i,j)> Tensor(Resd    1)        2.4 Note
```

Data completeness is limited in microED datasets, which often leads to systematically missing/undersampled regions of reciprocal space, and resolution anisotropy in the corresponding direction of the final map. This is likely to yield some anisotropically stretched ADP ellipsoids, contributing to this high ratio.

```
PLAT341_ALERT_3_C Low Bond Precision on  C-C Bonds ...............    0.00975 Ang.
```

Data completeness is limited in microED datasets, which often leads to systematically missing/undersampled regions of reciprocal space, and resolution anisotropy in the corresponding direction of the final map. This is likely to reduce the precision of some C-C bond lengths.

Zn(II) methionine

B-level alerts

```
RINTA01_ALERT_3_B  The value of Rint is greater than 0.18
            Rint given   0.222
PLAT020_ALERT_3_B The Value of Rint is Greater Than 0.12 .........      0.222 Report
```

Data reduction R-factors are greater (typically 10-20%) for current microED datasets than for small molecule XRD datasets, due to additional sources of inaccuracy present in data collection. Merging of datasets from multiple crystals was necessary to achieve sufficient completeness for structure determination by direct methods, which incurred a penalty to merge R-factors.

```
PLAT084_ALERT_3_B High wR2 Value (i.e. > 0.25) ...................       0.38 Report
```

This wR2 value is typical for a current small molecule microED structure, and mirrors the higher data reduction R-factors seen in microED data resulting from distortions/sources of inaccuracy present in data collection.

C-level alerts

```
PLAT082_ALERT_2_C High R1 Value ..................................       0.15 Report
```

This R1 value is typical for a current small molecule microED structure, and mirrors the higher data reduction R-factors seen in microED data resulting from distortions/sources of inaccuracy present in data collection.

```
PLAT213_ALERT_2_C Atom O4              has ADP max/min Ratio .....        3.7 prolat
PLAT213_ALERT_2_C Atom C6              has ADP max/min Ratio .....        3.5 prolat
PLAT250_ALERT_2_C Large U3/U1 Ratio for <U(i,j)> Tensor(Resd    1)        3.1 Note
```

Data completeness is limited in microED datasets, which often leads to systematically missing/undersampled regions of reciprocal space, and resolution anisotropy in the corresponding direction of the final map. This is likely to yield some anisotropically stretched ADP ellipsoids, contributing to these high ratios.

```
PLAT341_ALERT_3_C Low Bond Precision on  C-C Bonds ...............    0.00667 Ang.
PLAT414_ALERT_2_C Short Intra D-H..H-X      H1B      ..H8B      .       1.94 Ang.
                                            2-x,-1/2+y,1-z  =      2_746 Check
PLAT420_ALERT_2_C D-H Bond Without Acceptor N1        --H1B      .     Please Check
```

Data completeness is limited in microED datasets, which often leads to systematically missing/undersampled regions of reciprocal space, and resolution anisotropy in the corresponding direction of the final map. This is likely to reduce the precision of some C-C bond lengths.

Co(II) tetraphenyl porphyrin

B-level alerts

```
THETM01_ALERT_3_B  The value of sine(theta_max)/wavelength is less than 0.575
           Calculated sin(theta_max)/wavelength =    0.5558
```

Reflections were most reliably observed out to 0.9 Angstrom resolution, and data reduction statistics were improved by only integrating reflections out to that shell. Therefore these were the reflections used for structure determination and refinement.

```
PLAT082_ALERT_2_B High R1 Value ................................       0.16 Report
PLAT084_ALERT_3_B High wR2 Value (i.e. > 0.25) ..................       0.45 Report
```

These R1 and wR2 values are typical for a current small molecule microED structure, and mirror the higher data reduction R-factors seen in microED data resulting from distortions/sources of inaccuracy present in data collection.

C-level alerts

```
RINTA01_ALERT_3_C  The value of Rint is greater than 0.12
           Rint given   0.137
PLAT020_ALERT_3_C The Value of Rint is Greater Than 0.12 .........      0.137 Report
```

Rint values are higher for current microED datasets than for XRD on similar targets (0.10 – 0.20 is typical), due to additional sources of inaccuracy present in data collection.

```
PLAT220_ALERT_2_C NonSolvent   Resd 1  C   Ueq(max)/Ueq(min) Range        4.0 Ratio
PLAT241_ALERT_2_C High   'MainMol' Ueq as Compared to Neighbors of         C3 Check
PLAT241_ALERT_2_C High   'MainMol' Ueq as Compared to Neighbors of         C7 Check
PLAT241_ALERT_2_C High   'MainMol' Ueq as Compared to Neighbors of         C8 Check
PLAT241_ALERT_2_C High   'MainMol' Ueq as Compared to Neighbors of        C29 Check
PLAT241_ALERT_2_C High   'MainMol' Ueq as Compared to Neighbors of        C30 Check
PLAT241_ALERT_2_C High   'MainMol' Ueq as Compared to Neighbors of        C43 Check
PLAT242_ALERT_2_C Low    'MainMol' Ueq as Compared to Neighbors of         C6 Check
PLAT242_ALERT_2_C Low    'MainMol' Ueq as Compared to Neighbors of        C28 Check
PLAT242_ALERT_2_C Low    'MainMol' Ueq as Compared to Neighbors of        C31 Check
PLAT242_ALERT_2_C Low    'MainMol' Ueq as Compared to Neighbors of        C42 Check
```

Data completeness is limited in microED datasets, which often leads to systematically missing/undersampled regions of reciprocal space, and resolution anisotropy in the corresponding direction of the final map. This is likely to yield some anisotropically stretched ADP ellipsoids, contributing to the high Ueq ratio. Additionally, evidence of disorder is present in two of the phenyl rings on this structure, yielding additional disagreement between certain carbon atoms' Ueq relative to their neighbors.

```
PLAT341_ALERT_3_C Low Bond Precision on  C-C Bonds ..............    0.01212 Ang.
```

Data completeness is limited in microED datasets, which often leads to systematically missing/undersampled regions of reciprocal space, and resolution anisotropy in the corresponding direction of the final map. This is likely to reduce the precision of some C-C bond lengths.
